# A closer look at the Azzolino collection

**DOI:** 10.1371/journal.pone.0283539

**Published:** 2023-04-12

**Authors:** Anna Lagerqvist Alidoost, Marei Hacke, Thea Winther, Tom Sandström

**Affiliations:** 1 Swedish National Archives, Preservation and Conservation, Stockholm, Sweden; 2 Swedish National Heritage Board, Heritage Laboratory, Visby, Sweden; University of Florida, UNITED STATES

## Abstract

The state of preservation of documents from the historically significant Azzolino collection at the Swedish National Archives has been investigated and analyses carried out of the iron gall inks. The collection shows varied levels of iron gall ink corrosion. An initial visual condition survey was followed by characterisation of the writing ink with XRF spectrometry on a selection of documents. The aim was to investigate whether ink composition could be related to author or geography, and in turn to level of ink corrosion, which could then serve as a basis for decisions on treatment options. Results indicate a relative purity of the inks in this collection in terms of high iron content and low levels of other elements, entailing that elemental analysis is not a good tool to predict potential deterioration of ink in single documents from this historical context. XRF-mapping showed a possibility for discerning authors by ink composition, contributing meaningful information to questions of attribution and historical context for these documents. A tendency for the ink of Queen Christina to contain more copper than inks from the other authors, and the indication that some inks contain calcium, may be of note for further study.

## Introduction

### Background and aims of the study

The Azzolino collection contains documents connected to the Swedish Queen Christina (1626–1689), who governed the country between 1650 to 1654 before abdicating and moving to Rome [1]. The documents of the collection were generally written between the years 1655 and 1690. The collection consists of 57 volumes including approximately 5000 sheets of handwritten text that is mainly produced using iron gall ink as a medium, which through its corrosive nature presents considerable challenges to both the preservation and accessibility of the collection. These volumes are now housed at the Swedish National Archives in Marieberg, Stockholm, with the reference ID: SE/RA/710003/04 and are partly accessible online through the national archive database [2].

The Azzolino collection’s historical significance and reported poor state led the Unit of Preservation and Conservation at the Swedish National Archives to carry out a condition survey through visual inspection. The purpose of the survey was to gain insight into the variation of conditions of documents within the collection, and to enable a prioritisation of documents in need of conservation treatment or improved storage. The iron gall inks were analysed on a selection of documents using XRF spectrometry in collaboration with the Heritage Laboratory of the Swedish National Heritage Board. The aim of the XRF analyses was to investigate the possibility of relating specific ink compositions to certain writers, geographic locations and/or condition of the document through degree of ink corrosion.

### The Azzolino collection–history

The Azzolino collection owes its name to the Roman cardinal Decio Azzolino, who became a close friend to the Swedish Queen Christina after she had abdicated from the Swedish throne in 1654, converted to Catholicism, and moved to Rome [3, 4]. Azzolino was her universal heir, but died just shortly after her in 1689. Their individual archives were joined and saved as part of the Azzolino family archive in Iesi, Italy, until 1925, when the Swedish government purchased the documents connected to the Queen–since then referred to as the Azzolino collection [5]. The collection presents a diversity of documents representing a variety of origins, uses and stages of iron gall ink corrosion. The collection contains private as well as official letters and agreements on paper and parchment, drafts of letters and literary works, protocols, financial accounts, drawings and bound books. The writers are foremost Queen Christina, cardinal Decio Azzolino and members of the queen’s administration, but also prominent individuals from different royal houses of Europe [5].

### Iron gall ink–composition and degradation

Iron gall inks produced throughout the centuries can display variations in composition and characteristics, potentially affecting the condition of the ink and the substrate (e.g. paper or parchment). The most basic ingredients in iron gall inks are iron (II) sulphate (ferrous sulphate), historically known as green vitriol or copperas, and tannin (often from oak galls) in water with a binder such as gum Arabic [6]. The actual mechanisms of the colour-forming process of iron gall ink still represent a field of ongoing research and the exact form of the compounds that we know under the name “iron gall” have not been determined [7]. The ink is, however, generally understood as containing a water-soluble ferrous tannate complex that oxidises to form insoluble ferric tannate as the ink dries. Degradation reactions are governed by exposure to air, light, heat and humidity, and they will always occur in natural ageing but can accelerate under certain conditions, for example if free iron ions are present. Iron bound in a stable complex does not cause any harm, and in theory the iron tannate complex is stable. However, depending on the ink recipe, there may be some excess acids or excess iron and the gradual breakdown of the ferric tannate complex may release some of the iron [8]. Such free iron ions can oxidise from ferrous to ferric form (iron (II) into iron (III)), a reaction accompanied by the production of free radicals such as superoxide and hydroperoxyl which will greatly increase the oxidation of the surrounding cellulosic substrate, i.e. the paper [9]. The oxidation of cellulose in turn will speed up acid hydrolysis because acidic groups form during the oxidation. Also, acid hydrolysis will reduce the iron back to its former iron (II) state so that it is free to oxidise again into iron (III), leading to the autocatalytic breakdown of the iron gall ink and its substrate.

It has been shown that gum Arabic in the ink and gelatin sizing of the paper can to some extent protect the paper fibers [10, 11]. In addition, studies demonstrate that the nature of the vitriol affects the degradation process and that the presence of copper could accelerate the degradation [12, 13], whereas the presence of zinc may provide a somewhat protective action [14].

### XRF analyses of iron gall ink

Multiple studies of iron gall inks have demonstrated that XRF analyses can be used to distinguish different hands or manuscripts according to the ink compositions [15–20]. This is mostly based on variations in the copper and zinc contents relative to iron, but other elements, such as manganese, potassium, aluminium, sulphur, silicon and lead may also be distinctive markers of separate inks. An ambition to not only identify components in iron gall ink, but to also quantify them with the help of XRF analysis, has played a part in several studies. In an investigation of a 14^th^ century breviary, Aceto et al. used standardless quantification through a freeware software provided by the International Atomic Energy Agency, IAEA [15]. Many inks in this study were rich in copper and zinc, with some copper and zinc weight percentage levels even exceeding those of iron.

Other studies have used quantification methods based on standards such as samples prepared with inks made from historical recipes with known compositions or samples of paper impregnated with a known quantity of metal sulphate solutions [16–19]. Belhadj et al. analysed 14^th^ century manuscripts from Chartres and, in agreement with the study of Aceto et al., showed high zinc to iron levels of 1.6–2 weight/weight ratio [18]. The analysis of black ink lines on wooden substrates from Stradivari’s workshop from the middle of the 17^th^ to the 18^th^ century showed ink compositions with zinc to iron ratios of 0–0.35 and copper to iron ratios ranging from 0–0.5 [19]. Hahn et al. analysed manuscripts from authors including Goethe and Mozart from the late 18^th^ and early 19^th^ century, which displayed widely varying ink compositions with copper to iron ratios between 0 to >2 and zinc to iron ratios between 0 to 0.7 [17].

Another approach is to report semi-quantitative results using ratios of elements to iron, obtained through the net peak areas of XRF spectra. For example, in the study of paper manuscripts from Stradivari’s workshop from the late 17^th^ and early 18^th^ century, the measured copper and zinc to iron ratios based on XRF net peak areas were in the range of 0–0.25 [21].

## Materials and methods

### Condition survey

**Fig 1:**
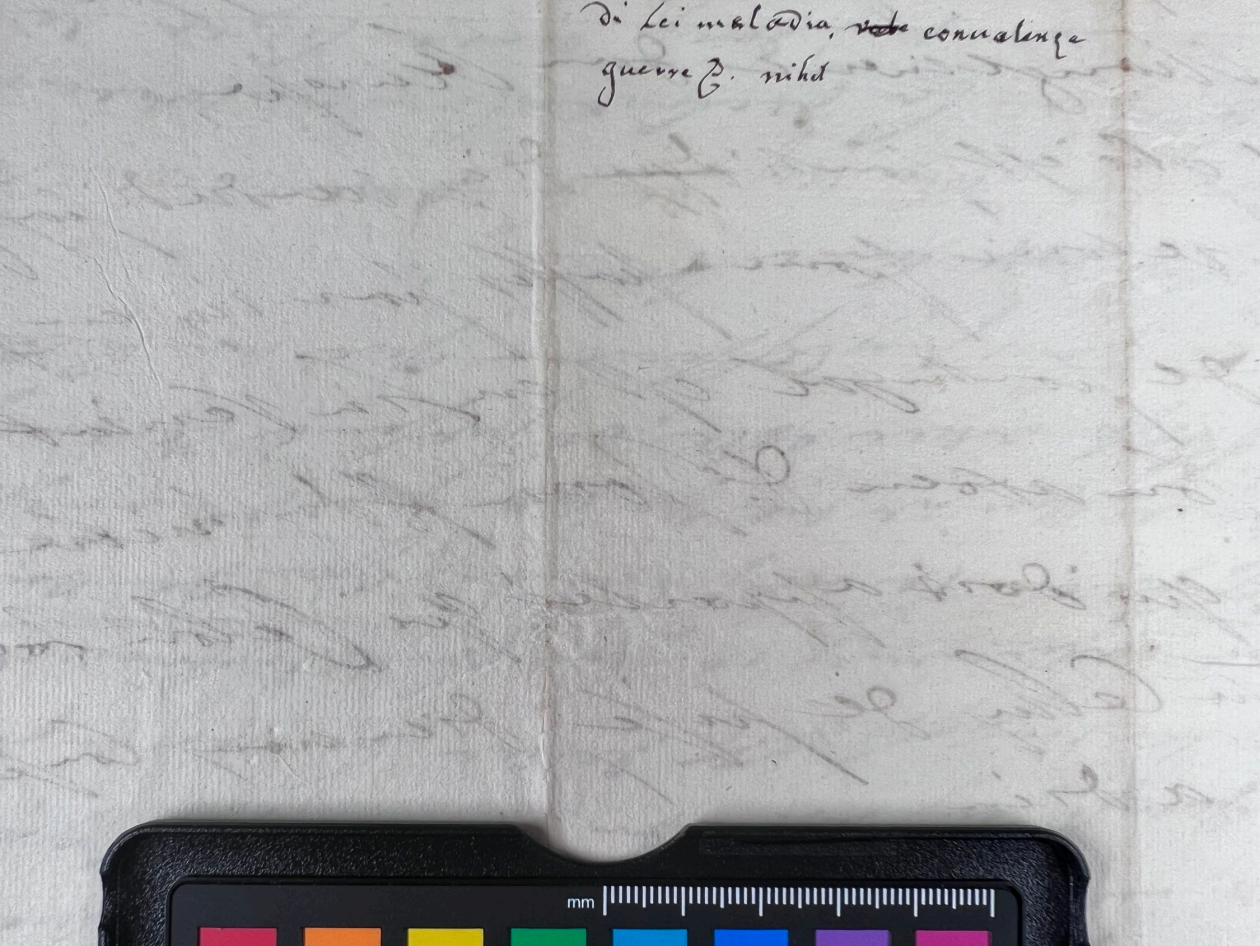
Burn through category 1. Example of document from the Azzolino collection displaying burn through 1 from verso.

**Fig 2:**
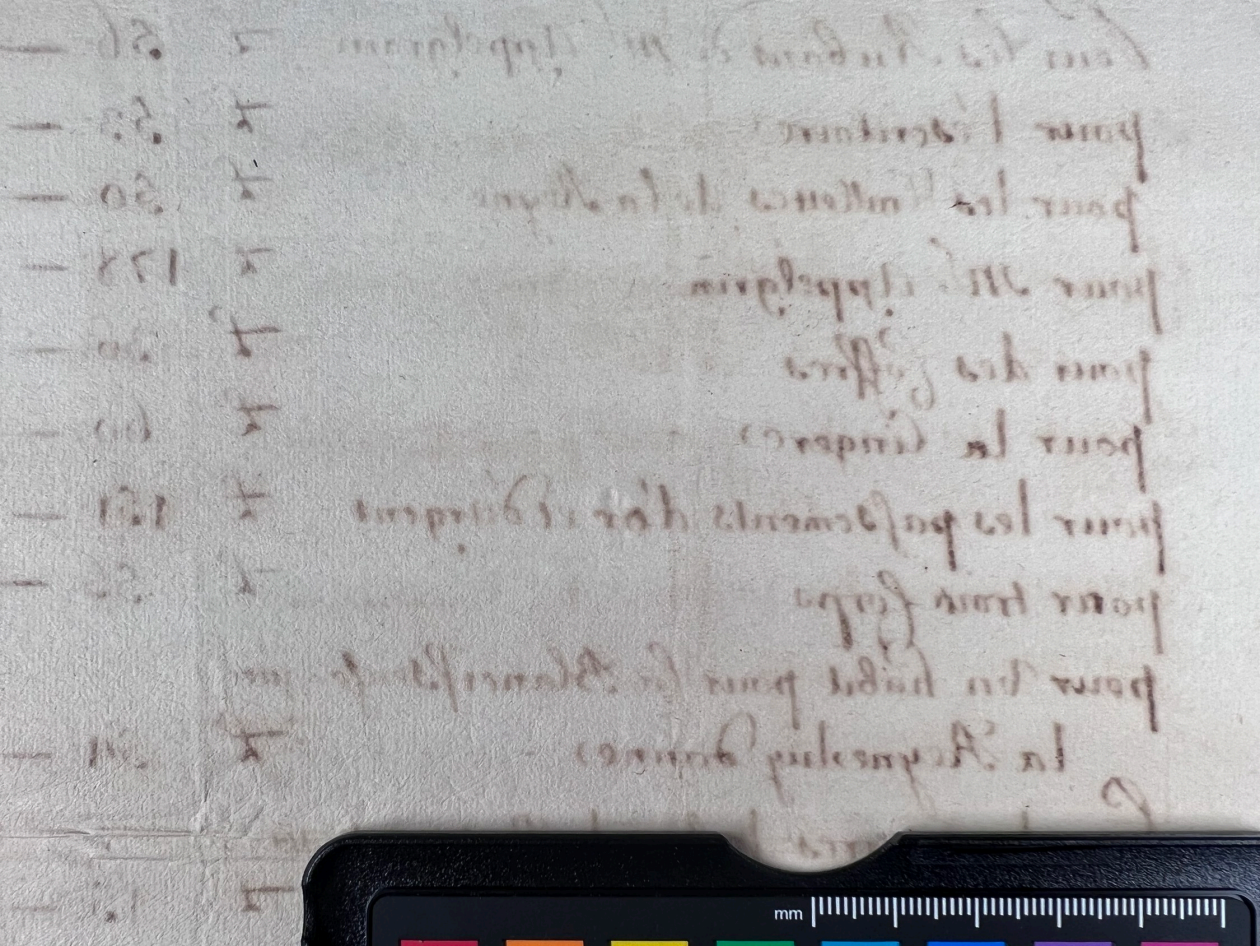
Burn through category 2. Example of document from the Azzolino collection displaying burn through 2 from verso.

**Fig 3:**
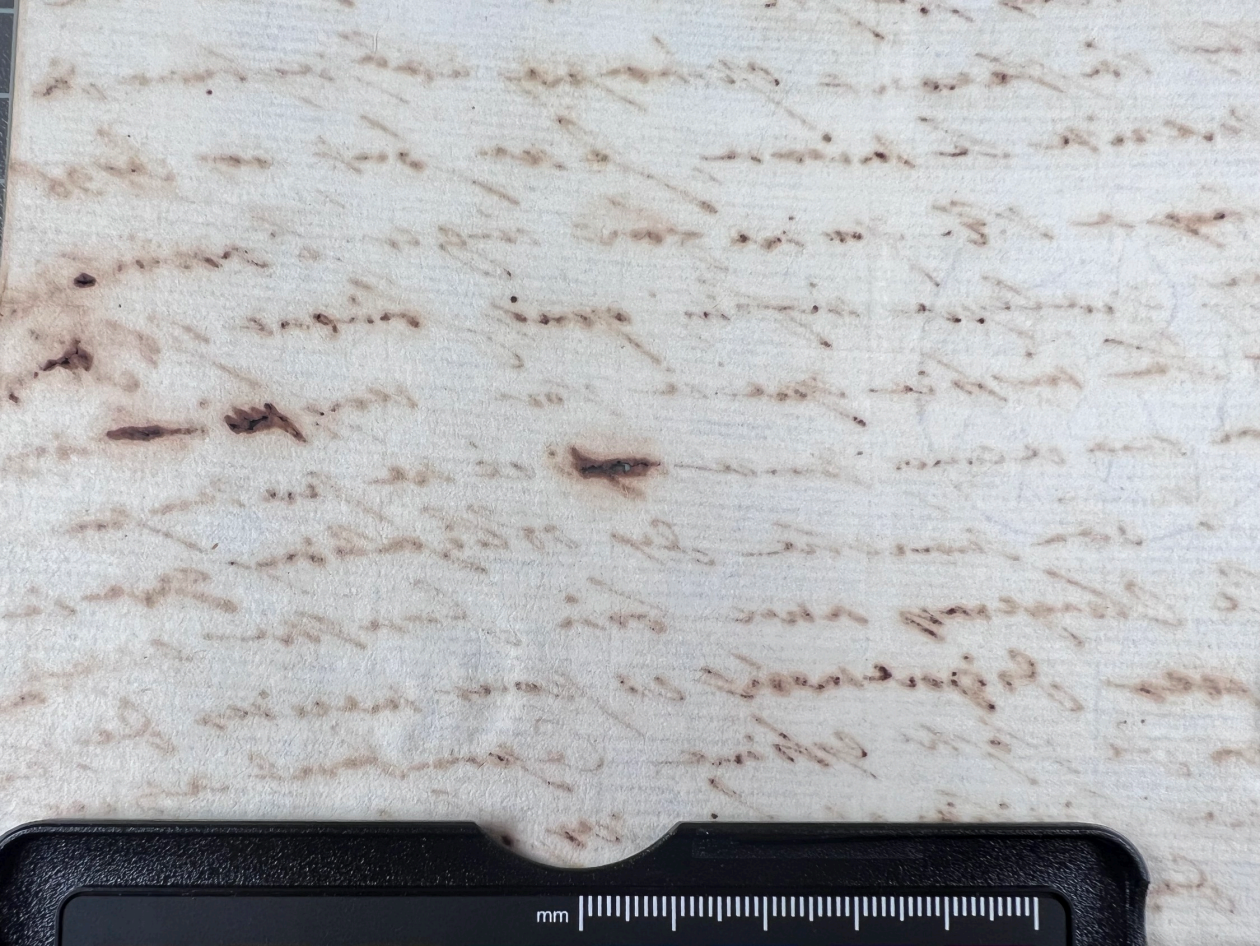
Burn through category 3. Example of document from the Azzolino collection displaying burn through 3 from verso.

**Fig 4:**
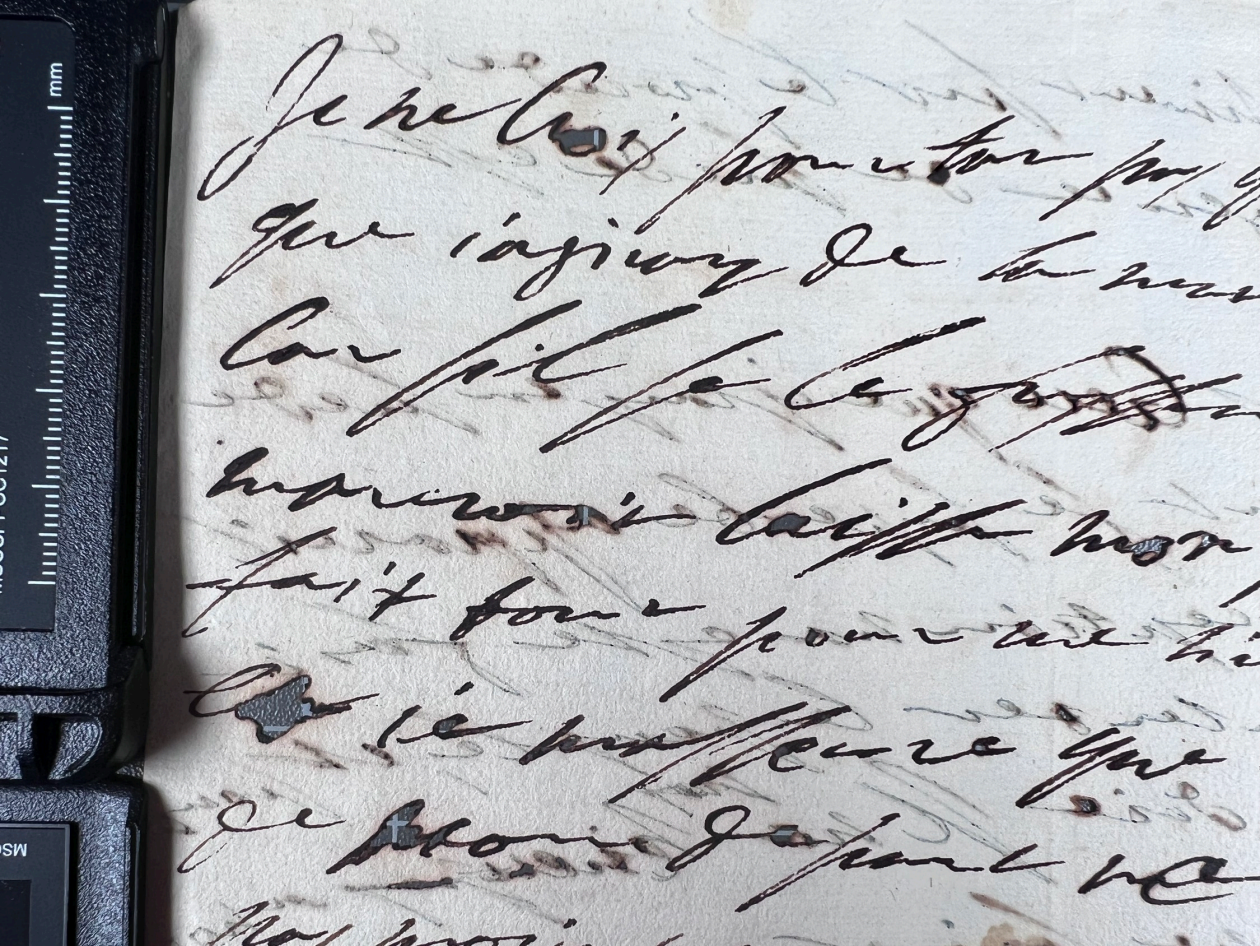
Burn through category 4. Example of document from the Azzolino collection displaying burn through 4, the document has text on both recto and verso.

The documents of the Azzolino collection currently present a complex set of preservation-related issues. Iron gall ink corrosion, along with mechanical and microbiological damage, can be found throughout the entire collection, but some parts display a condition so poor that the risk of information loss requires action. An initial condition evaluation of a statistical selection of the collection was carried out, see (S1 File). This followed a protocol constructed according to a slightly modified version of “Condition rating for paper objects with iron-gall ink” by Reißland and Hofenk de Graaff (2001) as well as recommendations from *The Iron Gall Ink Website* and the Library of Congress [22–24]. The degree of iron gall ink corrosion was evaluated from the verso of the document and categorised according to four condition ratings defined as categories of burn through from 1 to 4 (Table 1 and Figs 1–4). When text was written on both sides of a paper, the evaluation of burn through entails an estimation of the corrosion rate of the ink from both recto and verso. The condition rating categories from 2001 were slightly modified by adding the possible occurrence of a single crack in an inked area to the definition of burn through 2. This new description made it clearer how the overall degree of burn though for each document should be categorised.

**Table 1:** Categories of burn through.

Burn through 1	The document is in good condition with no or only light discoloration in inked areas.
Burn through 2	The document is in fair condition with dark brown discoloration in inked areas. No extensive mechanical damage, but possibly a singular occurrence of crack in an inked area.
Burn through 3	The document is in poor condition with mechanical damage through several cracks in inked areas.
Burn through 4	The document is in bad condition and displays serious loss of substance.

Degree of ink corrosion evaluated through categories of burn through; categories generally defined according to Reißland and Hofenk de Graaff [22].

In order to assess the condition of the entire collection of 4418 documents, with some documents consisting of multiple sheets, a statistical selection of every 14th document was made, leading to 314 surveyed documents. The method is described in the (S1 File) and is based on previous examples within condition surveying of library and archival materials, where methods for a randomized statistical selection are used for surveys of over 30 items but less than 10% of the population to represent the whole with 95% confidence and a margin of error of circa 5% [25–27].

**Fig 5:**
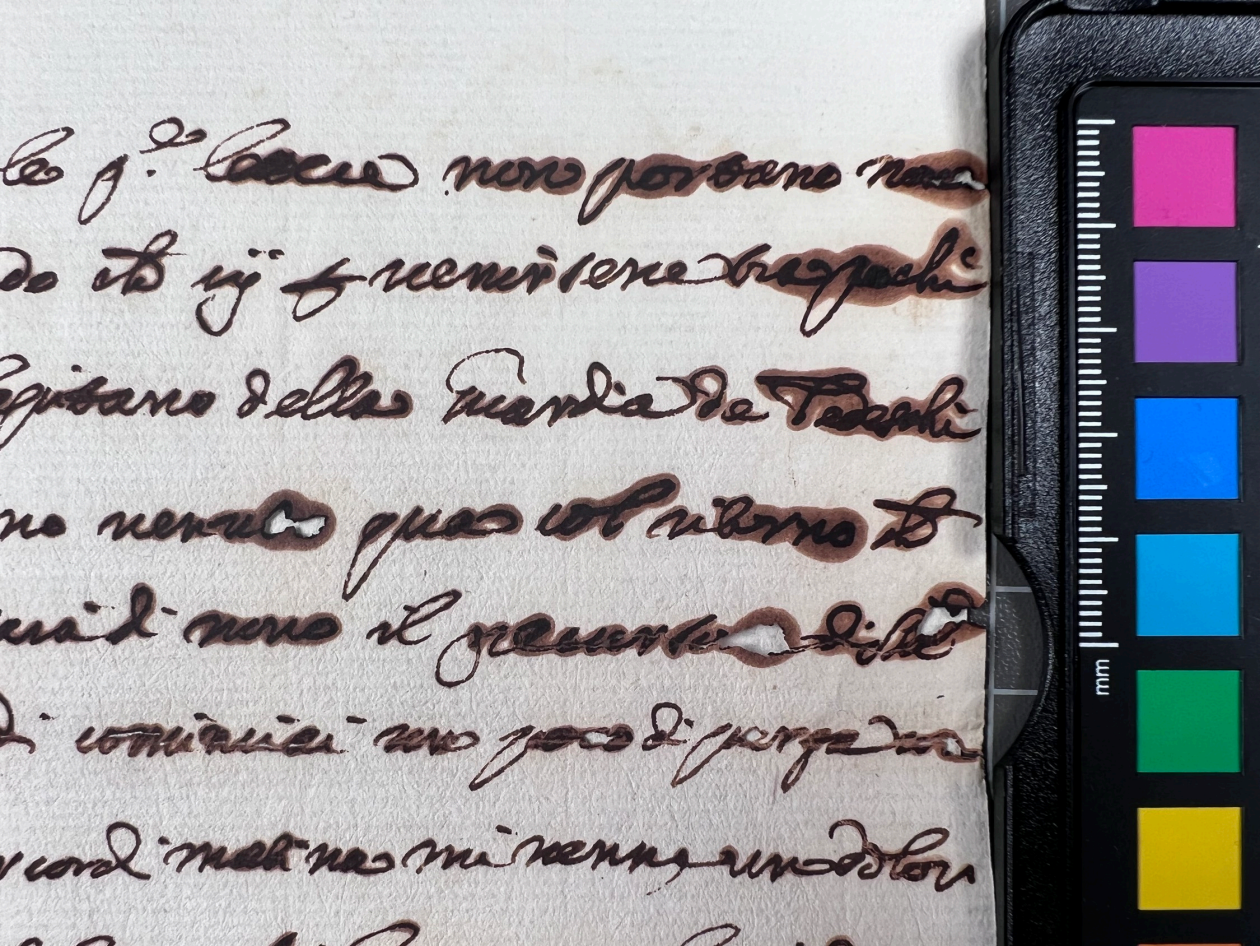
Ink halos. Ink halos due to ink corrosion. This particular document from volume K 400 of the Azzolino collection.

**Fig 6:**
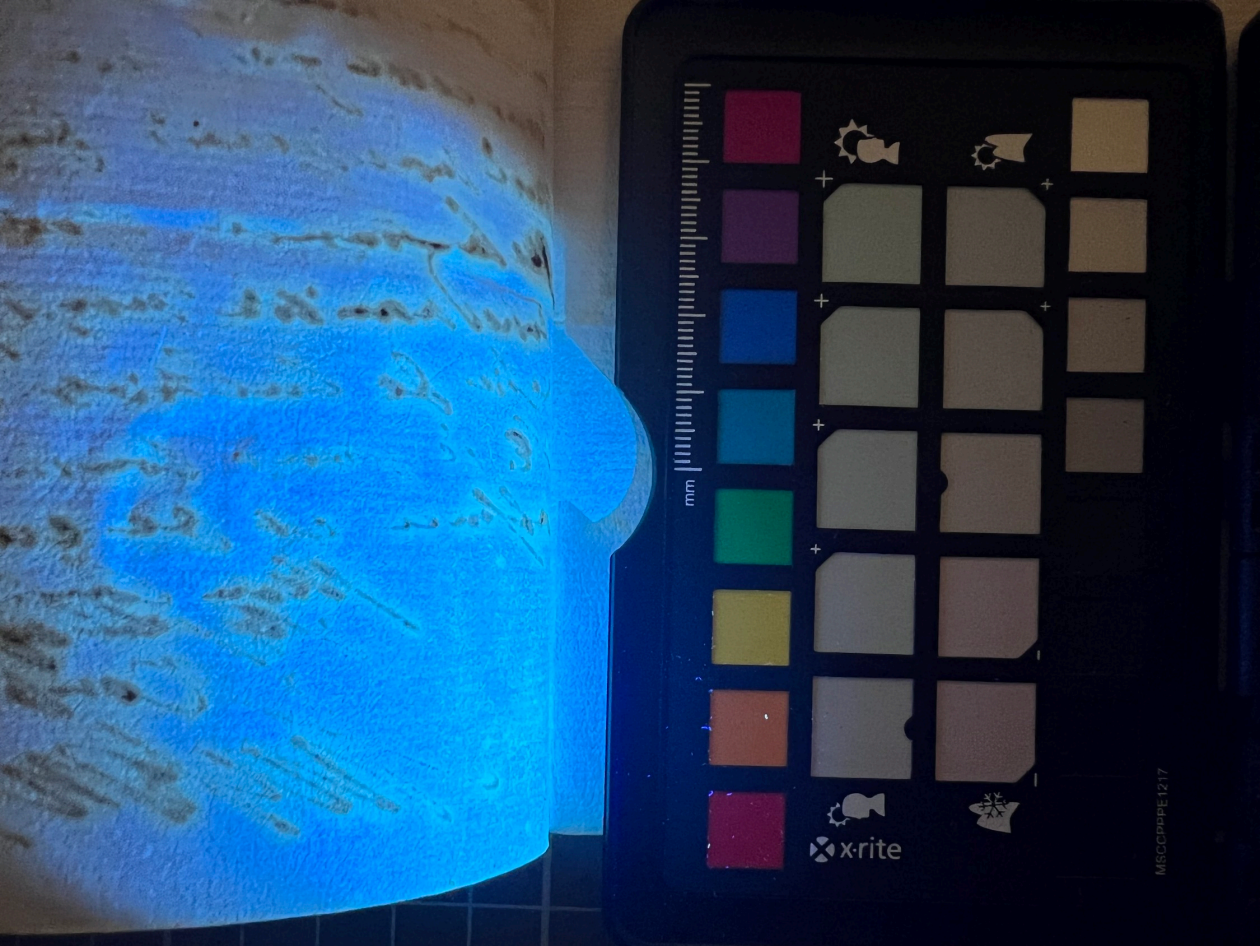
Fluorescence. Yellow fluorescence on the verso of corroding ink when viewed under UV light. This particular document from volume K 400 of the Azzolino collection.

**Fig 7:**
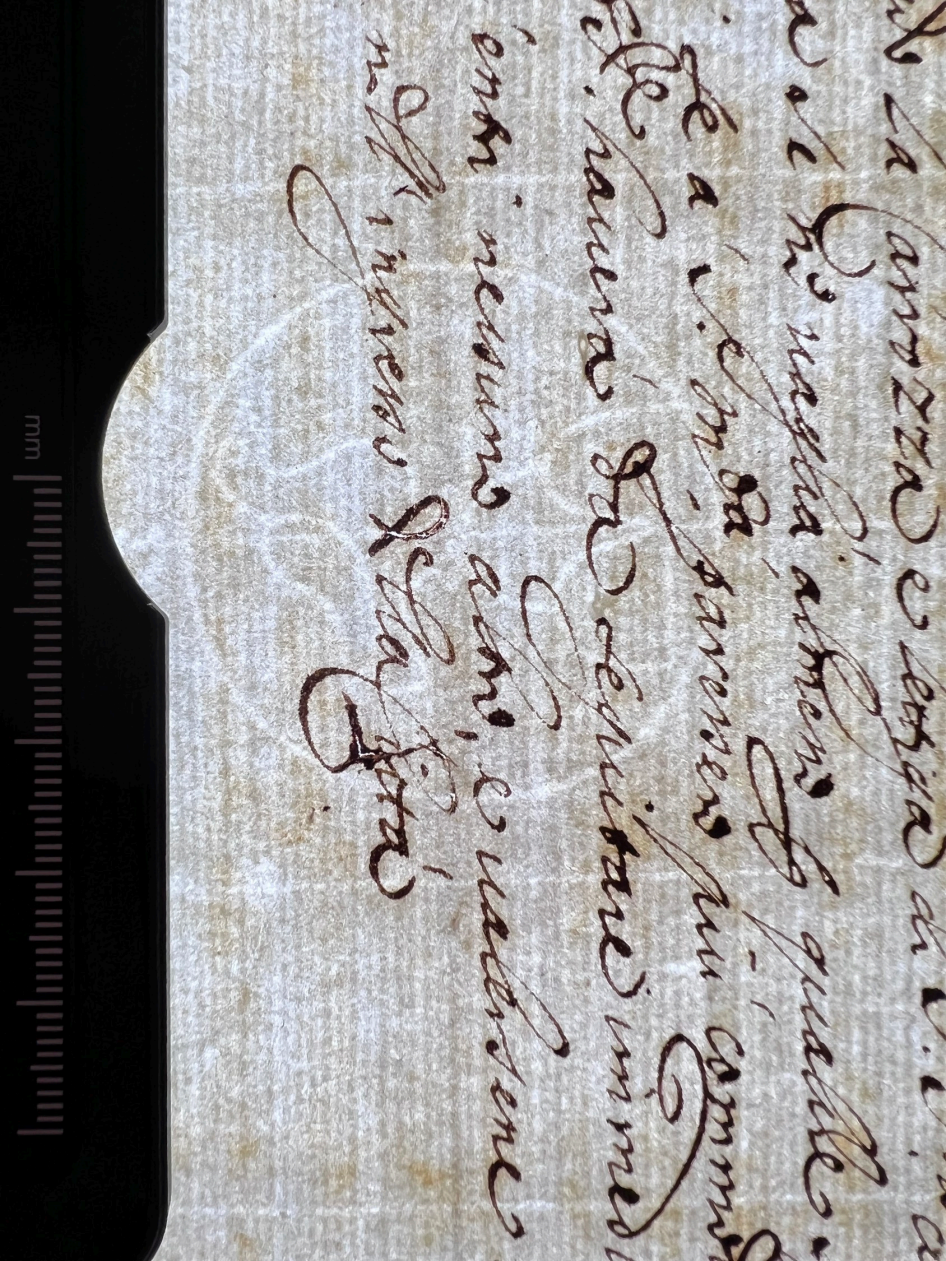
Cracks and watermarks. Cracks in ink and watermark in paper visualized through transmitted light. This particular document from volume K 400 of the Azzolino collection.

**Fig 8:**
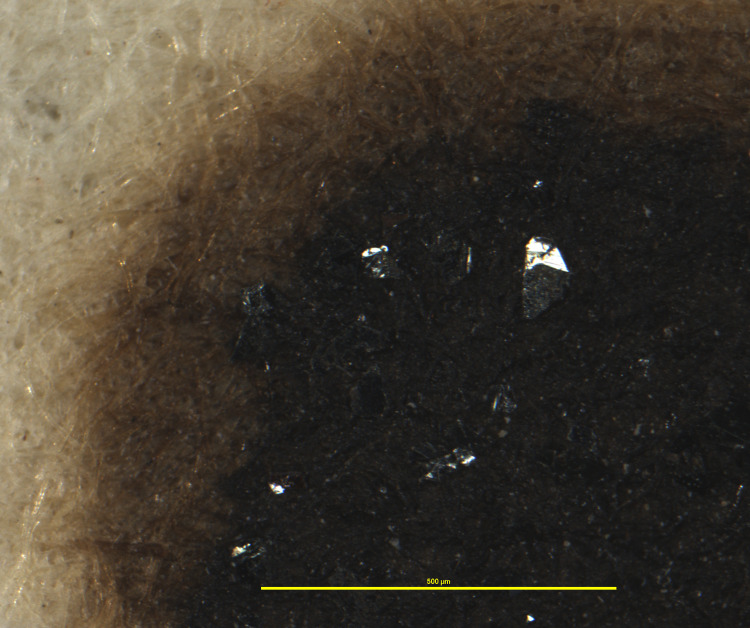
Ink sediments with reflective surfaces. Example of different sediment structures found on inks in the Azzolino collection, here black deposits with smooth, shiny surfaces. This particular document from volume K 427 of the Azzolino collection.

**Fig 9:**
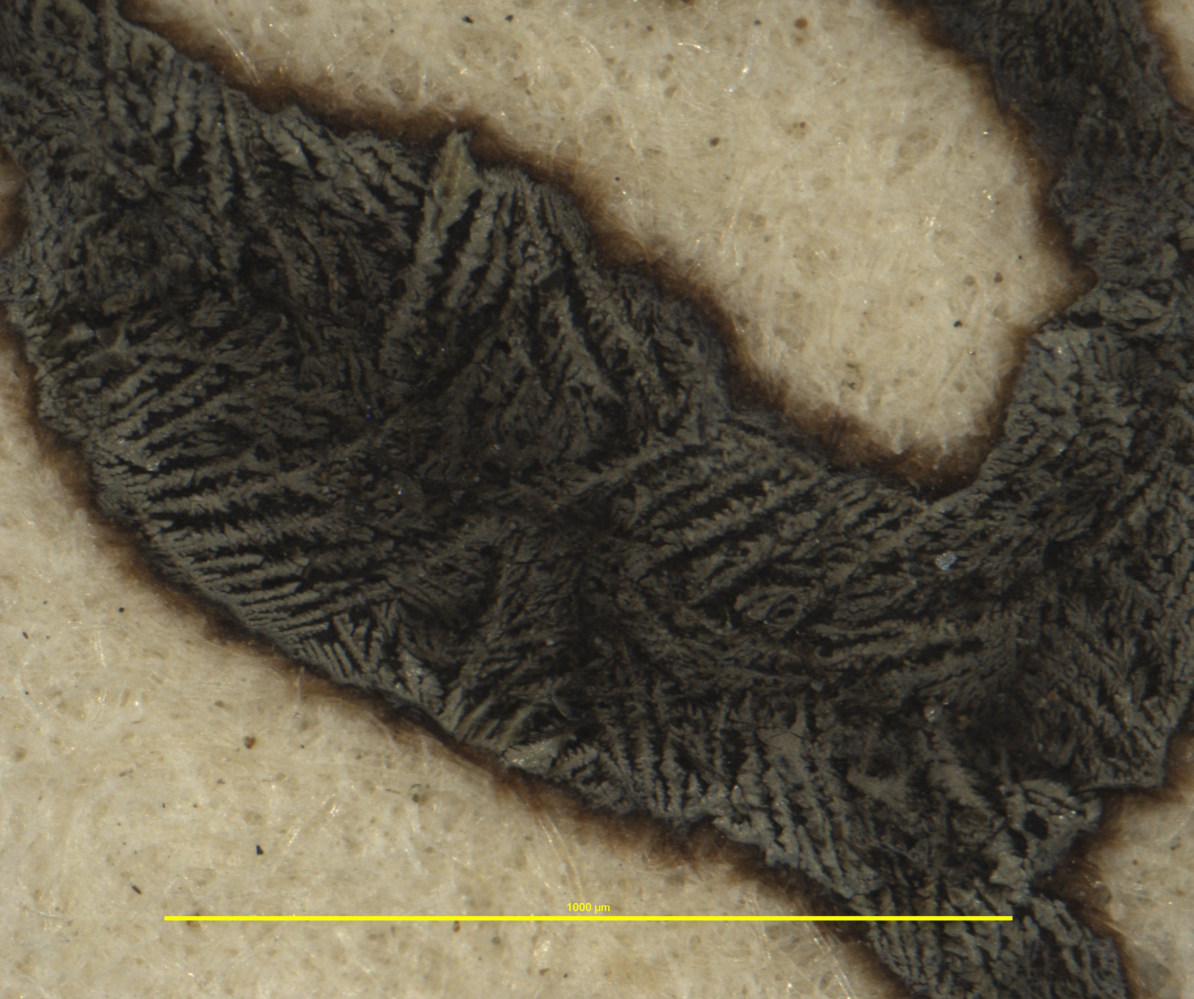
Feather-like sediments on ink. Example of different sediment structures found on inks in the Azzolino collection, here with a feather-like structure and a dull surface. This particular document from volume K 427 of the Azzolino collection.

**Fig 10:**
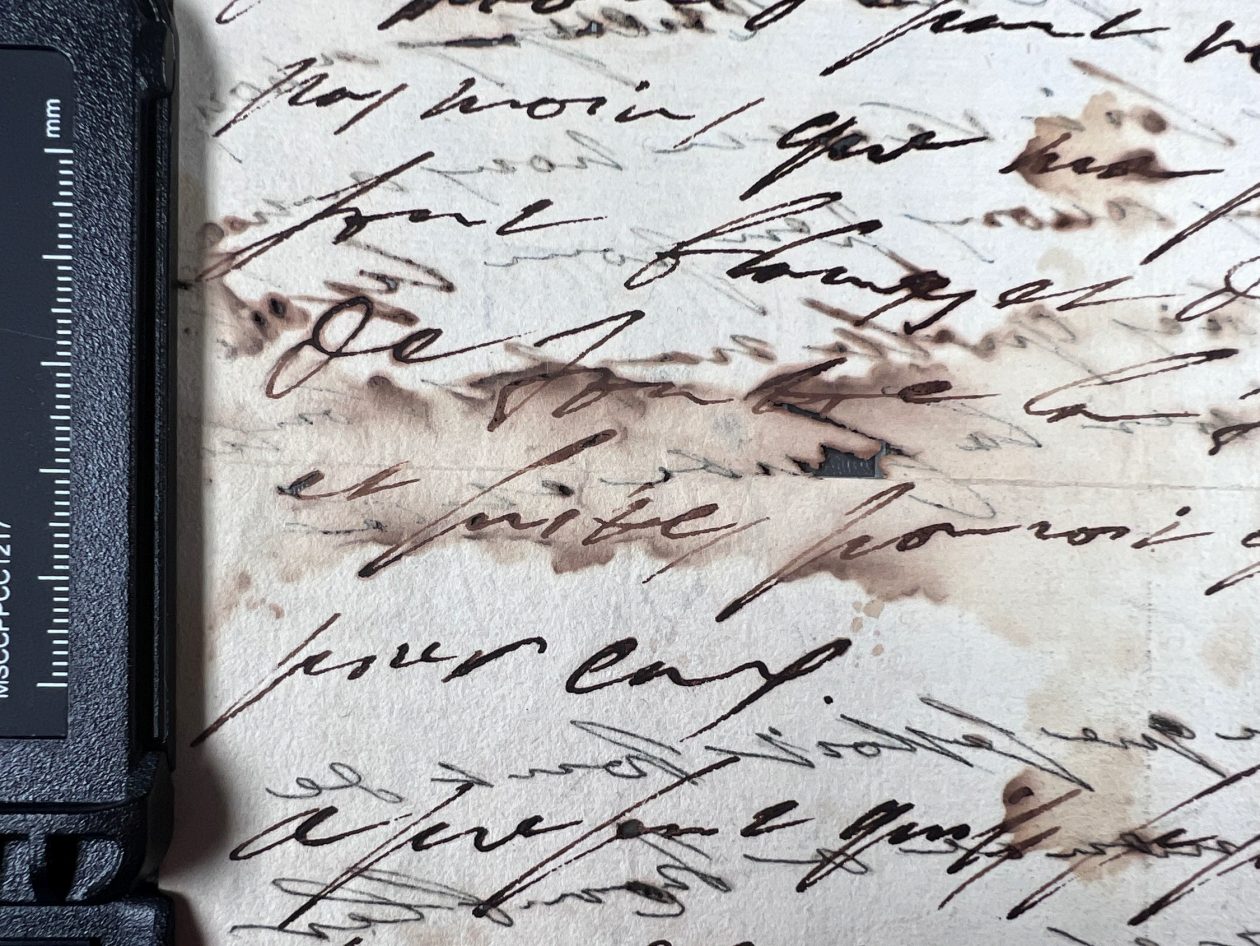
Water damage. Example of documents displaying effects from water damage. This particular document from volume K 400 of the Azzolino collection.

As individual documents of collections within the Swedish National Archives do not generally have specific numbers of identification, the documents of the Azzolino collection were counted throughout the survey process and given an informal ID according to the volume they belong to and the order in which they are kept. Apart from burn through, additional properties of the documents were examined, such as: the extent of ink coverage; whether or not the ink had been affected by water damage; possible water marks; the extent of ink-transfer from adjacent text; ink-transfer technique; thickness of ink layer; colour of UV fluorescence; presence of drying material or sediments and cracks (Figs 5–10). The documents were examined under a microscope (Olympus SZ4045 stereo microscope 10-20x and Dino-Lite AM7915MZT 10-160x), by raking light, UV light (365 nm) and transmitted light, and the presence of water marks was documented.

### XRF

A selection of 23 documents from the Azzolino collection, as well as one document from the so-called Del Monte collection, were selected for ink analysis using XRF. Both the Azzolino and Del Monte collections are accessible at the Swedish National Archives in Marieberg, Stockholm, and all necessary permits were obtained from the head of collections for the described study, which complied with all relevant regulations. The Del Monte document was included as questions had been raised regarding its authenticity as a letter written by Queen Christina, since the handwriting and content of the letter have, by some historians, been described as uncharacteristic [28]. The Azzolino documents were selected based on writer (primarily queen Christina, cardinal Azzolino and the queen’s banker, Manuel Teixeira), geographic location from where it was written (primarily Rome, Hamburg or Stockholm) and characteristics or symptoms of degradation or damage (such as thickness of ink lines, degree of ink corrosion, UV fluorescence, water damage, ink halos and previous conservation treatments). The overall condition of the individual documents was estimated and defined through categories of good, fair and poor, thus relating degrees of ink corrosion to the general condition of the paper object. The documents were written within a time span of 30 years, from 1659 to 1689, and represent a variety of origins and ink properties corresponding with the collection as a whole. The XRF work was intended as a pre-study to judge the feasibility of using the technique for verifying, or possibly even quantifying, differences between the inks and their inherent links to other variabilities.

The XRF instrument used was an Artax 800 (Bruker, Berlin, Germany), with a molybdenum X-ray tube and polycapillary lens (measuring spot size <100μm) at 50 kV and 600 μA current. Spectra were collected in air and without filters. Further details about instrument settings and areas of analysis can be found in the (S2 and S3 Files).

Elemental 2D mapping was carried out on four of the documents. Each mapped area measured approximately 1 cm x 2 cm. Mapping result images give relative quantitative information in greyscale for every element detected, where black represents the lowest levels detected and white indicates the highest levels. The minimum and maximum net peak areas are stated for every elemental map. These values give an indication of the overall variation in quantity for each element. Together with the accumulated spectrum they also show whether an element is present in significant amounts or near background levels.

Line scans for semi-quantitative analyses of the inks were measured on all 24 selected documents. Five adjacent points were measured for five areas per document, one area on the paper and four areas on ink lines. The live time was 10 s for each individual spectrum giving a total of 50 s for the accumulated spectrum of each line scan. Semi-quantification was achieved by calculating the net peak areas normalised to the Compton peak. The Compton peak is a measure for the density of the sample, which directly translates to interaction volume. Similar samples with different interaction volumes usually means that these samples have different thicknesses and a ratio of the peak of interest against the Compton peak is therefore a valid method for semi-quantitative comparisons between similar samples.

Within the project, quantification was also attempted based on comparison with reference samples with known element concentrations. For details about the methods, see instrument reports in (S2 and S3 Files).

## Results and discussion

### Condition survey

**Fig 11:**
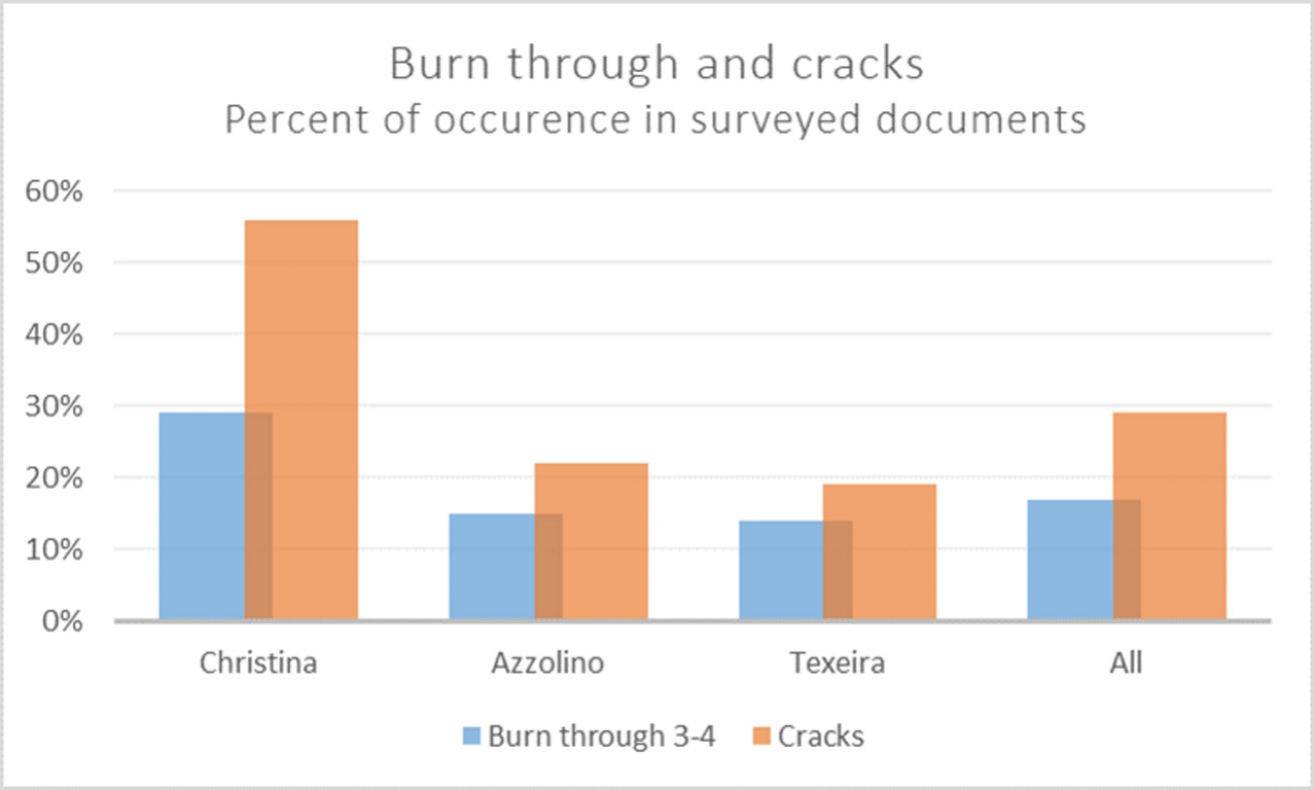
Burn through and cracks in condition survey. Percent of surveyed documents displaying burn through 3–4 and cracks in ink layer, according to writer. Of the 314 documents that were assessed in the survey 55 were written by Christina, 46 by Azzolino, 43 by Teixeira and the rest of the documents by circa 35 other authors.

Important factors influencing the degree of iron gall ink corrosion are, among others, the components of the ink, the thickness of the ink lines, the thickness and sizing of the paper, and the level of relative humidity that the document has been exposed to [29–31]. The corrosion of the Azzolino collection seems to some extent connected to writer, as the documents written by Christina often display severe burn through and cracks (56%) and the documents by Azzolino less so (22%). The documents written by the banker Manuel Teixeira in the Queen’s administration—who is frequently featured in the collection—display less occurrence of cracks (19%) compared with the selected documents in total (29%), see Fig 11. For a full description of the condition survey results, see the (S1 File).

**Fig 12:**
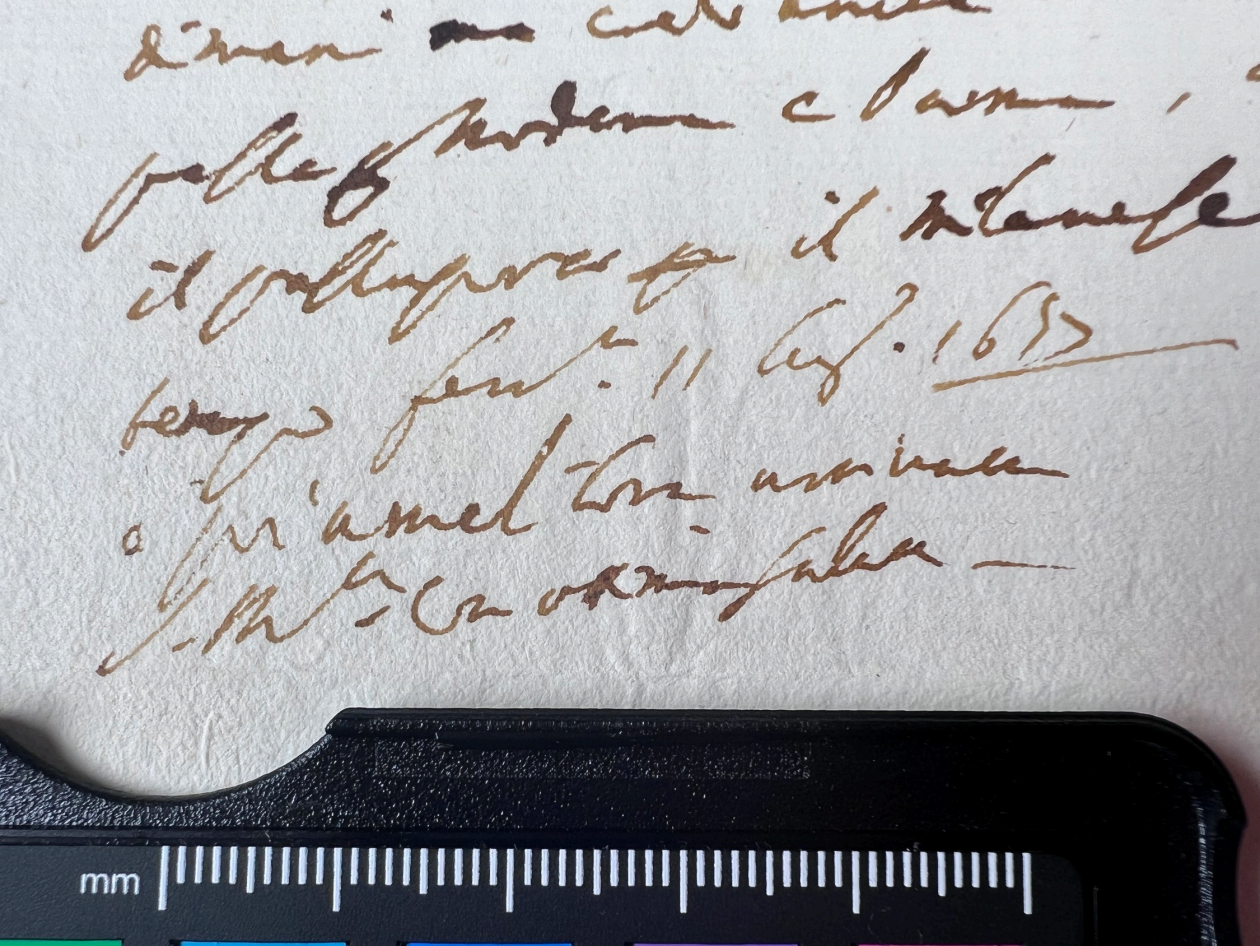
Ink thickness 1. Visual estimation of ink thickness 1, exemplified through a document from volume K 400 of the Azzolino collection.

**Fig 13:**
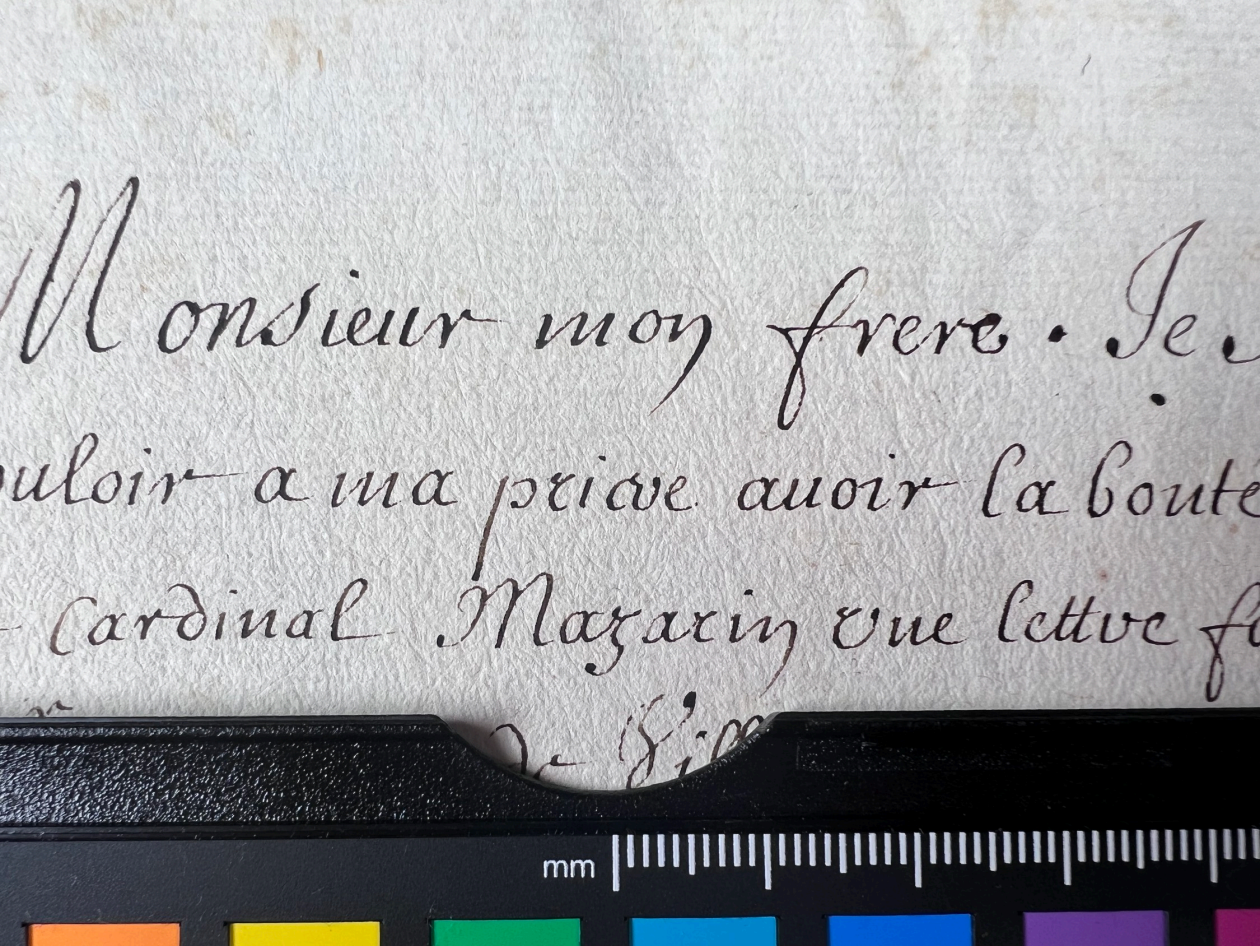
Ink thickness 2. Visual estimation of ink thickness 2, exemplified through a document from volume K 400 of the Azzolino collection.

**Fig 14:**
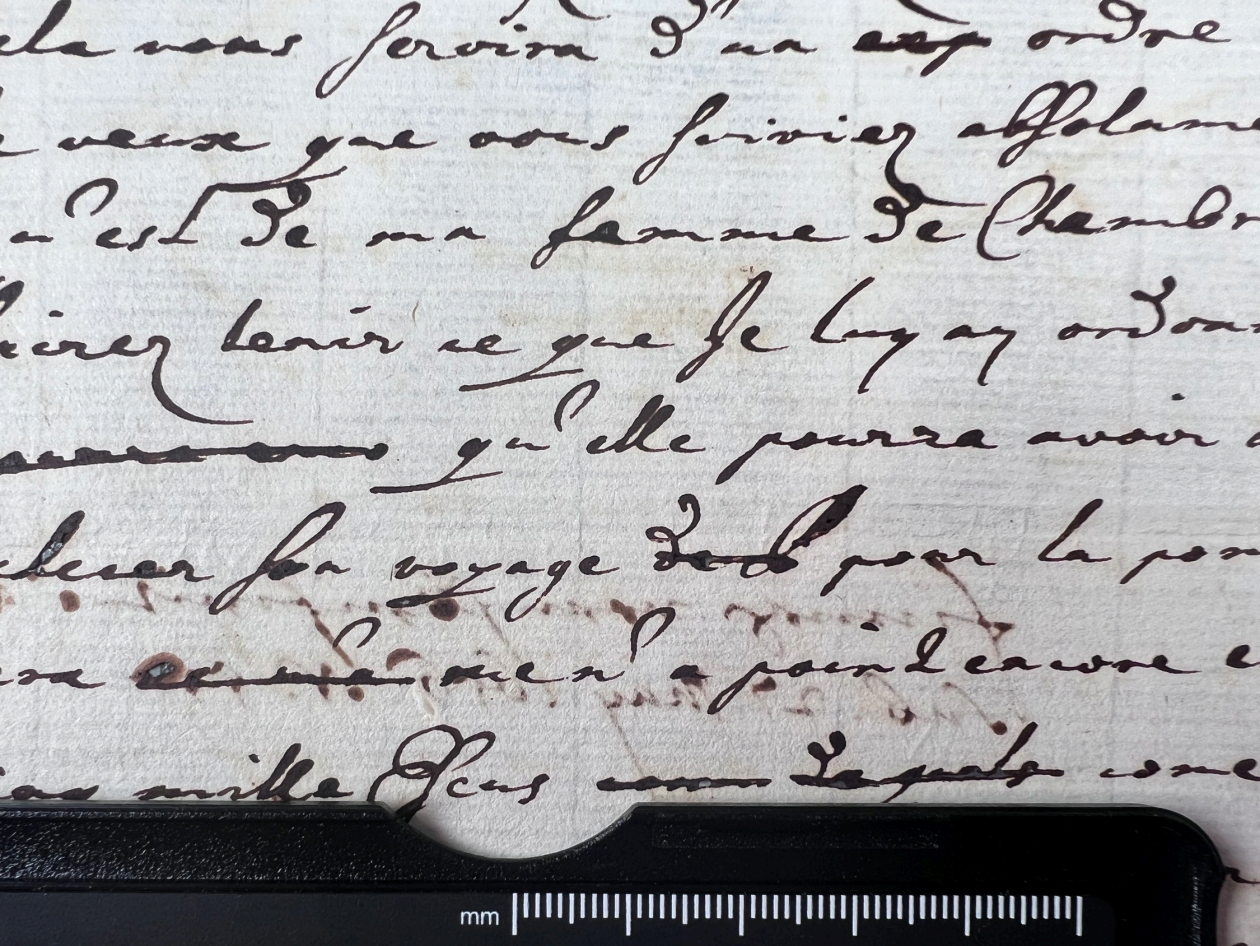
Ink thickness 3. Visual estimation of ink thickness 3, exemplified through a document from volume K 400 of the Azzolino collection.

**Fig 15:**
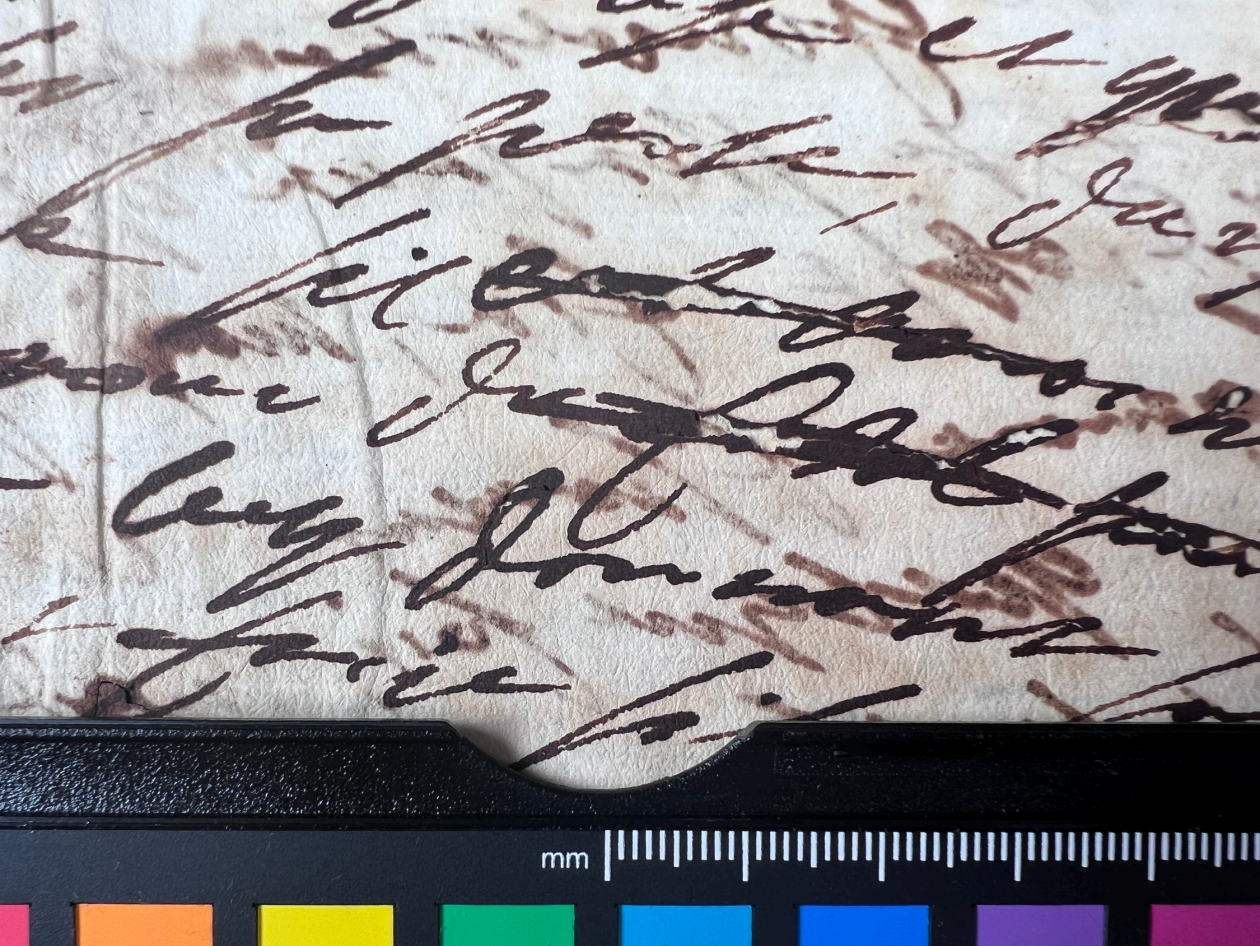
Ink thickness 4. Visual estimation of ink thickness 4, exemplified through a document from volume K 400 of the Azzolino collection.

An assessment of ink line thickness (i.e. z-axis of document) was made by visual examination and expressed as a value from 1 to 4 with 4 being the thickest, see Figs 12–15.

Burn through and presence of cracks were evaluated in relation to ink thickness and observed water damage. In the case of the documents pertaining to the two main authors of the collection, Christina and Azzolino, there are noticeable differences in the results from the survey. The documents written by Azzolino have, to a greater extent, been damaged by water and display a clearer tendency of ink-transfer to adjacent documents, i.e. 42% of Azzolino’s surveyed documents showed water damage but only 24% of Christina’s and for the collection as a whole the number of water damaged documents, as indicated by the survey, is 30%.

**Fig 16:**
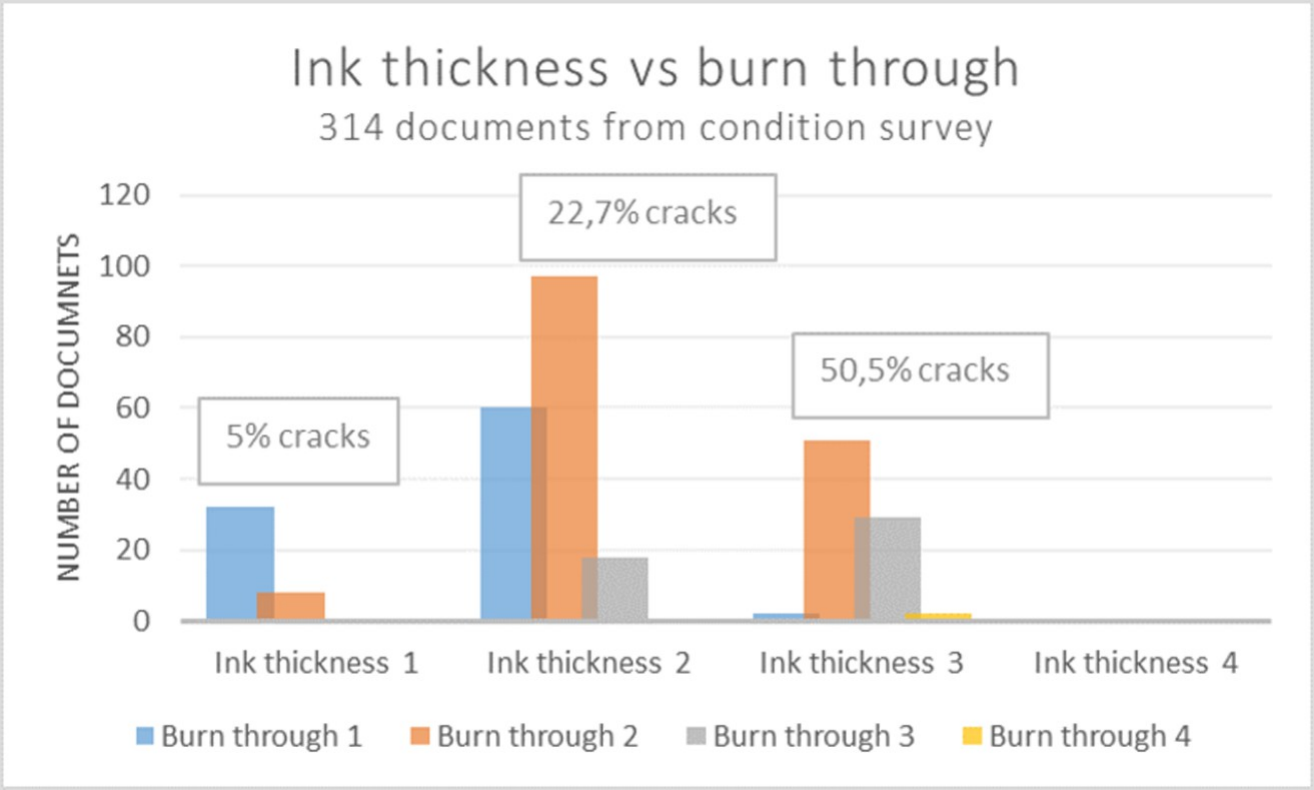
Burn through in relation to ink thickness and occurrence of cracks as assessed by the condition survey. Y-axis displaying number of documents of specified level of overall ink thickness, 1–4, and burn through, 1–4. Inserted boxes present occurrence of cracks within each category of ink thickness.

**Fig 17:**
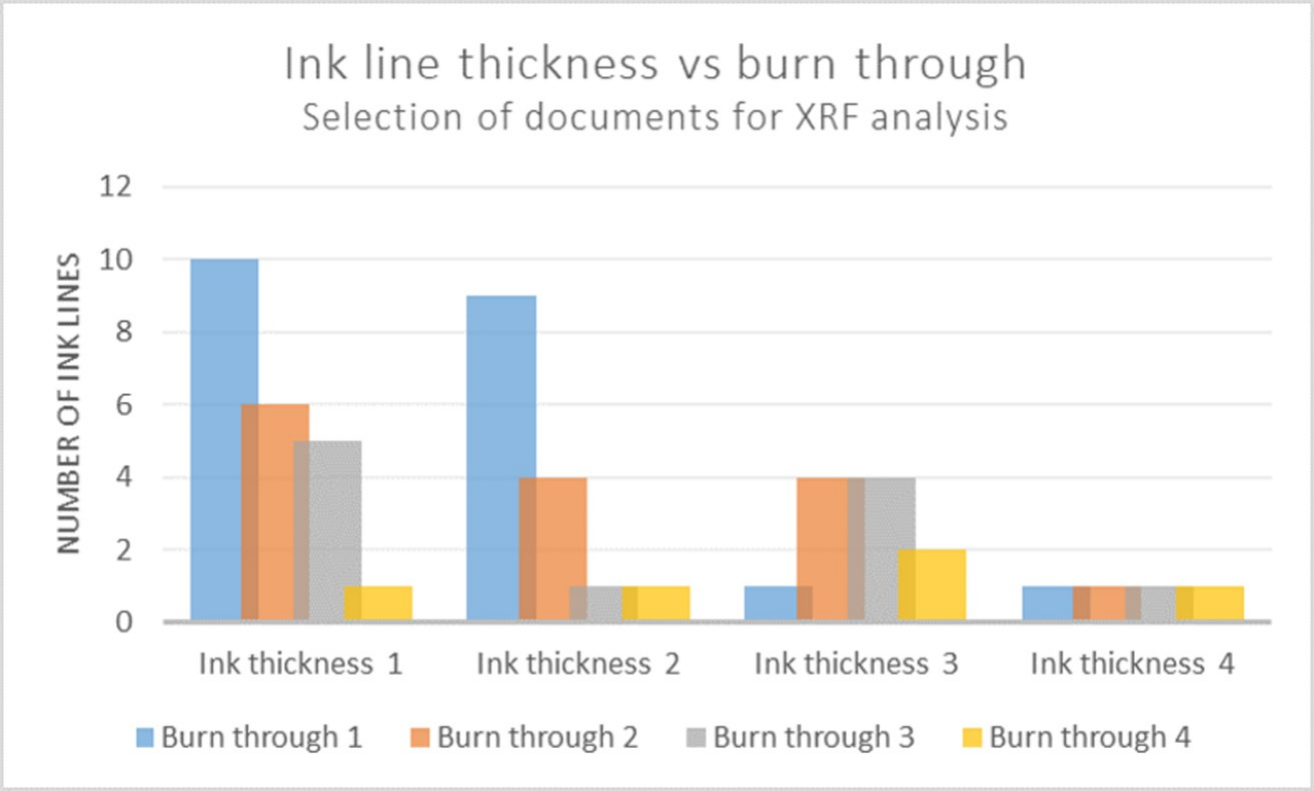
Burn through in relation to ink line thickness of 24 documents for XRF analysis. Y-axis displaying number of ink lines of specified ink thickness, 1–4, and burn through, 1–4.

What seems to be a decisive factor in the development of severe ink corrosion is the thickness of ink lines, as the thicker layer of ink correlates with more aggressive occurrences of ink corrosion in the analysed documents. Fig 16 presents the evaluated ink thickness in relation to burn through and occurrence of cracks of the 314 examined documents from the condition survey. Fig 17 visualizes the condition of the individual ink lines in the 24 documents that were analyzed using XRF. When studying tendencies related to burn through 1, as presented in the blue columns in Fig 16, there are more occurrences of this least severe category of ink corrosion in documents with thin ink lines (ink thickness 1–2), compared with the more advanced corrosion and higher occurrence of cracks of the thicker ink (3). In the condition survey, there were no documents in the statistical selection that displayed an overall ink thickness of 4.

When comparing the ratio of thick ink (3 and 4) to thin ink (1 and 2) from the individual lines of the analysed documents in Fig 17, it becomes clear that the levels of burn through progressively increase as the ink thickness ratio increases: Burn through 1 displaying thick:thin ratio 0.1; burn through 2 ratio 0.5; burn through 3 ratio 0.8; burn through 4 ratio 1.5.

Comparing the thickness of ink between the Christina and Azzolino documents, the latter display a more frequent occurrence of thick lines (ink thickness 3: 41%) than within the Christina documents (ink thickness 3: 27%), but 56% of Christina documents show cracks in the inked areas, whereas only 22% of the Azzolino documents present cracks. Furthermore, 29% of Christina documents have reached category 3 of burn through, while only 15% of the documents by Azzolino are found in category 3 of burn through (see Fig 11). The condition rating categories of burn through 1–4 have been defined and applied according to the description in Table 1, where category 2 signifies a generally fair condition with a possible single occurrence of a crack in an inked line. As Fig 11 demonstrates, there is a noticeable difference between the overall condition rating in category of burn through of the evaluated documents and the total occurrence of cracks. This variation is particularly evident in the documents written by Christina, which *might* indicate a difference in ink composition that makes the ink used by queen Christina more corrosive. Other factors could of course influence the present state, such as paper properties, different storage conditions before Christina’s death and the merger of the collections, or the respective popularity of the documents to visitors and researchers of the Swedish National Archives. As Christina’s documents might more frequently be asked for, they are therefore potentially more exposed to climatic variations and handling.

### XRF characterisation of ink components

The inks of all 24 documents analysed in this study contained predominantly iron, with only relatively small amounts of other inorganic elements present. Common additional elements were copper, zinc, calcium, potassium and manganese. Some instances of nickel, mercury and lead containing inks occurred. Semi-quantification was achieved by calculating the net peak areas normalised to the Compton peak (S2 XRF instrument report).

An attempt to quantify the ink composition indicated mostly very low weight/weight ratios of companion metals (manganese, zinc and copper) to iron. Quantification methods and results are discussed in detail in the (S3 File). Results showed manganese to iron weight/weight ratios of 0–0.02, zinc to iron weight/weight ratios were in the range of 0–0.03 and only one manuscript (K394_0014) had significantly higher levels of zinc with circa 12% (ratio Zn/Fe 0.12). Copper to iron weight/weight ratios were mostly within 0 to 0.02, while three documents showed levels of 10–14% (K394 0014, K409 1608 and K422 3190, all in text written by Queen Christina). This implies that the inks were produced with a refined vitriol (iron (II) sulphate) and even the highest levels of companion metals identified were well below the highest levels reported in previous studies [17–19]. For the assessment of the composition of each ink, measurements were taken from up to five separate areas; see for details (S2 File). The results show some inhomogeneity within the inks, and the detection of companion metals can vary between the analysed areas. Table 2 shows the number of analysed areas of ink for each document and the number of times the companion metals were detected. If an element is present at very low levels, and its characteristic peaks in the XRF spectra are subsequently near the background, then the element may not be detected in each analysed area; while an element that is present at higher levels is more likely to be consistently detected. With this in mind, the results listed in Table 2 indicate a higher occurrence of copper content in the inks used by Christina compared to the inks used by the other writers.

**Table 2:** Presence of elements in analysed documents.

Document	Writer	Location	Date	Condition	Burn through	Cracks	Mn	Cu	Zn	Pb	Hg	Ni
Del Monte Vol II A1 No 17	Christina?	Italy	1687	good	1				2 of 4			
K394 0014	Christina	Germany	1666	fair	1	yes	5 of 5	5 of 5	5 of 5			
K396 266	Christina	Italy		fair	4	yes	2 of 4 (+p)	3 of 4			3 of 4	
K403 828	Christina	Italy	1665	good	2							
K407 1298	Christina	Italy	1677	good	2		1 of 5	1 of 5	1 of 5			
K408 1556	Christina		1685	fair	4	yes	1 of 3	3 of 3	1 of 3			
K409 1608	Christina	Italy	1687	good	1		1 of 4	4 of 4	2 of 4	3 of 4		
K422 3078	Christina	Italy	1669	poor	4	yes		1 of 2				
K422 3190	Christina			good	2			2 of 2	2 of 2			
K429 3767	Christina			fair	1			4 of 4	1 of 4			
K430 3842	Christina			fair	3	yes	3 of 3	3 of 3	1 of 3			1 of 3
K403 828	Azzolino	Italy	1665	good	2							
K415 2098	Azzolino	Italy	1661	fair	1		2 of 4 (+p)	3 of 4 (+p)	2 of 4	3 of 4	3 of 4	
K415 2182	Azzolino	Italy	1671	fair	1		2 of 5 (+p)	2 of 5	1 of 5	1 of 5		1 of 5
K423 3234	Azzolino	Italy	1669	fair	1		3 of 4	1 of 4	1 of 4			2 of 4
K405 1092	Teixeira	Germany	1670	good	1							
K419 2574	Teixeira	Hamburg	1664	good	1		1 of 5		3 of 5			
K420 2868	Teixeira	Hamburg	1689	good	2		2 of 4	1 of 4				
K397 364	Stropp	Sweden	1662	good	3	yes	5 of 5 (+p)		3 of 5			
K399 470	Niclas Marcus	Sweden	1676	good	1		3 of 5	1 of 5	2 of 5			
K401 658	Seved Bååt	Sweden	1659	fair	3	yes	5 of 5	3 of 5	5 of 5			
K412 1902	Adami	Sweden	1665	good	2		4 of 4		4 of 4 (+p)			
K421 3064	Accounts			fair	3	yes						
K408 1556	Secretary		1685	fair	2		3 of 3	1 of 3		2 of 3	2 of 3	
K422 3190	Secretary			good	2		2 of 3	2 of 3	1 of 3			
K429 3800	Astrologer			fair	3	yes	2 of 3					
K429 3801	Astrologer			fair	4	yes						
K422 3078	Secretary	Italy	1669	fair	3	yes			2 of 2	1 of 2	1 of 2	

Number of ink areas where the secondary metals were detected out of total number of areas analysed per document. More saturated colours indicate higher rate of detection for each element. (+p) indicates that the element was also detected in areas of paper without ink.

**Fig 18:**
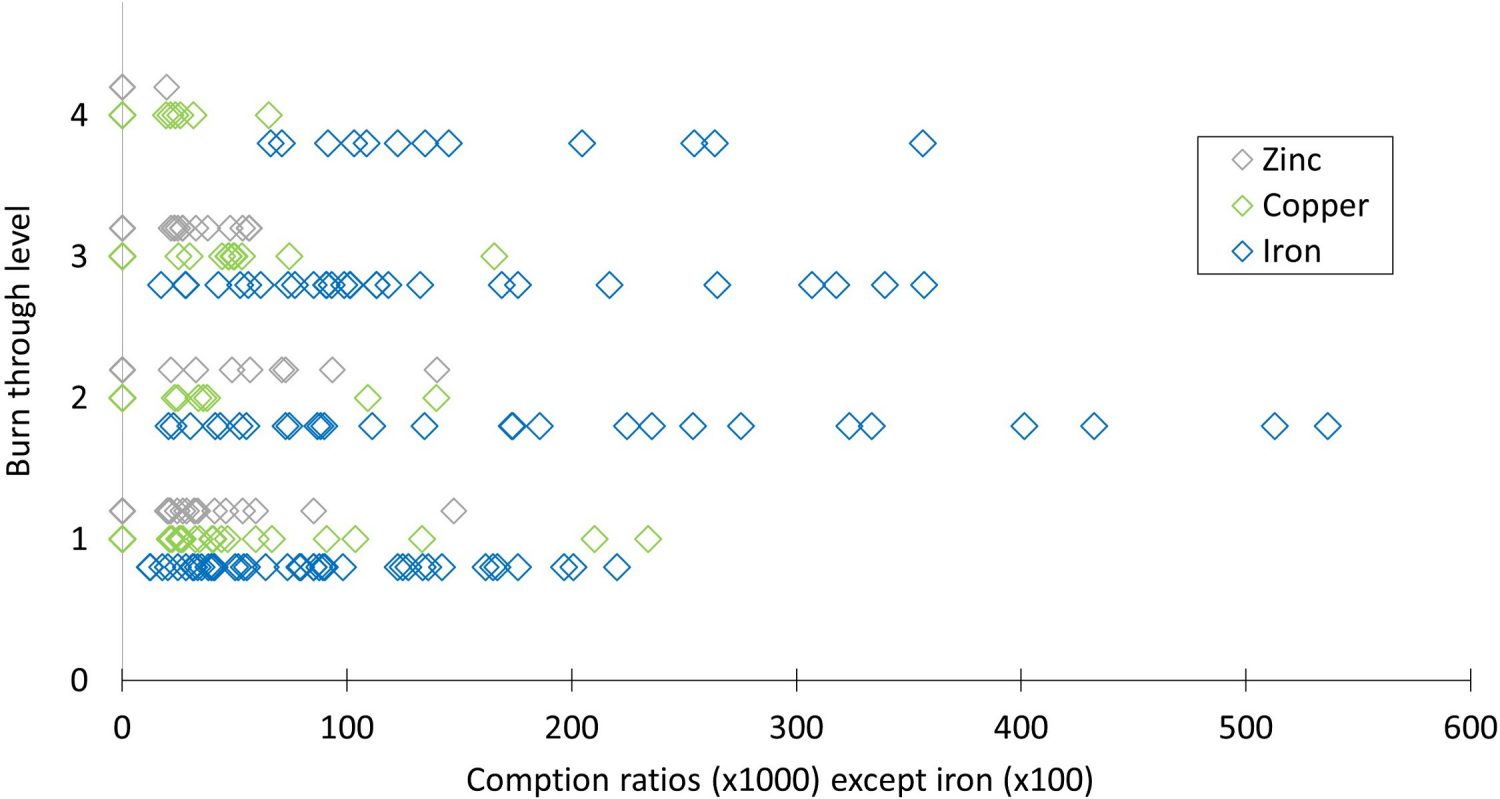
Level of burn through showing no correlation with semi quantitative XRF results. Data expressed as Compton ratios: [net peak area*1000/Compton net peak area] except for iron where [net peak area*100/Compton net peak area] in order to reduce the numbers for iron to improve legibility. All absent peaks were set to 0. In some cases the difference between a peak near background level or an absent peak is marginal and not always obvious. Absent peaks can simply mean present below limit of detection.

Previous studies on iron gall ink suggest that copper could accelerate ink corrosion [12, 13] and it may be possible that this is also the case for Christina’s documents. However, despite some compositional tendencies and comparable Compton ratios of separate ink lines from each document enabling the distinction between different inks, there were no definite correlations between ink composition and ink corrosion, occurrence of cracks or condition of the documents (Table 2 and Fig 18). For example, the three documents with the highest reported levels of copper (K394 0014, K409 1608, K422 3190) did not all show cracks and some were even judged to be in good condition, while the one document written by Queen Christina and judged to be in poor condition displayed only very low levels of copper.

Looking closer at geographical aspects in Table 2, the Swedish letters generally seem to contain less copper and there seems to be more zinc in letters composed north of the Alps than in Rome. Christina’s ink, during her two-year long stay in Hamburg, differs from that used by Teixeira, a more permanent resident of Hamburg. One might argue that the inks used in Northern Europe, here represented by Sweden and Germany, share a compositional tendency in the relatively low presence of copper and a slightly greater occurrence of zinc. However, the levels are not at such a significant level that was common in many Medieval inks of Northern Europe [15, 18].

The limited number of analysed documents using XRF does however entail a significant level of uncertainty when considering these observations as a basis for generalisation with regard to the collection in its entirety.

The document from the Del Monte collection that was included in the study was a letter of questionable origin, signed “La Regina” in 1687, expressing a promise of monetary compensation from the queen to the family of her banker at the time of her death. The Del Monte letter differs in ink composition from the general trend of the inks used by queen Christina in that no copper has been detected in the ink of this document (Table 2). However, in order to confidently draw any conclusions regarding the authenticity of the Del Monte letter based on ink composition, it would be necessary to carry out paleographic analysis in combination with ink analysis on several documents written by Christina during that particular period.

**Fig 19:**
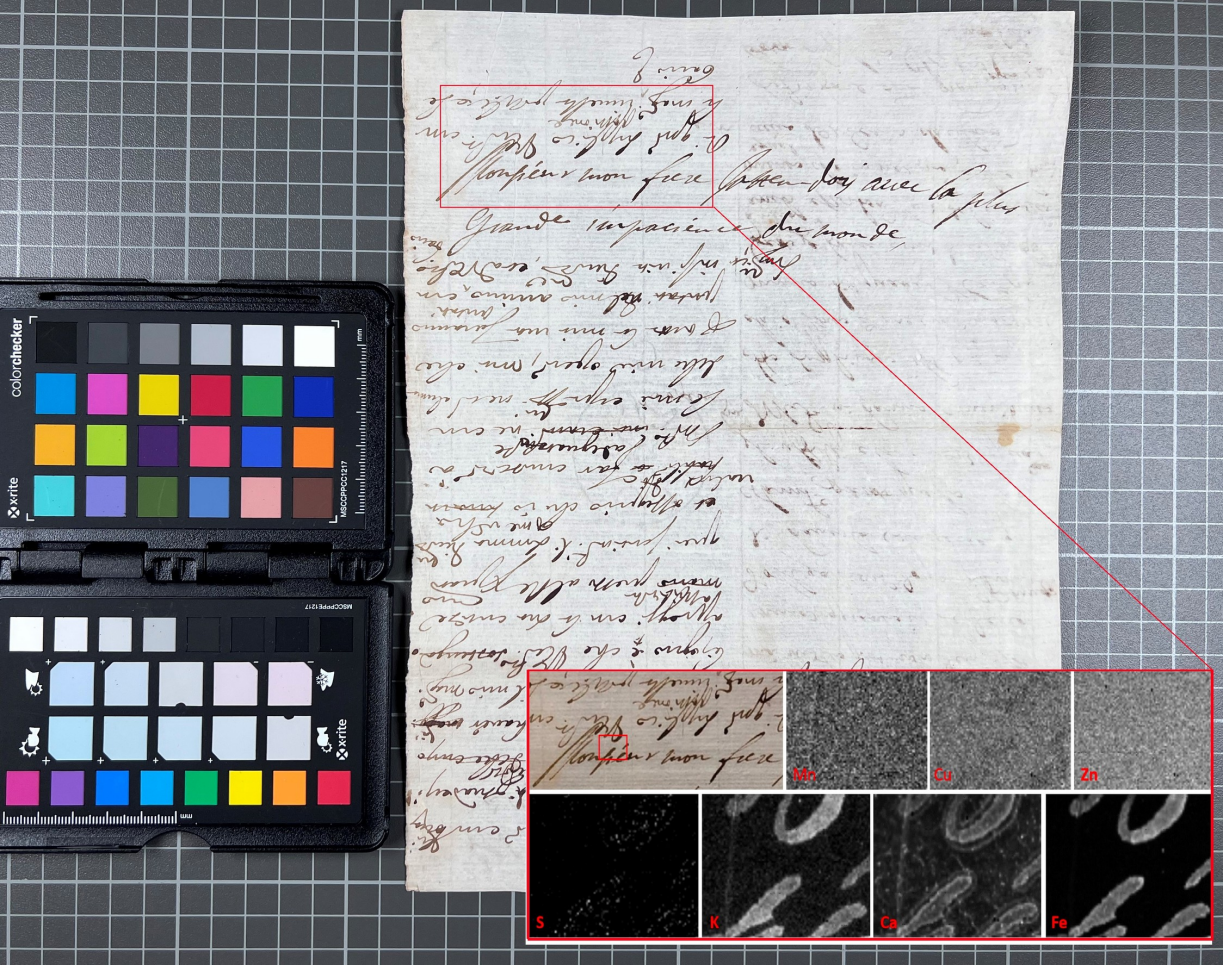
One document, two hands–one ink. XRF map (6532 points) from K403 828 over Azzolino’s (upside down) and Christina’s (right way up) writing. Outliers from dust or other contamination were removed (6 points) to improve contrast in the calcium map. The removed points appear as missing pixels, just noticeable as black dots in the zinc map.

Another letter that differed from the possible trend, in that the ink did not contain copper, is the joint writing by Azzolino and Christina, K403_828 in Table 2. Fig 19 shows a detail of the document written by Decio Azzolino to the French emissary Hughes de Lionne, with a note on the back written by Queen Christina—most likely to the French king on the same matter. XRF mapping of an area containing text by both hands on the same page shows that the inks are of identical composition, suggesting that the two authors have used the same ink, which–judging by its lack of copper–possibly was not one of Queen Christina’s more usual inks. It is an intriguing piece of evidence from a historical point of view, given that the nature of their relationship as well as the issue of Christina’s sexuality has been a topic of discussion in numerous biographical and historical writings [5]. This particular letter does not contain any detailed information regarding such issues, but the results from the analysis do indicate that the two friends probably were close enough to share an inkpot, maybe even facing each other, as their writings are mirrored. This conclusion highlights one of the more important realisations from this project, concerning the narrative possibilities of XRF analysis when combined with historical research.

**Fig 20:**
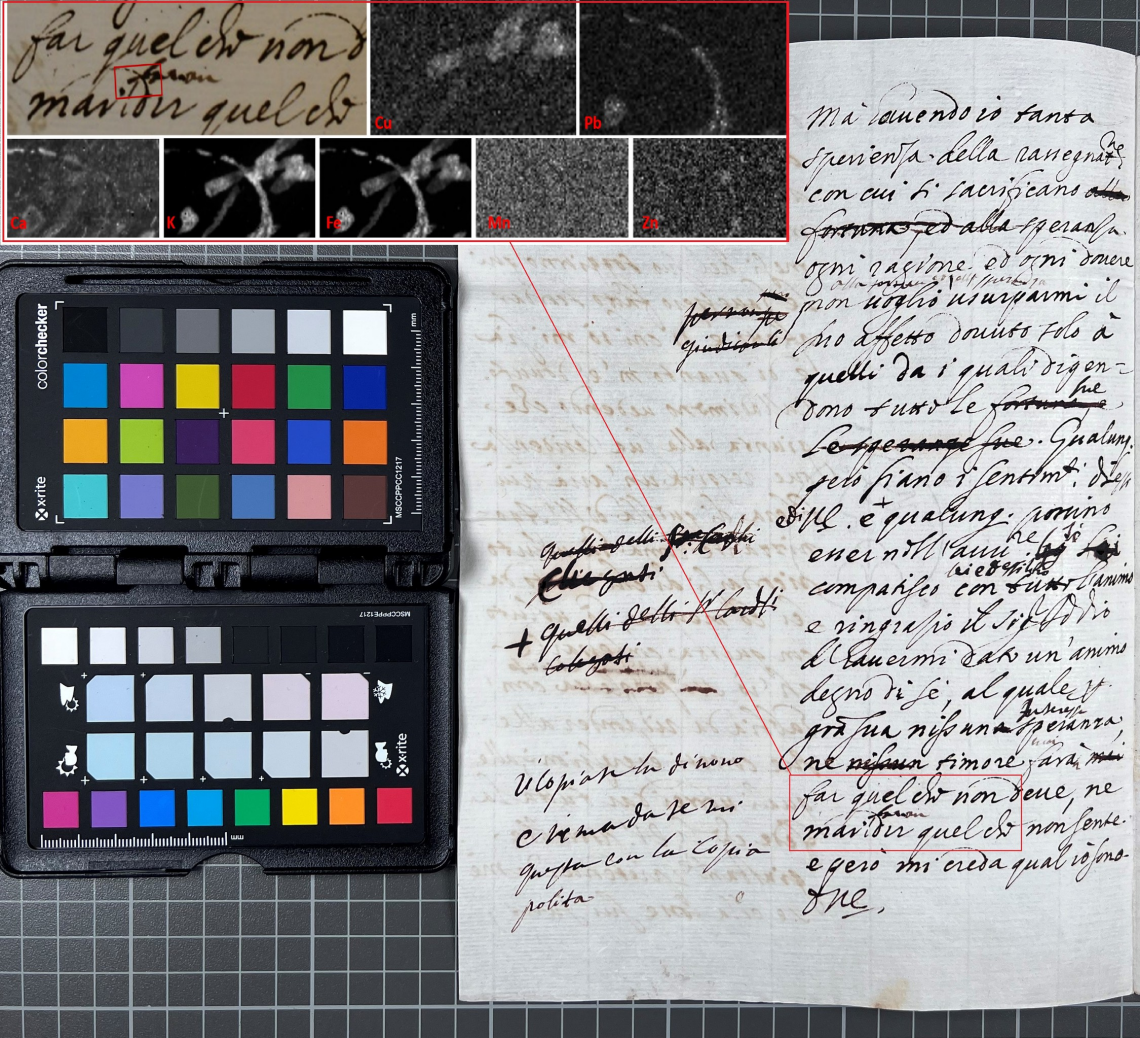
One document, two hands–two inks. XRF map (5600 points) from K408 1556 of area with text written by the Queen’s secretary and notes by herself. Ink used by secretary contains lead but not copper, ink used by Christina contains copper but not lead. Outliers were removed (7 points) to improve contrast in the calcium map.

The XRF map in Fig 20 shows a detail of a document draft written by a secretary to Queen Christina, with inserted notes by Christina herself. The results demonstrate the use of two inks with different elemental compositions–with Christina’s ink containing copper and the secretary’s ink containing lead.

**Fig 21:**
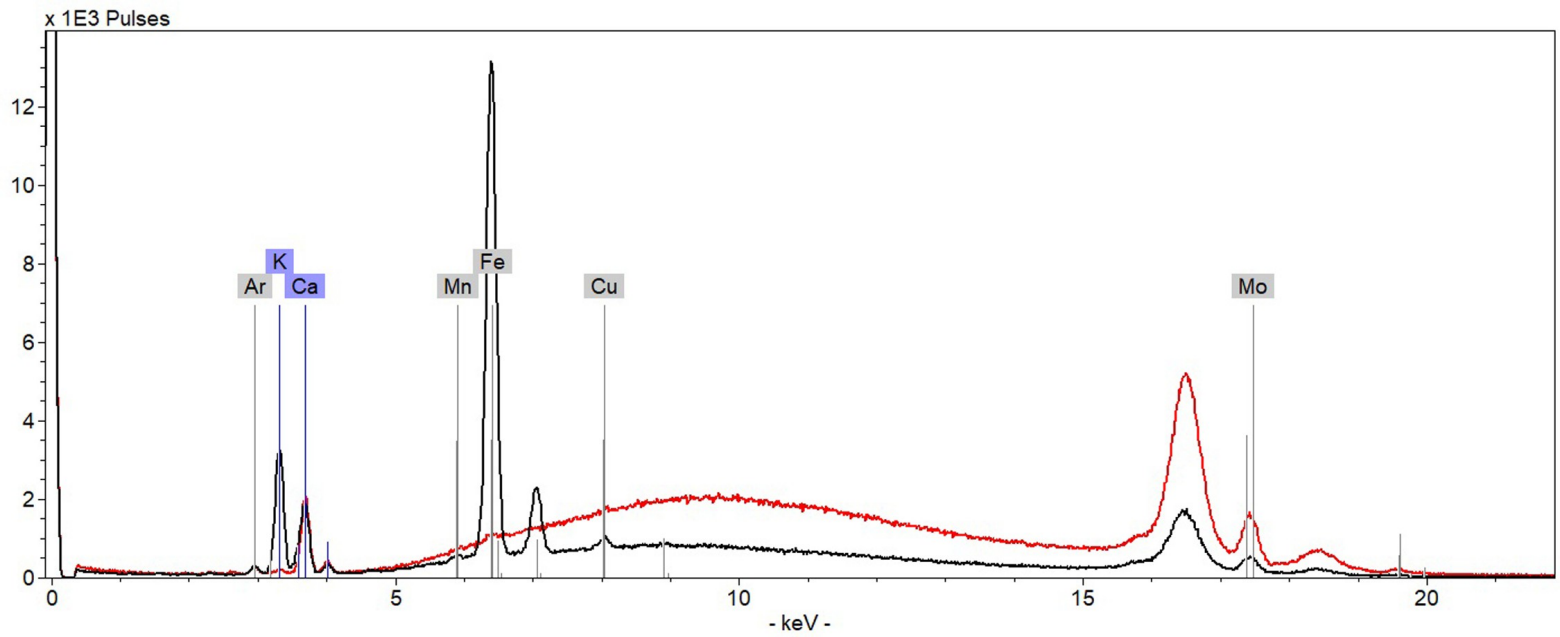
XRF spectra from K408 1556. Accumulated spectra (5 points) of paper (red spectrum) and Christina’s writing ink line 4 (black spectrum).

**Fig 22:**
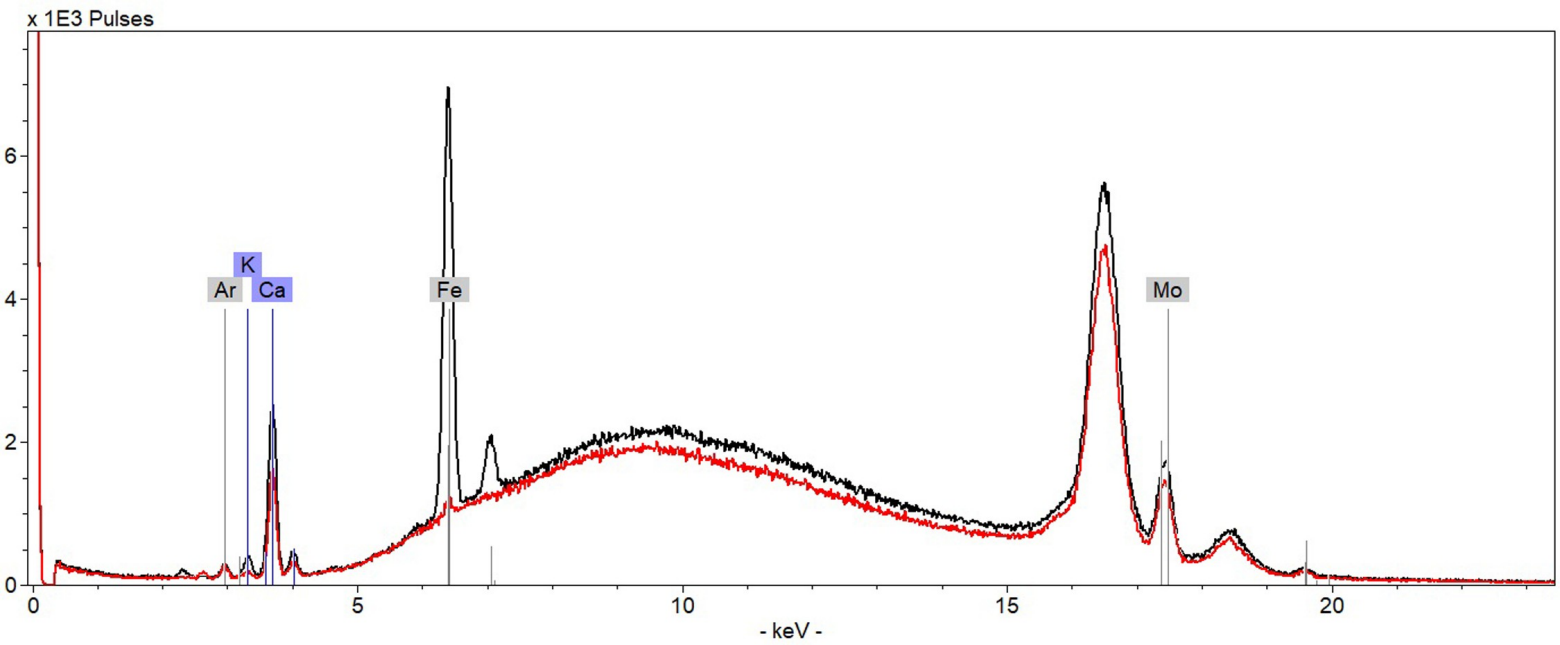
XRF spectra from K403 828. Accumulated spectra (5 points) paper (red spectrum) and Christina’s writing ink line 6 (black spectrum).

All analysed papers showed a significant presence of calcium. Besides the incident molybdenum and the Compton peaks, calcium is the dominant peak for all XRF spectra collected from areas of paper without ink. Spectra collected from ink also show calcium in all cases but the peak is usually lower than that for potassium (Fig 21). Potassium appears to be present in almost all inks. Even in cases where the potassium peak is significantly lower than the calcium peak and appears close to the background level observed in the paper areas, it is clear from mapping results that potassium is associated with the ink (Figs 19 and 22). The presence of potassium, and occasionally calcium, in iron gall inks may be attributed to the gum Arabic binder [32].

**Fig 23:**
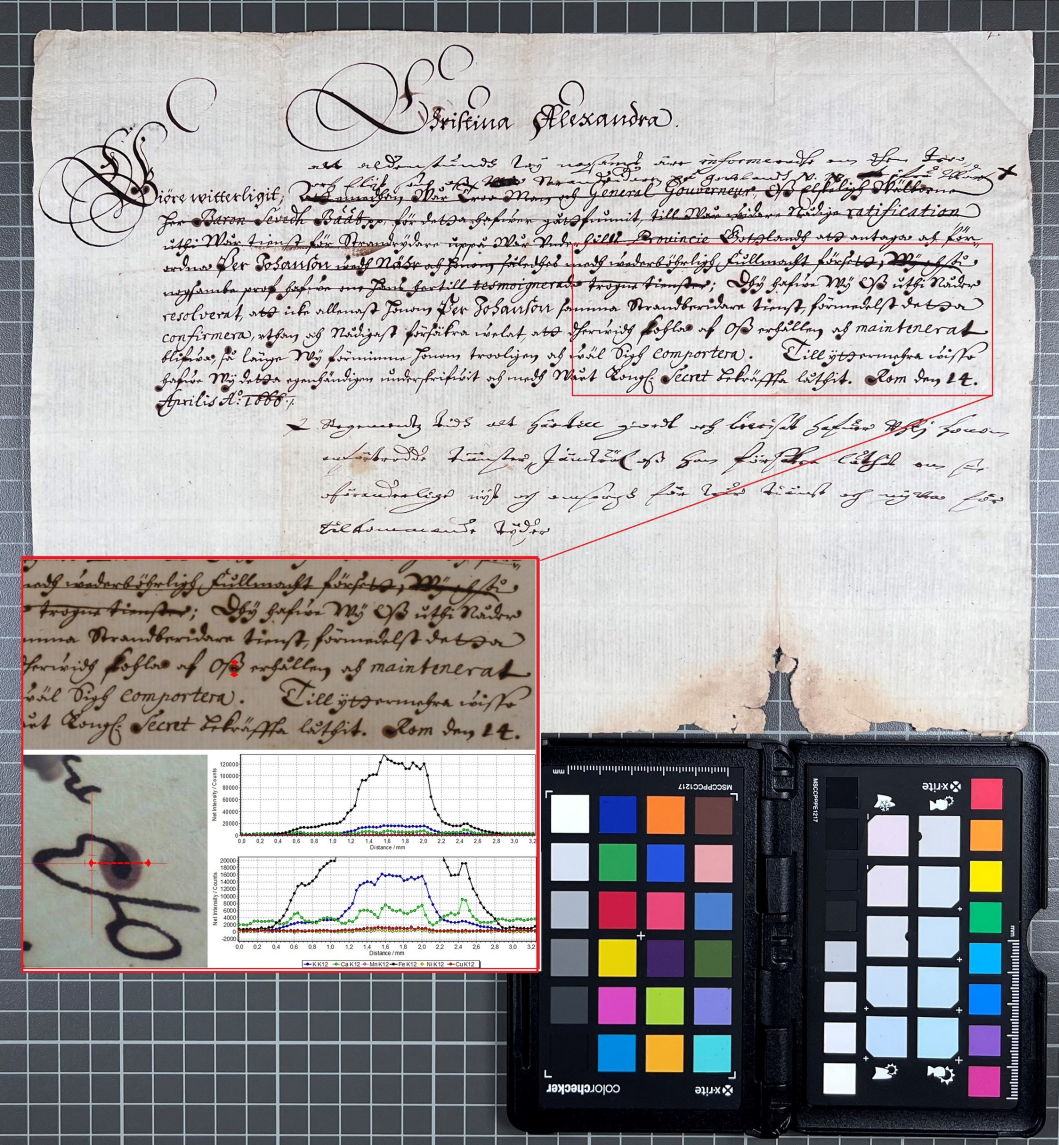
XRF line scan (82 points) from K396 266 over Christina’s ink line with halo, displaying the migration of iron, calcium and potassium from the ink into the paper.

The presence or absence of calcium in the inks is less obvious and could not be determined from comparisons between the spectra collected from the papers with those of the inks. However, the presence of calcium in some inks can be seen in the results of line scans and mapping done across ink lines. Such line scans or maps were carried out for eleven documents (Table 3 and Fig 23). Five of the documents showed a clear association of calcium with the ink. The calcium appears to be more mobile than other elements and partially migrates towards the edges or into the halos of ink lines.

**Table 3:** Association of elements with ink.

Letter	Writer	Presence of deposits or drying materials	Thickness ink layer	Verso, burn- through	Cracks in ink area	XRF Line scan	XRF Map	K	Ca	Mn	Cu	Zn	Pb
K394 0014	Christina	yes	2	1	yes	yes		++	+	+	++	++	-
K396 266	Christina		3	4	yes	yes		++	++	+	+	-	-
K403 828	Christina		2	2			yes	++	++	-	-	-	-
	Azzolino							++	++	-	-	-	-
K407 1298	Christina		3	2		yes		++	-	-	-	-	-
K408 1556	Christina		2	2			yes	++	+	++	++	-	-
	Secretary							++	+	++	-	-	++
K409 1608	Christina		2	1		yes		++	-	-	++	-	-
K422 3078	Christina		3	3	yes		yes	++	+	+	-	-	-
K415 2182	Azzolino		2	2		yes		-	++	-	-	-	-
K412 1902	Adami	yes	2	2		yes		++	++	+	-	+	-
K421 3064	Accounts		3	3	yes	yes		+	++	-	-	-	-
K429 3800	Astrologer		3	3	yes	yes		++	+	-	-	-	

Documents with XRF line scans or maps across inks lines and elements other than iron associated with the inks; ++ indicated clear association of element with ink, + indicated possible association and–indicated no detected association.

The presence of potassium and calcium were evaluated in relation to information gained during the condition survey, such as the presence of deposits, the thickness of the ink lines, the occurrence of cracks and the level of burn through, but no coherent trends were identified (Table 3). However, the influence of potassium and calcium on the condition of the documents would certainly be worthwhile to investigate further, possibly in relation to the presence of deposits on the ink and the development of oxalates, as described by Ferrer and Sistach [33].

## Conclusion

The Swedish National Archive’s Azzolino collection of 4418 documents, dating to the second half of 17^th^ century and written mostly in iron gall ink, was the focus of a condition survey and material investigation. The aims of the study were to understand the variation of conditions of documents to enable a prioritisation for conservation or improved storage, as well as to explore the ink components in relation to writers, geography and ink corrosion. A selection of 314 documents were surveyed, using visual inspection, and a small sub-selection of 24 documents were analysed with XRF. From the information gathered during the initial condition survey, it became evident that Queen Christina’s documents are generally in poorer condition than those of other writers. A clear correlation was observed between the thickness of ink lines and the degree of burn through. No other conclusive correlations could be drawn from the condition survey between the condition of the paper and ink with regards to categorisation according to writer or geography. Generally accepted condition rating categories for iron gall ink documents, linking occurrence of cracks to burn through categories of 3 and 4, were found insufficient in determining the corrosion rate of the ink in the Azzolino collection. Several examined documents, displaying an overall condition corresponding with categories of burn through 2 and even 1, could still present singular occurrences of cracks, predominantly but not exclusively in areas with a thick ink layer. The occurrence of cracks is therefore not clearly correlated with the degree of burn through.

Results from the XRF data indicate that the letters in the selection by Queen Christina more often involved inks containing copper. This may be the cause of the increased tendency of ink corrosion but a correlation could not be established at an individual data point level. Further results indicated that the inks from north of the Alps more commonly contain zinc and present lower levels of copper. Because of the limited number of documents within each category, these tendencies are not strong enough to draw definite conclusions. What is evident, however, is the fact that the content of other metal elements than iron is mostly remarkably low, implying that the vitriol used in the ink production of the latter half of the 17^th^ century was relatively refined.

Using the mapping of elements by XRF, it has been possible to discriminate similarities and differences of inks used on one document. Combining this information with that of content and style provided meaningful information on the context of their creation. Noteworthy has also been that some inks contain calcium and that the calcium has a greater tendency than other ink components to migrate towards the edges and into halos of ink lines.

## Supporting information

S1 File Condition survey. Report on condition survey of the Azzolino collection.
(PDF)

S2 File XRF instrument report. Identification of ink components through XRF analysis of Azzolino documents.
(DOCX)

S3 File XRF instrument report quantitative analysis. Report on quantification method based on XRF analysis.
(DOCX)
