# Supplementary material for: A closer look at the Azzolino collection
Source: PLoS One. 2023 Apr 12;18(4):e0283539. doi: 10.1371/journal.pone.0283539 (PMC10096476; doi:10.1371/journal.pone.0283539)
Supplement: S1 File — Report on condition survey of the Azzolino collection. (PDF) [file pone.0283539.s001.pdf]

Supporting information 3

The Azzolino collection

Iron gall ink corrosion survey

Anna Lagerqvist Alidoost

Thea Winther

2018-06-04/ 2023-02-08

Contents

Screening of the Azzolino collection 3
Sample number 3
On different types of documents 4
Method 5
Results 6
Deposits/Drying material 8
Discussion 10
Possible documents for analysis 11
References 14
Appendix 15
Appendix A Authors confirmed by signature or context in examined documents 15
Appendix B Authors listed in inventory in the entire collection 16
Appendix C1. Screening results of the most frequent authors 17
Appendix C2. Complementary diagrams on screening results 18
Appendix D. Examples of documents with deposits 19

Screening of the Azzolino collection

In 2018, the Unit of Preservation and Conservation at the Swedish National Archives initiated a 3-year project dealing with the various needs of the Azzolino collection, starting with a general condition screening. During the year, in-depth analyses of the material were carried out, following the condition screening and as part of a FoU (Research and development) project in collaboration with the Swedish National Heritage Board. Further on, possible stabilization actions will be investigated and the housing conditions for the collection will be improved. This work would prepare the collection for a future digitization process.

Through minor spot-checks in various parts of the collection, several examples of the iron gall ink corrosion on paper had earlier been observed, albeit with a varying degree of destructive progress. The lack of a systematically established overview constituted a reason for an initial condition screening of the collection, followed by further examination through in-depth analyses on a selection of objects regarding the physical material and its chemical properties.

Sample number

In order to establish an overview of the condition of the collection, and to find interesting objects for in-depth analysis, a statistical selection of documents for evaluation needed to be obtained that would fit the project time frame. To make a statistical selection, a quantitative understanding of the contents of the collection was required, other than the knowledge that there were three shelf meters.

Any accurate information on the number of documents was not possible to find. Based on information from Livrustkammaren (The Royal Armoury), and their digitization project concerning 80 of the Christina letters, that the Azzolino collection consists of approximately 5000 documents (population), this number was considered as indicative of the quantitative extent of the collection. A statistical selection was calculated with a margin of error of 5% and a confidence level of 95% according to:

$$\text{Sample size} = \frac{\dfrac{z^2 \times p(1-p)}{e^2}}{1+\left(\dfrac{z^2 \times p(1-p)}{e^2 N}\right)}$$

where N = Population, e= margin of error and z-score is used to describe confidence, see https://www.surveymonkey.com/mp/sample-size-calculator/ (accessed 2018-09-01)

Entering 5000 into sample size resulted in 357 documents to be investigated.

5000 divided by 357 gave us that every 14th document was to be chosen for inspection providing a random selection of documents.

In reality the collection turned out to contain 4418 documents and 314 were investigated giving us instead a margin of error of 5.4 with a confidence level of 95%. Examples within condition surveying of library and archival materials where methods for a randomized statistical selection are used for

surveys of over 30 items but less than 10% of the population to represent the whole with the given margin of error can be seen in Buchanen and Coleman (1979) and Chrzastowski et al. (1989).

How to decide what constitutes a document is a difficult question and as the screening was carried out on every 14th document, a note on the definition of “document” is necessary. The selection for screening has been made according to its current context and organizational structure, which was created by the turn of the last century. Meaning, the term document can refer to one piece of paper as well as several letters bound together many years ago, a bound book or a letter with inserted pieces of paper with information relevant to the purpose of the letter. The inventory from 1923 has served as a guide, albeit sometimes insufficient, to that end. Printed documents without handwritten comments, in total eight of them in the collection, have been omitted from the screening process as the focus of this project concerns handwritten documents and iron gall ink in particular.

On different types of documents

The vast diversity of the different kinds of documents in the collection is noteworthy as they represent a variety of origins, uses and stages of iron gall ink corrosion. As the collection of queen Christina’s documents was, at the time of her death, joined with those of Cardinal Decio Azzolino and for more than 200 years kept in the Azzolino family archive, the now so-called Azzolino collection contains documents concerning both of these two individuals. The collection contains private as well as official letters and agreements on paper and (some on) parchment, drafts of letters and literary works, protocols, financial accounts, drawings and bound books. The authors are foremost Queen Christina, Cardinal Decio Azzolino and members of the queen’s staff such as Gammal, Santini, Texeira, Brobergen, Bourdelot, Adami, del Monte, Galdenblad; but also prominent individuals such as Charles X Gustav, Louis XIV, the king of Spain, the Duke of Chaulnes, Leopold I, members of the catholic church and several others – see full list in Annex B.

Within the screening process, a number of letters with signatures and known authors were examined but there are also several examples of copies and documents that are unsigned or where the author might be assumed but not confirmed. Out of the 314 examined documents, 55 contain text, comments or signature by Christina (46 written by her alone), 46 letters are from Decio Azzolino and 43 letters from Texeira. Other authors confirmed by signature are listed in Appendix A. Documents for instance labelled as “Miscellanea”, “Mémoire/Memoriale”, “Comptes” and other various documents without known author have also been examined.

The screening process followed the inventory, constructed in 1923 by Ernst Nygren, relying on its description and organization of the collection to the extent possible. However, several volumes displayed an order disconnected from the inventory; some documents seemed to be missing whereas other documents found no resonance in the inventory. The inventory is, furthermore, describing the collection on a general level but specifying details of certain documents, resulting in a fair deal of guess work throughout the screening process, with regard to author, date and general context. The inventory is written in French with additions in Italian, Latin, German and Spanish where the documents related require it. The different types of documents are described as “minute”, “pièce”, “mémoire”/”memoriale”, “lettre”, “autographe”, “livre relié”, “cahier”; indicating the variety of documents within the collection.

Method

314 documents from 57 volumes were selected for evaluation where the condition of the ink and paper was assessed according to a protocol used to define properties, see Table 1. The construction of the protocol was based on properties described in Condition rating for paper objects with iron-gall ink, *ICN-Info # 1*, by Birgit Reissland and Judith Hofenk de Graaff, recommendations from *The Iron Gall Ink Website* and, to some extent, the Library of Congress.[1] Properties assessed for the whole document by visual inspection include the extent of ink coverage; whether or not the ink had been dissolved in water; the extent of ink-transfer from adjacent text; ink-transfer technique; thickness of ink layer in z-axis; extent of iron gall ink corrosion (burn through); presence of drying material, deposits and cracks. The documents were examined in microscope (Olympus SZ4045 stereo microscope 10-20x and Dino-Lite AM7915MZT 10-160x), raking light, UV light 365 nm and transmitted light and the presence of water marks was documented. Photos and microscopic images were taken of examples of different phenomena.

Table 1. Aspects for condition evaluation

	Scale	Yes/no
Large areas covered with ink	1-4	
Dissolved ink in previous water damage		x
Ink-transfer from adjacent texts	1-4	
Ink-transfer technique		x
Thick ink layer	1-4	
Verso, burn through	1-4	
Cracks in ink layer?		x
Drying material present?		x
Deposits on the ink?		x
Appearance in UV? Fluorescence?		X – color specified of fluorescence
Watermark?		x

[1] Reissland, B. & Hoffenk de Graaff (2001), Condition rating for objects with iron-gall ink, *ICN-information nr. 1*, Instituut Collectie Nederland, https://www.scribd.com/document/27703598/Condition-rating-for-paper-objects-with-iron-gall-ink-ICN-info-1, 2018-06-01.
Reissland, B., Scheper, K., Fleischer, S. (2007) Pre-Treatment Assessment – Visual Assessment, *Iron Gall Ink Website*, https://irongallink.org/igi_index5604.html, 2018-06-01.
Albro, S., Biggs, J.L., Dekle, C., et.al. (2008), Developing Guidelines for Iron-Gall Ink Treatment at the Library of Congress, *The Book and Paper Group Annual 27* (2008), p. 129-165., http://cool.conservation-us.org/coolaic/sg/bpg/annual/v27/bp27-20.pdf, 2018-06-01.

Results

Important factors influencing the degree of iron gall ink corrosion are, among others, the components of the ink, the thickness of the ink lines, the thickness and sizing of the paper and the level of relative humidity that the document has been exposed to. Thickness of paper was unfortunately not documented within the screening process. Components of ink will be analyzed in the next step of this project, but a closer look on the thickness of ink lines (expressed in degrees of 1-4, 4 being the thickest) and exposure to water damages in relation to the degree of burn through and presence of cracks might prove beneficial. In the case of the documents pertaining to the two main characters of the collection, Christina and Azzolino, there are noticeable differences in the results from the screening where these properties have been evaluated.

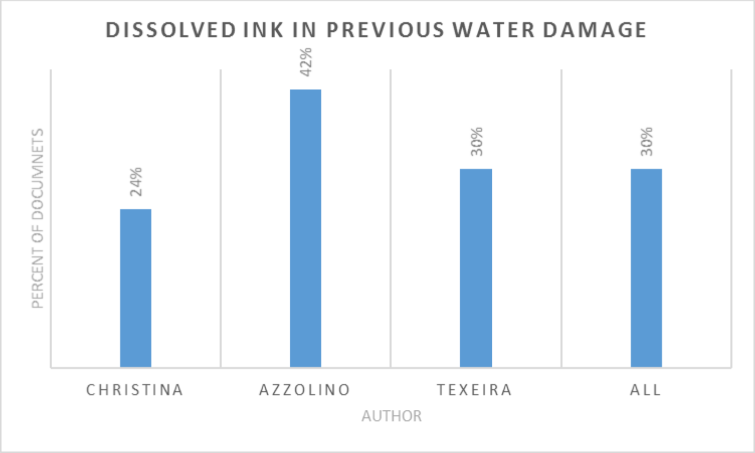

Fig. 1. Percent of documents with ink dissolved in previous water damage.

The documents written by Azzolino have, to a greater extent, been damaged by water and display a clearer tendency of ink-transfer to adjacent documents, see fig. 1 and fig. 14 (in Appendix C2). One reason for this could, however, be that those documents might generally have been kept folded and thereby establishing a direct contact between the ink and other parts of the paper. The occurrence of previously folded versus unfolded objects have not been documented within the screening process.

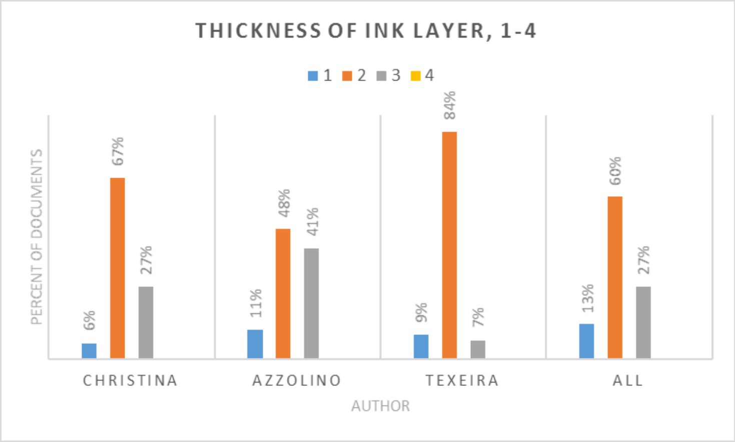

Fig. 2. Thickness of ink layer in the documents, in a scale 1-4, where 4 indicates the greatest thickness.

Comparing the thickness of ink between the Christina and Azzolino documents, the latter display a more frequent occurrence of thick lines (degree 3: 41 %) than within the Christina documents (degree 3: 27 %) but 56 % of these show cracks in the inked areas and 29 % have reached level 3 of burnthrough, whereas only 22 % of the Azzolino documents present cracks and 15 % level 3 of burnthrough, see fig. 2, 3 and 4. This *might* indicate a difference in ink composition that makes the ink used by queen Christina more corrosive. Other factors could of course influence the present state, such as paper properties, different climate conditions before Christina’s death and the merger of the collections or the respective popularity of the documents to visitors and researchers of the Swedish National Archives. As Christina’s

documents might more frequently be asked for, they are therefore more exposed to climatic variations and handling. No statistics of use has, however, been possible to find.

Noteworthy is the fact that the documents written by Texeira display a relatively low occurrence of cracks; 19 % compared with those from Christina (56 %) and the collection in total (29 %). The ink lines of these documents are generally moderately thick (degree 2: 84%), and only 14 % have reached level 3 of burnthrough, but the composition of ink could be interesting to analyze further.

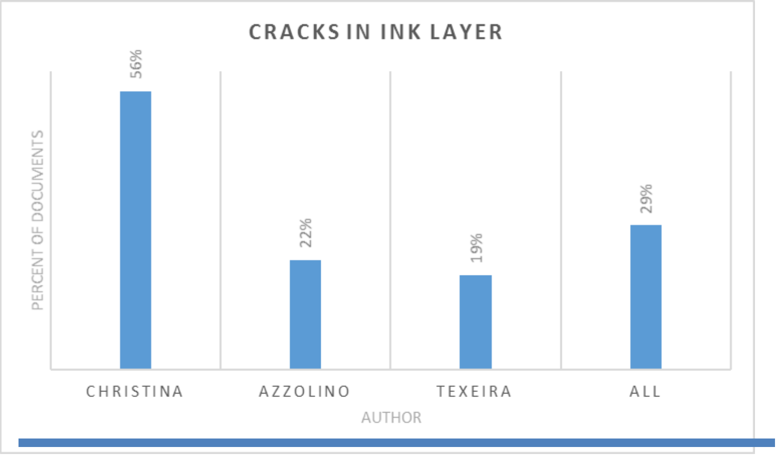

Fig. 3. Percent of documents with cracks in ink layer.

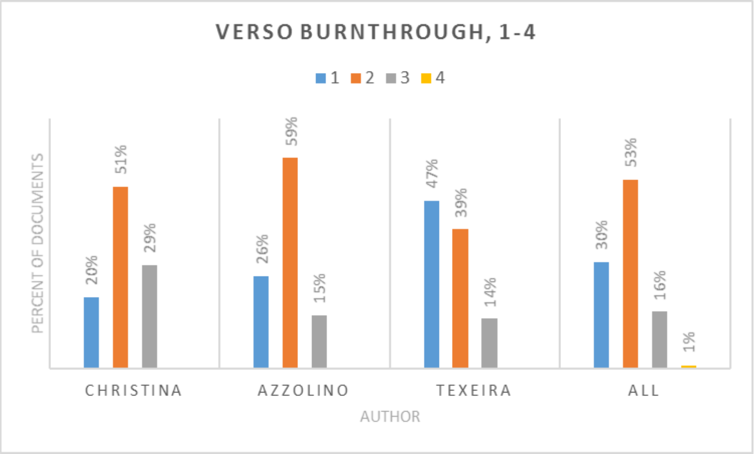

Fig. 4. Level of burnthrough seen from verso of the documents, level 1-4, 4 indicating the greatest thickness.

See full list of screening results and complementing diagrams in Annex C.

Deposits/Drying material

From microscopic images several types could be discerned, grouped together as dark and light deposits, fig. 6 and 7 (table 5 and 6 in Appendix D). Some deposits were identified as drying material in the shape of sand, being black, brown or translucent and irregularly rounded. However, some of these could be iron(III)potassium oxalate and thereby in fact a deposit from the ink instead of an added material by the writer. Deposits that can be described as spiky or needle-shaped and cubic are most likely made of a formation of iron sulphate called amarantite ($Fe(SO_4)\cdot(OH) \times 3H_2O$) according to literature on the subject. White powder or flakes most likely constitute deposits made of calcium sulphate and could possibly be related to the documents having been exposed to water.[2] Unfortunately, the characteristics of each instance of deposits were not specified in the screening protocol, meaning that the properties of the evaluated documents cannot, by the existing data, be correlated to specific types of deposits. The different types of deposits could, however, be investigated further through analysis to gain a deeper understanding of their connection to the composition and condition of specific inks.

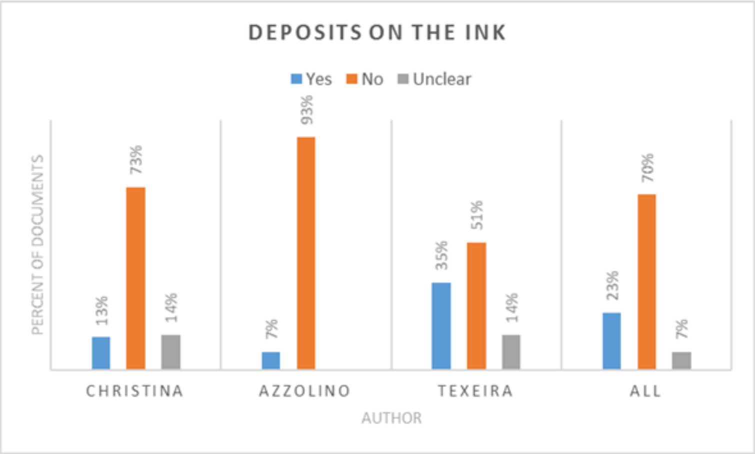

Fig. 5. Percent of documents displaying deposits.

[2] Ferrer, N., Carme Sistach, M. (2013), Analysis of Sediments on Iron Gall Inks in Manuscripts, *Restaurator. International Journal for the Preservation of Library and Archival Material*, Vol. 34, Issue 3, p. 175-193.

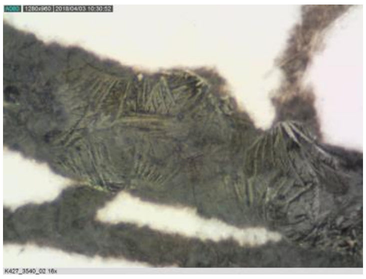

a) Greenish spiky/needle (K427_3540)

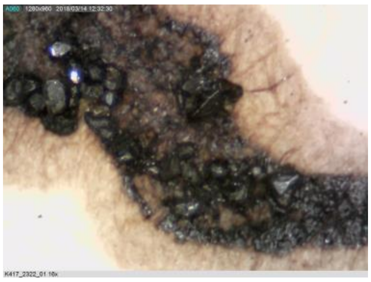

b) Black rounded, shiny (K417_2322)

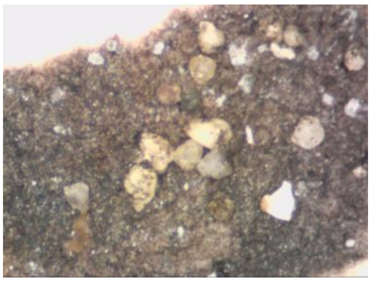

c) Brownish/translucent, rounded (K401_700)

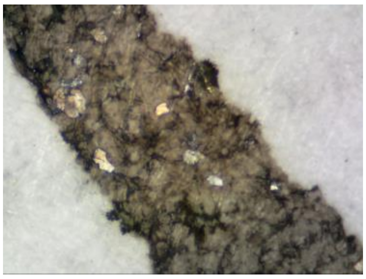

d) Goldish (K401_672)

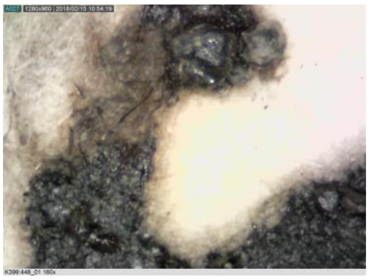

e) Combination black rounded and whitish translucent (K399_448)

Fig. 6. Above: examples of different types of dark deposits (a-e).

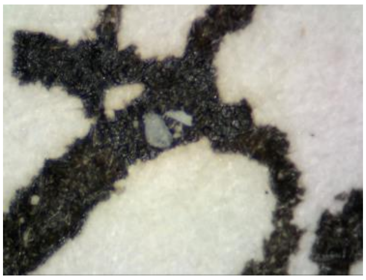

a) Whitish/translucent (K419/420?)

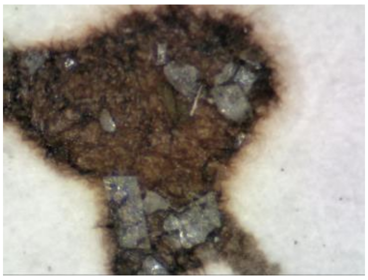

b) Silverish cubic (K419?)

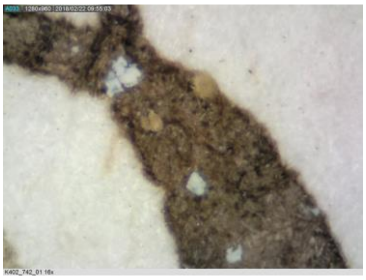

c) White flaky (K402_742)

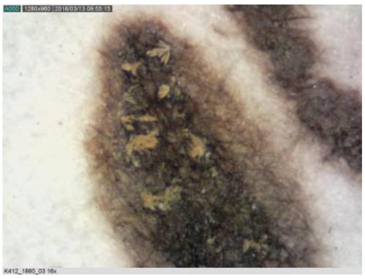

d) Yellowish to white/brown (K412_1860)

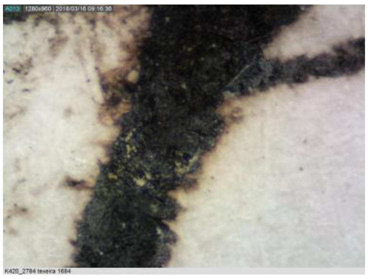

e) White specks (K420_2784)

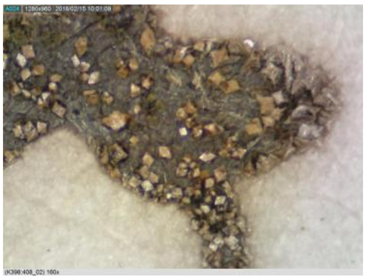

f) Yellow/brown, cubic (K398_408)

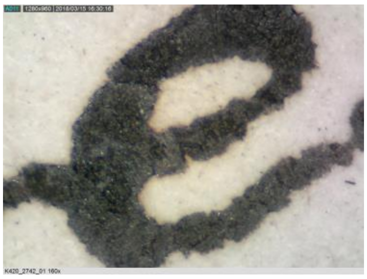

g) Translucent layer (K420_2742)

Fig. 7. Above and to the left: examples of different types of light deposits (a-g).

Discussion

Following the screening process, a meeting has been held with one of the members of the project's reference group, Véronique Rouchon from Muséum National d'Histoire Naturelle (MNHN) in France, and certain points of interest were discussed.

The question if the level of protein content can be related to the sizing of the paper was discussed. The sizing could possibly serve as protection of the paper fibers and thus affect the corrosion process of the iron gall ink. Even though all the documents within the collection are made out of rag paper, the intended function of paper matters as paper made for printing was generally less sized than those intended for writing.

Iron gall ink was used for several centuries and the number of recipes and compositional variations are significant. Certain ingredients are consistently used though, albeit of different origins and proportions; iron (II) sulphate (vitriol), tannic acid (gallotannic or gallic acid), water or wine and gum Arabic. The vitriol is a component that through its inorganic composition is apt for XRF analysis but other elements that are also usually found in iron gall inks are copper, potassium and zink.

For elemental analysis using micro-XRF and XRF mapping, it could be worthwhile to study and compare areas in documents that have and have not been damaged by water, and analyze the content of iron, potassium, zink and other distinguishing elements. Sulphur will probably prove difficult to observe as it is relatively light and has similar energy lines as molybdenum, which the X-ray source that is used by the Swedish National Heritage Board is made of, and might therefore present conflicting results.

Composition of ink could be analyzed in a quantitative way by studying the ratios of copper:iron and zink:iron through the use of micro-XRF. These ratios can point to the type of vitriol that was used in the ink production and serve as a sort of "fingerprint" of the ink, connecting it to a specific geographical area. The content of iron and copper determines the aggression of the iron gall ink corrosion, meaning that the qualitative and quantitative analysis of these components could give an indication of the risk for future degradation and the preservation-related needs of the document. Quantification of ink is however only possible if the ink is in good condition and the paper not too thick.

The presence of deposits is generally not considered to be connected to the degree of iron gall ink corrosion, but might serve as an indication of the elemental composition of the ink and could be interesting to analyze further. Raman analysis could be carried out to that end.

On the screening process

Some categories of observations regarding properties of the iron gall ink and paper are made by visual assessments and based on the collected experiences of the two conservators performing the evaluation. The evaluation has been carried out according to predefined criteria but the assessment of these criteria is, during the screening process, to some extent subjective and the results must therefore not be interpreted as absolute. The categories "Thickness of ink lines", "Large areas covered with ink", "Ink-transfer from adjacent text" should preferably be considered as indicative of the evaluated document's properties in relation to the collection in total.

Possible documents for analysis

Several themes of analysis are suggested:

1) A comparison of ink composition for the ink of the hand of
   a) Christina
   b) Azzolino
   c) Texeira
2) A geographical comparison concentrating on
   a) Rome
   b) Hamburg
   c) Sweden (Gotland and Stockholm)
3) Comparison of areas of water damage to areas of no water damage
4) Possibility of visualizing migration of ink, using both/either multispectral analysis and/or XRF mapping
5) Deposit composition
6) Ink composition based on condition and damage phenomenon
7) Other

Some documents also are candidates for investigating the possibility to increase legibility through visualization. Lastly some suspected forgeries are included to see how the analytical techniques can contribute with information.

Hopefully complementary analysis with several techniques will be possible to carry out for some of the suggested documents but only one suggested technique for respective document is presented in the table below.

Table 4. Possible documents for analysis

Id	Question	Primary suggested analysis	Comment	Category
K398_388	Compare areas of water damage / no water damage	XRF mapping		3
K421_3064	Compare areas of water damage / no water damage	XRF mapping		3, 4
K429_3800/3801	Compare to adjacent document	Multispectral imaging	Corroded document have been subjected to	3, 4

			previous conservation treatment	
K404_952	Ink composition	Micro-XRF	Christina signature, about Gotland, 1666	1a, 2c
K397_371	Ink composition	Micro-XRF	Christina signature, good condition	1a
K434_4058	Ink composition	Micro-XRF	Helsingör, 1658	1a, 2c
K401_601	Ink composition	Micro-XRF	Christina signature	1a
K422_3078	Ink composition	Micro-XRF	Christina autographe, Rome, 1669	1a, 2a
K422_3189	Ink composition	Micro-XRF	Christina/Santini, Rome, 1669	1a, 2a
K394_14	Ink composition	Micro-XRF	Christina, Hamburg, 1666	1a, 2b
K394_42	Ink composition	Micro-XRF	Christina, Hamburg, 1667	1a, 2b
K408_1524	Ink composition	Micro-XRF	Christina, Rome, 1683 (?)	1a, 2a
K409_1608	Ink composition	Micro-XRF	Christina, Rome, 1686	1a, 2a
K407_1298	Ink composition	Micro-XRF	Christina, Rome, 1670s (from inventory)	1a, 2a
K429_3767	Ink composition	Micro-XRF	Christina, alchemy drawing	1a
K397_364	Ink composition	Micro-XRF	Stropp, Stockholm, 1662	2c
K401_658	Ink composition	Micro-XRF	Bååt, Stockholm	2c
K412_1902	Ink composition	Micro-XRF	Adami, Stockholm, 1665	2c
K402_741	Ink composition	Micro-XRF	Azzolino	1b
K403_828	Ink composition	Micro-XRF	Azzolino	1b
K415_2098	Ink composition	Micro-XRF	Azzolino, 1661	1b
K423_3234	Ink composition	Micro-XRF	Azzolino, Rome, 1669	1b, 2a
K415_2182	Ink composition	Micro-XRF	Azzolino, 1672	1b
K419_2574	Ink composition	Micro-XRF	Texeira, Hamburg, 1664	1c, 2b
K405_1092	Ink composition	Micro-XRF	Texeira, Hamburg, 1670	1c, 2b
K420_2868	Ink composition	Micro-XRF	Texeira, Hamburg, 1689	1c, 2b

K422_3190	Migration of ink	Micro-XRF / mapping	Strong warm and cold, yellow, fluorescence	4, 6
K396_266	Sizing analysis	Mapping, Raman?	Defined halo	4, 6
K430_3842	Ink composition	Micro-XRF	Christina, corroded ink, and secretary, less corroded	1a, 4, 6
K394:17	Ink composition	Micro-XRF	Evident cracks in thin lines	6
K398_408	Deposit composition	Raman	A lot of material on the ink, light brown cubic formations	5
K428_3752	Deposit composition	Raman	Text completely covered by greenish deposits, spikes/feather/needles	5
K426_3428	Deposit/drying material composition	Raman	A lot of drying material, also unattached from the paper	5
del Monte collection Montanari – (12/9 1687, Vol.II A:1 nr 17	Ink composition	Micro-XRF	Suspected forgery of Christina	1a
Descartes	Ink composition	Micro-XRF	Suspected forgery of Descartes	7
SDHK218	Investigate possible layers in text	Multispectral imaging	Suspected alteration, text on parchment, 15th century?	4, 7

References

Albro, S., Biggs, J.L., Dekle, C., et.al. (2008)

Developing Guidelines for Iron-Gall Ink Treatment at the Library of Congress, *The Book and Paper Group Annual*, 27 (2008), p. 129-165., http://cool.conservation-us.org/coolaic/sg/bpg/annual/v27/bp27-20.pdf, 2018-06-01.

Ferrer, N., Carme Sistach, M. (2013)

Analysis of Sediments on Iron Gall Inks in Manuscripts, *Restaurator. International Journal for the Preservation of Library and Archival Material*, Vol. 34, Issue 3, p. 175-193.

Reissland, B. & Hoffenk de Graaff (2001)

Condition rating for objects with iron-gall ink, *ICN-information nr. 1*, Instituut Collectie Nederland, https://www.scribd.com/document/27703598/Condition-rating-for-paper-objects-with-iron-gall-ink-ICN-info-1, 2018-06-01.

Reissland, B., Scheper, K., Fleischer, S. (2007)

Pre-Treatment Assessment – Visual Assessment, *Iron Gall Ink Website*, https://irongallink.org/igi_index5604.html, 2018-06-01.

https://www.surveymonkey.com/mp/sample-size-calculator/ (accessed 2018-09-01)

Buchanan, Sarah and Sandra Coleman (1979). *Deterioration Survey of the Stanford University Libraries Green Library Stack Collection.* Materials Science

Chrzastowski, Tina E., David Cobb, Nancy Davis, Jean Geil, and Betsy Kruger, (1989). *Library collection deterioration: a study at the University of Illinois at Urbana-Champaign*. College and Research Libraries 50 (5), p. 577-584.

Appendix

Appendix A Authors confirmed by signature or context in examined documents

**Author**	**Number of documents**
Abbate	1
Lorenzo Adami	7
Angelini	1
Arceiuscouo	1
Decio Azzolino	46
Francesco Azzolino	1
Leonora Baroni	1
Bernardi	1
Bianchi	1
Bourdelot	1
Brancaccio	1
Bustius	1
Bååt	1
Alfonso Carafe	1
Catharina	1
Duc de Chaulnes	3
Ferdinand Chiaraualle	1
Queen Christina	46(54)
Colonna	1
Forberger	1
Galdenblad	1
Gammal	5
Barone de Giengies	1
Macchiati	3
Malaspina	1
Del Monte	5
Angelo Morosini	1
Offerman	1
Orlando	1
Gualdo Priorato	2 (4)
Girolamo Rota	1
Cesare Sacchi	1
Santinelli	4
Santini	1 (4)
Silbercron	1
Stropp	1
Johan Henrik Sylv.	1
Texeira	43
Engelbr. Wilhelm	1
Zetina	3

Appendix B Authors listed in inventory in the entire collection

Christina	D'Yllan	Girolamo Rota	Del Monte
Anna von Brobergen	Seved Bååt	Lionne	Charles XI
Lorentz von Broberg	Bourdelot	MG de la Gardie	Simon Hörman
J. Grottman	Davisson	Orsini	J. Adlercron
Niclas Marcus	Ondedei	Severa	D. Gaspar de Haragza
Offerman	Silbercroon	Leopold I	Panurghi
Jacob Spalding	De Suarez	M. Palbitski	Franc Malavat
Michael Bolts	Louis XIV	Rospigliosi	Olivekrans
Gammal	Le roi d'Espagne	Pontus de la Gardie	Sobieski
G. Gyldenstierna	Cesare Macchiati	Packlin (?)	Duc de Yadagne
G. Olliequist	Lorenzo adami	F.M. Santinelli	Rattisbonne
I. Stropp	D'Archenholtz	Simon Streck	Salomon
N. Forborg	Emporagius	L'abbé Santini	Rapicanti
Karl X Gustav	Marchese Roggi	G. Kurck	Guillaume de Brandebourg
Decio Azzolino	Terlon	Arensburg	Prince d'Orange
P. Appelman	Texeira	Wirtz	Leyonbergh
A. Caraffa	Josua Abensur	Duc de Chaulnes	Banicampi
Chanut	C.G. Wrangel	Rosenbac	Cantersten
Filippo Passarini	G. Bianchi	Bidal	Reichenbach
Tenderini	Cornelius Mysmans	Hedvig Eleonora	Gualdo Priorato
Carlo Conti	Marquis d'Astorga	Abbé de Saint Martin	Arciuscouo di Firenze
Giuseppe Malaspina	Clairet Poissonet	De Buy	Pedro de Mediani
P.F. Pezza	Manoel Valensin	Michel de Pologne	Andreas eps Cracoviensis
Marie Aldobrandini Ceri	AB. Bardt	Jean Stephani Wydzga	Ranuzzi
Zetina	Bernardo Bernardi	Archivescoouo di Corinthe	Angelo abate Farratini
A. Totten	Giov. Battista Patemi	Francesco Azzolino	Billiote
G. Lillieflycht	Man. Colonna	Giuseppi Saluoni	Jean Simonna
J. Vidikindi	Bremond	Ferdinando de Chiaraualle	Rinaldo Santoloni
Nuncius Apostolicus	Ferd. Orlandi/o	La princesse de Vrsins (?)	Samuel Porberger
Mattias del Monte	Rosenbach	Joan. Teod. Reynal	Longueuil
Christoffer de Forberger	Bobixensky	Beyna	Gabriel Carlo Ferri Arp (?)
Bildt	Piru Ligorio	Landini	Joh. Henr. Sylv.
Hieronymus Castellus Phys	P. Ant. Viegra	Estienne des Rousseau	Gio. Francesco Tudino Perito
Jean Peaul, comte de Cerdan	Teresa della Valle	Marie de Vie (uilb?)	Franciscus Spoleti
Francois de St: Bernard	P(F)rancois Nuñez Sanchez	Scopatore	Franc. Cechius
Pier de Rossi	Bengt Oxenstierna	Angelo Morosini	Gio. Ant. Ribotti
Dominique Marie Lassatti	Pierre Anger	Puffendorff	La Chancellerie de Christine
Gio Batta RizziCardinal Pro-Dataire	Lorenzo Pillati	Caspar Sabanelli	

Appendix C1. Screening results of the most frequent authors

	Christina (55)	Azzolino (46)	Texeira (43)	All (314)
Large areas covered with ink, 1-4	1: 27 %<br>2: 27 %<br>3: 33 %<br>4: 13 %	1: 4 %<br>2: 54 %<br>3: 21 %<br>4: 21 %	1: 51 %<br>2: 40 %<br>3: 9 %<br>4:	1: 34 %<br>2:39 %<br>3: 19 %<br>4:6 %<br>Undocumented: 2 %
Dissolved ink in previous water damage	Yes: 24 %<br>No: 76 %	Yes: 42 %<br>No: 58 %	Yes: 30 %<br>No: 70 %	Yes: 30 %<br>No: 70 %
Ink-transfer from adjacent texts	1: 80,0 %<br>2: 18 %<br>3: 2 %<br>4:	1: 50 %<br>2: 43 %<br>3: 7 %<br>4:	1: 86,0 %<br>2: 14,0 %<br>3:<br>4:	1: 69 %<br>2:27 %<br>3: 4 %<br>4:
Ink-transfer technique	Yes:<br>No: 100 %<br>Unclear:	Yes:<br>No: 95, 7 %<br>Unclear: 4,3 %	Yes:<br>No: 100 %<br>Unclear:	Yes:<br>No: 97,7 %<br>Unclear: 2,3 %
Thick ink layer, 1-4	1: 6 %<br>2: 67 %<br>3: 27 %<br>4:	1: 11 %<br>2: 48 %<br>3: 41 %<br>4:	1: 9 %<br>2: 84 %<br>3: 7,0 %<br>4:	1: 13 %<br>2: 60 %<br>3: 27 %<br>4:
Verso, burnthrough, 1-4	1: 20,0 %<br>2: 51 %<br>3: 29 %<br>4:	1: 26 %<br>2: 59 %<br>3: 15 %<br>4:	1: 47 %<br>2: 39 %<br>3: 14,0 %<br>4:	1: 30 %<br>2: 53 %<br>3: 16 %<br>4: 1 %
Cracks in ink layer?	Yes: 56 %<br>No: 44 %	Yes: 22 %<br>No: 78 %	Yes: 19 %<br>No: 81 %	Yes: 29 %<br>No: 71 %
Drying material present?	Yes: 9 %<br>No: 80 %<br>Unclear: 11 %	Yes: 9 %<br>No: 87,0 %<br>Unclear: 4 %	Yes: 12 %<br>No: 81 %<br>Unclear: 7,0 %	Yes: 13 %<br>No: 82 %<br>Unclear: 5 %
Deposits on the ink?	Yes: 13 %<br>No: 73 %<br>Unclear: 14 %	Yes: 7 %<br>No: 93 %<br>Unclear:	Yes: 35 %<br>No: 51 %<br>Unclear: 14 %	Yes: 23 %<br>No: 70 %<br>Unclear: 7,0 %
Appearance in UV? Fluorescence?	None: 24 %<br>Warm Y (Y): 44 %<br>Cold Y (G): 65 %	None: 22 %<br>Warm Y (Y): 70 %<br>Cold Y (Y): 70 %	None: 35 %<br>Warm Y (Y): 46 %<br>Cold Y (G): 49 %	None: 24 %<br>Warm Y (Y): 58 %<br>Cold Y (G): 61 %
Watermark?	Yes: 78 %<br>No: 22 %	Yes: 67 %<br>No: 33 %	Yes: 75 %<br>No: 23 %<br>Undocumented: 2 %	Yes: 80 %<br>No: 20 %

Appendix C2. Complementary diagrams on screening results

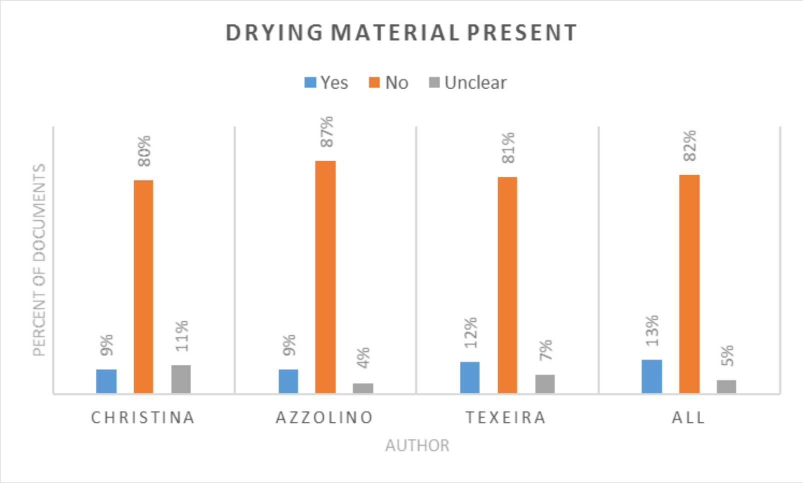

Fig. 10. Percent of documents with drying material present.

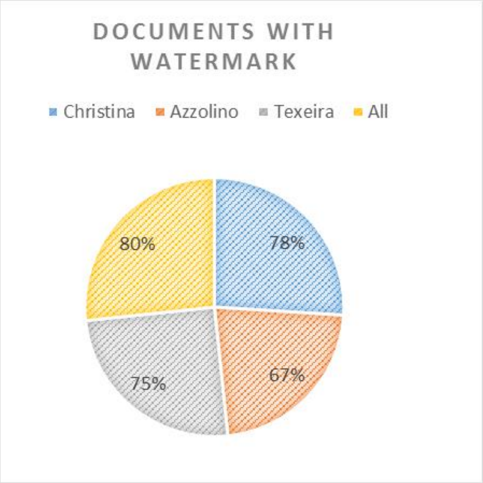

Fig. 11. Percent of documents with watermark.

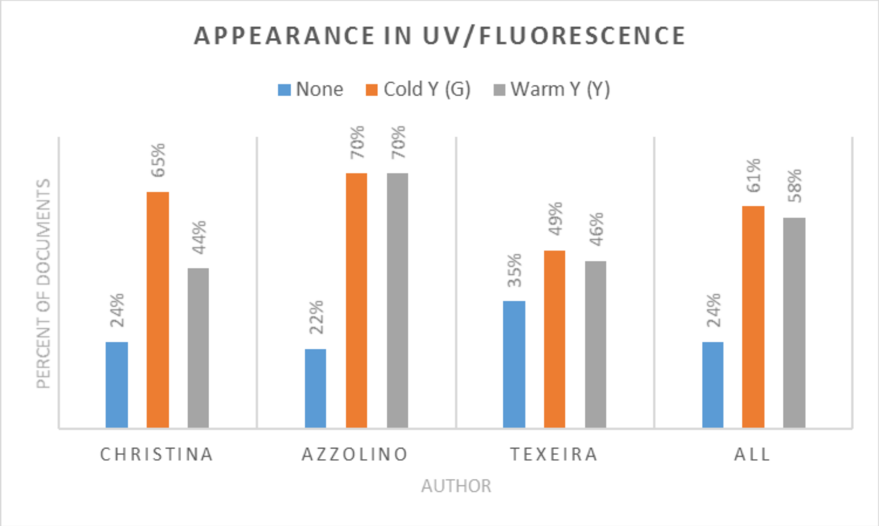

Fig. 12. Percent of documents with different kinds of appearance in UV (none/warm/cold).

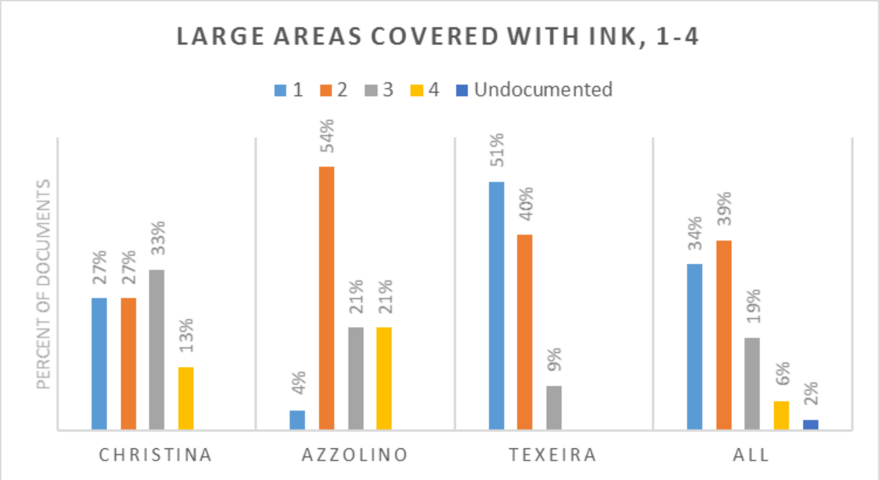

Fig. 13. Percent of documents with various degrees of ink coverage.

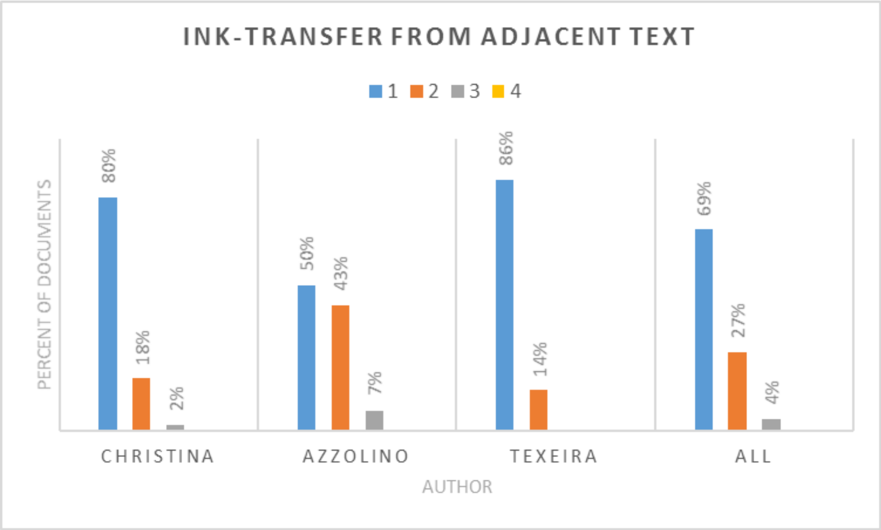

Fig. 14. Percent of documents with various degrees of ink-transfer from adjacent text.

Appendix D. Examples of documents with deposits

Table 5. Documents with dark deposits

Description of deposit	*Greenish spiky, needle*	*Black, rounded shiny*	*Brownish, translucent Rounded*	*Goldish*	*Combination black rounded and whitish translucent*
Name of document	?<br>K427_3540<br>K428_3752 (w.yellow)<br>K401_602 (dotted)	K394_42<br>K394_14<br>K406_1188<br>K417_2292<br>K417_2308<br>K417_2322<br>K418_2434	K394_56<br>K417_2294<br>K395_196 or sand?	K401_672	K403_854

Table 6. Documents with light colored deposits

Description of deposit	*Whitish Trans-lucent*	*Silverish cubic*	*White flaky*	*Yellowish to White /brown*	*White specks*	*Yellow /brown, cubic*	*Trans-lucent layer*
Name of document	K395_224<br>K399_448<br>K401_700<br>K402_770<br>K410_692<br>K419_2700	?<br>?	K395_238<br>K402_742<br>K402_770	K398_408	?	K412_1860<br>K412_1902<br>K415_2070<br>K420_2784	K412_1888<br>K420_2826<br>K420_2742<br>K420_2770
	**Sand?**	**K401_700**					
