# Supplementary material for: A closer look at the Azzolino collection
Source: PLoS One. 2023 Apr 12;18(4):e0283539. doi: 10.1371/journal.pone.0283539 (PMC10096476; doi:10.1371/journal.pone.0283539)
Supplement: S2 File — Identification of ink components through XRF analysis of Azzolino documents. (DOCX) [file pone.0283539.s002.docx]

**Datum** 2018-10-25

**Dnr** 3.5.1-04051-2017

**Fyndnr.**

**Löpnr:**

**Handläggare**: Tom Sandström

**Författare**: Marei Hacke

**Riksantikvarieämbetet**

Artillerigatan 33
Box 1114
621 22 Visby

**Tel** 08-5191 8000

**E-post** riksant@raa.se

**Hemsida** www.raa.se

**Org.nr** 202100-1090

**Plusgiro** 59994-4

**Bankgiro** 5052-3620

# µ- XRF Instrument Report

**Date of analysis** 2018-06-11 – 2018-06-15

**Analyst** Tom Sandström and Marei Hacke

**Table of contents**

µ- XRF Instrument Report 1

Samples 2

Purpose 2

Method 2

Instrument parameters 3

Notes for interpretation 3

Results (spectra, line scans and maps) 6

Del Monte, Montanari – (12/9 1687), Vol. II, A:1 nr 17, Christina (?), good condition 6

K394_0014, fair condition, Christina, Hamburg, 1666 12

K396_266, Rome, April 1666, fair condition 21

K397_364, Stropp, 1662, Stockholm, good condition 30

K399_470, Niclas Marcus, 31 March 1676, Visby, good condition 37

K401_658, Seved Bååt, 19 Jan 1659, Stockholm, fair condition 44

K403_828, page 1: Azzolino and page 2: Christina (?), good condition, 1665 51

K405_1092, Texeira, June 1670, Hamburg, good condition 61

K407_1298, good condition, Christina, Rome, 1677 68

K408_1556 page 2, fair condition, Christina (?) and secretary (?), 1685 76

K409_1608, good condition, Christina, Rome, 1687 87

K412_1902, Adami Aug 1665, Stockholm, good condition 94

K415_2098, fair condition, Azzolino, 1661 101

K415_2182 fair condition, Azzolino, 1671 107

K419_2574, Texeira, 6 Aug 1664, Hamburg, good condition 115

K420_2868, Texeira June 1689, Hamburg, good condition, deposits present 122

K421_3064, “Comptes”, water damage, fair condition 128

K422_3078, fair condition, Christina signature, Rome, 1669 138

K422_3190, Mémoire Christina and secretary, strong fluorescence, good condition 146

K423_3234, fair condition, Azzolino, Rome, 1669 158

K429_3767, fair condition, Christina, Alchemy drawing 164

K429_3800, Christina (?), fair condition 171

K429_3801, Christina (?), fair condition 180

K430_3842 Part 1 fair condition, Christina and secretary 187

Results table 195

Results graphs 198

# Samples

**RAÄ Dnr 3.5.1-04051-2017**

**Riksarkivet Dnr 2017/7960**

All object numbers and associated report page numbers are listed in the table of contents above.

**Description of samples** letters from the Azzolino collection

**Age** second half 17^th^ century

**Material** paper with iron gall ink

**Concentrations** N/A

**Point of analysis** see images in Results section

# Purpose

XRF analyses are carried out to investigate the inorganic components of iron gall inks used in the Azzolino collection with regards to potential variations between documents written at different times, by different authors, in different places and which show a breadth of degradation indicators such as burn through, halos and cracking.

# Method

**Sample preparation** letters were analysed in-situ at Riksarkivet in Marieberg, Stockholm. The single sheets were supported on a PMMA sheet, which gave a blank background of Bremsstrahlung.

XRF spectrum of background sheet, poly(methyl methacrylate), brand name “sabic-ip”

## Instrument parameters

**x**  µ-XRF Artax 800, Mo X-ray tube with polycapillary lens, Bruker; Berlin, Germany

  single point analysis (spot size <100µm)

**x**  line scan (lateral resolution <100µm)

**x**  elemental 2D mapping

  quantification, MQuant Calib, Bruker; Berlin, Germany

  quantification with standards

**Voltage** 50 KV

**Current** 600 µA

**Filter x**  no filter   Al 315 µm   Mo 12.50 µm   other ______

**Lens** 0.060

**Atmosphere x**  air   He for light element detection

**See tables in Result section for details of scan time per point, number of measurement points, spot distance, scan area and total scan time.**

## Notes for interpretation

- Molybdenum and argon are associated with the XRF instrument and are present in every spectrum.
- The Mo L line overlaps with the S K line making an unambiguous detection of sulphur difficult.
- Automatic corrections were performed for escape peaks and background using cycle setting 1. Note that some images of spectra show a wrong background line where the cycle setting was accidentally left at 40, this was later corrected and all peak area calculations were carried out with background cycle setting 1.
- Peak areas were calculated from accumulated spectra. Most line scans contained five points for the accumulation, where there were fewer or more points or an obvious outlier a corrected spectrum was calculated adjusting the number of scans to five. This is indicated in the results table where applicable. The live time was 10 s for all spectra used in the accumulation spectra for semi quantification.
- Net peak areas were calculated using ROI (regions of interest) rather than the automatic deconvolution function. The start and end keV for each ROI was set as follows:
  - Start/keV End/keV Peak name
  - 2,179 2,444 S_K
  - 2,449 2,737 Cl_K
  - 2,774 3,115 Ar_K
  - 3,099 3,492 K_K
  - 3,484 3,873 Ca_K
  - 5,698 6,07 Mn_K
  - 6,082 6,698 Fe_K
  - 7,789 8,327 Cu_K
  - 8,425 8,836 Zn_K
  - 15,774 17,128 Compton
  - 17,155 17,797 Mo_K
  - 10,183 10,871 Pb_L
  - 7,307 7,672 Ni_K
  - 9,799 10,175 Hg_L
  - 1,907 2,099 P_K
  - 1,642 1,847 Si_K
- Mapping result images give relative quantitative information in greyscale for every element detected where black represents the lowest levels detected and white indicates the highest levels. The minimum and maximum net peak areas are shown on the right of the images; these numbers give an indication of the overall variation for each element. Together with the accumulated spectrum they also show whether an element is present in significant amounts or near background levels.
- In calculating net peak areas values are obtained even for non-existent peaks as some counts are present between the noise of the spectral line and the generated background line. In interpreting the results table it is important to cross reference results with the spectra to check whether a peak is actually present or not. The difference between noise and peak is not always clear and a cut-off value cannot be set as the noise counts from a spectrum with a high background (high bremsstrahlung) may be higher than counts from a real peak in a spectrum with a low background.
- The detector’s deadtime varied mostly between 5-9%. For some spectra this was significantly lower and seemed to result in erroneous values with lower than expected counts. For two areas (papers for K394_0014 and K429_3767) the net counts are shown in grey in the results table and were subsequently not included in any results interpretations.
- Certain papers induce high bremsstrahlung scattering (high background) which can present difficulties for the quality of fit of the background line, in some cases leading to overestimation of net peak areas for elements that are present near background level.
- Attempts were made to quantify the metals in relation to paper and the levels of minor components (Mn, Cu, Zn) in relation to iron in the inks, see 3.5.1-04051-2017 instrument report quantitative analysis.
- The overall count rate may be affected by variations in the paper, the composition of the inks and/or impregnation materials (matrix effects) and by variations in detector deadtime. This can be normalised by dividing the net counts of the peaks of interest by the net counts of the Compton peak. The Compton peak is a part of the Bremsstrahlung background.
- Due to inhomogeneities in the paper structure and variations in ink line thickness, comparison of XRF data from individual element peaks in different documents might present difficulties. It is however possible to semi-quantitatively analyse the composition of inks within one document and thereby draw some conclusions regarding the content of other elements relative to iron. In order to get an idea of the relative proportions of components in the individual inks, the net peak area counts of each element have been normalized to Compton and compared with that of iron. The results are plotted below in the section Results graphs, where the iron content has been reduced by x10 in order to improve legibility of the graph which, bearing this fact in mind, presents a collection of ink lines containing significant amounts of iron but small or no amount of other metal elements.

# Results (spectra, line scans and maps)

## Del Monte, Montanari – (12/9 1687), Vol. II, A:1 nr 17, Christina (?), good condition

| 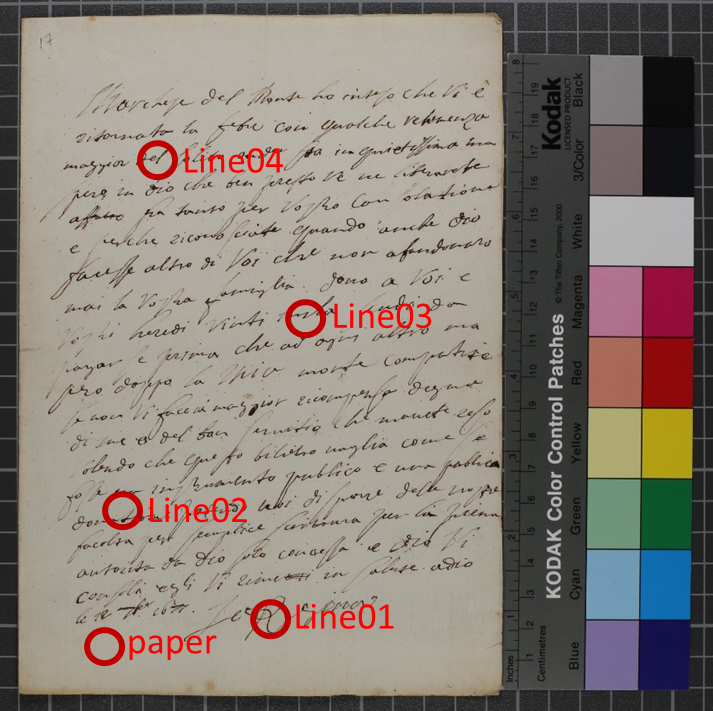 | Analysis area | Comment | Instrument settings |
| --- | --- | --- | --- |
|  | Line 01 | ink thickness 2, Christina (?) | 5 points, 10 s live time, 0.26 mm spot distance, 1.04 mm length |
|  | Line 02 | ink thickness 3, Christina (?) | 5 points, 10 s live time, 0.17 mm spot distance, 0.68 mm length |
|  | Line 03 | ink thickness 2, Christina (?) | 5 points, 10 s live time, 0.25 mm spot distance, 1.00 mm length |
|  | Line 04 | ink thickness 2, Christina (?) | 5 points, 10 s live time, 0.16 mm spot distance, 0.64 mm length |
|  | paper |  | 5 points, 10 s live time, 0.16 mm spot distance, 0.64 mm length |

### Del Monte, Montanari Vol. II, A:1 nr 17 line 01

|   Del Monte, Montanari Vol. II, A:1 nr 17 line 01 accumulated spectrum    Del Monte, Montanari Vol. II, A:1 nr 17 line 01 detail overlay of points 01 – 05 | 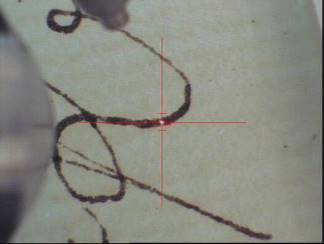  Point 01  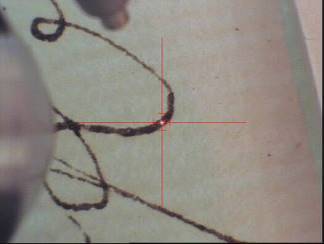  Point 05 |
| --- | --- |

### Del Monte, Montanari Vol. II, A:1 nr 17 line 02

| ****  Del Monte, Montanari Vol. II, A:1 nr 17 line 02 accumulated spectrum  ****  Del Monte, Montanari Vol. II, A:1 nr 17 line 02 detail overlay of points 01 – 05 | 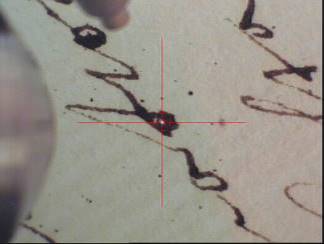  Point 01  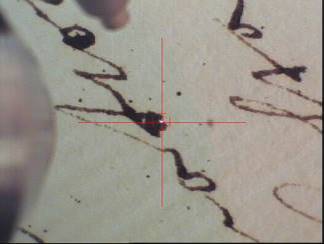  Point 05 |
| --- | --- |

### Del Monte, Montanari Vol. II, A:1 nr 17 line 03

| ****  Del Monte, Montanari Vol. II, A:1 nr 17 line 03 accumulated spectrum  ****  Del Monte, Montanari Vol. II, A:1 nr 17 line 03 detail overlay of points 01 – 05 | 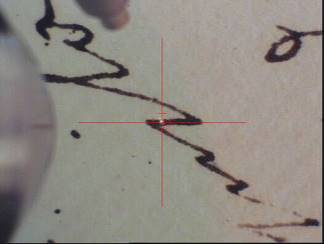  Point 01  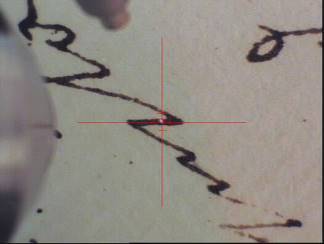  Point 05 |
| --- | --- |

### Del Monte, Montanari Vol. II, A:1 nr 17 line 04

| ****  Del Monte, Montanari Vol. II, A:1 nr 17 line 04 accumulated spectrum  ****  Del Monte, Montanari Vol. II, A:1 nr 17 line 04 detail overlay of points 01 – 05 | 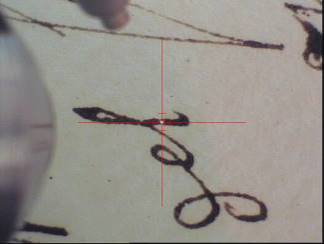  Point 01  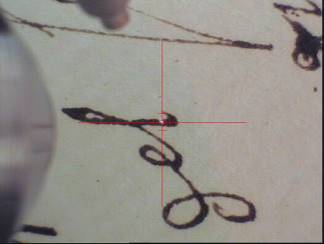  Point 05 |
| --- | --- |

### Del Monte, Montanari Vol. II, A:1 nr 17 paper

| ****  Del Monte, Montanari Vol. II, A:1 nr 17 paper accumulated spectrum    Del Monte, Montanari Vol. II, A:1 nr 17 paper detail overlay of points 01 – 05 | 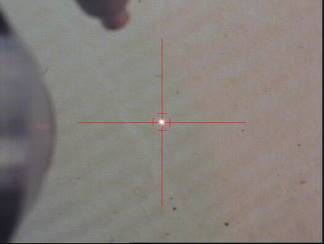  Point 01  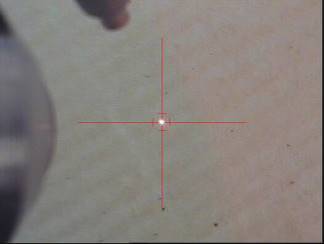  Point 05 |
| --- | --- |

## K394_0014, fair condition, Christina, Hamburg, 1666

| 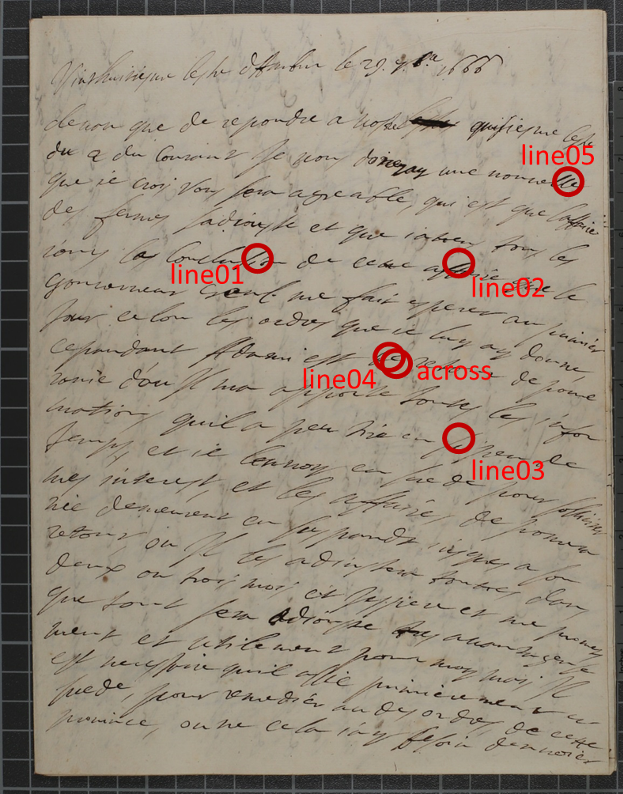 | Analysis area | Comment | Instrument settings |
| --- | --- | --- | --- |
|  | Line 01 | ink thickness 2 | 5 points, 10 s live time, 0.43 mm spot distance, 1.72 mm length |
|  | Line 02 | ink thickness 3 | 5 points, 10 s live time, 0.63 mm spot distance, 2.52 mm length |
|  | Line 03 | ink thickness 1 | 5 points, 10 s live time, 0.63 mm spot distance, 2.52 mm length |
|  | Line 04 | ink thickness 2 | 5 points, 10 s live time, 0.34 mm spot distance, 1.36 mm length |
|  | Line 05 | ink thickness 2 | 5 points, 10 s live time, 0.23 mm spot distance, 0.92 mm length |
|  | across | ink thickness 2 (paper, burnthrough, paper, ink, paper) | 51 points, 10 s live time, 0.05 mm spot distance, 2.50 mm length |
|  | paper | on folded back page of letter circa 1.5 cm in from lower right corner | 5 points, 10 s live time, 0.23 mm spot distance, 0.92 mm length |

### K394_0014 line 01

| ****  K394_0014 line 01 accumulated spectrum    K394_0014 line 01 detail overlay of points 01 – 05  ****  K394_0014 line 01 detail overlay of points 01 – 05 | 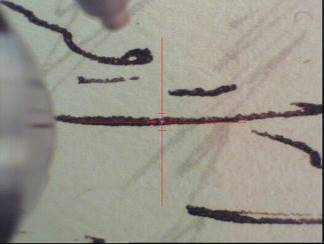  Point 01  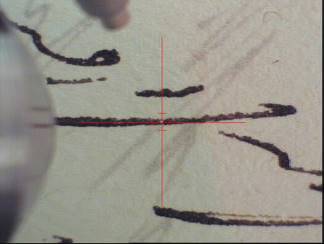  Point 05 |
| --- | --- |

### K394_0014 line 02

| ****  K394_0014 line 02 accumulated spectrum  ****  K394_0014 line 02 detail overlay of points 01 – 05  ****  K394_0014 line 02 detail overlay of points 01 – 05 | 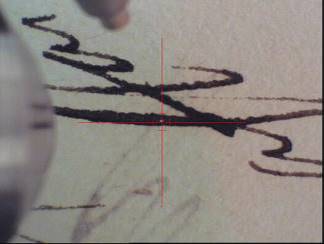  Point 01  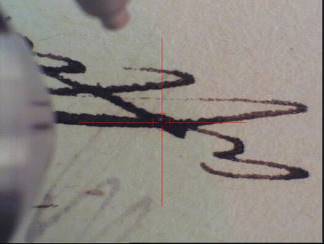  Point 05 |
| --- | --- |

### K394_0014 line 03

| ****  K394_0014 line 03 accumulated spectrum  ****  K394_0014 line 03 detail overlay of points 01 – 05 | 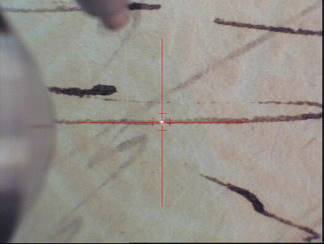  Point 01  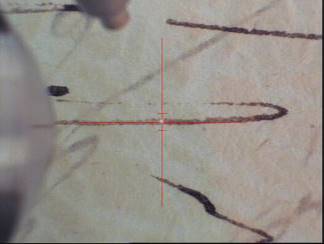  Point 05 |
| --- | --- |

### K394_0014 line 04

| ****  K394_0014 line 04 accumulated spectrum  ****  K394_0014 line 04 detail overlay of points 01 – 05 | 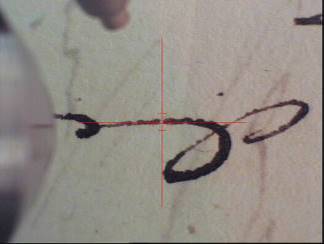  Point 01  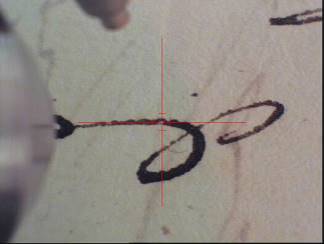  Point 05 |
| --- | --- |

### K394_0014 across

| ****  K394_0014 across accumulated spectrum  **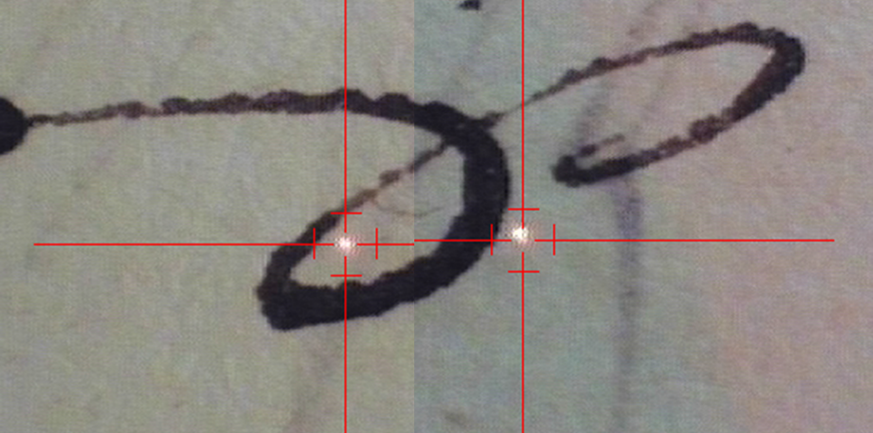**  Image of line scan area from point 01 to point 51 (with laser focus spots in the cross-hairs)  **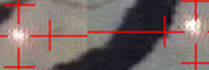**  Image of line scan area from point 01 to point 51 (with laser focus spots in the cross-hairs) | 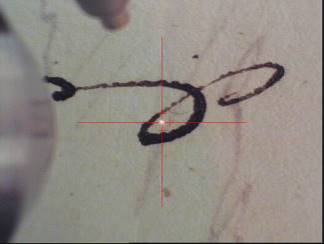  Point 01  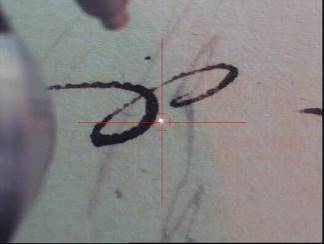  Point 51 |
| --- | --- |
| **** | Linescan results overview |
| **** | Linescan results detail  Note: raised iron detection in area of burnthrough. |

### K394_0014 line 05

| ****  K394_0014 line 05 accumulated spectrum  ****  K394_0014 line 05 detail overlay of points 01 – 05 | 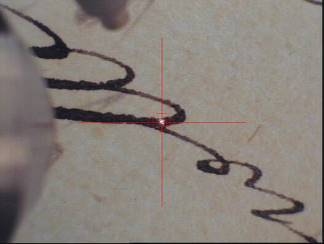  Point 01  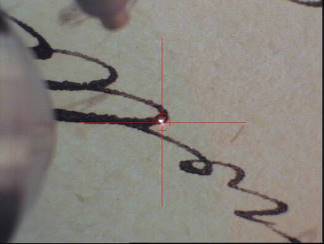  Point 05 |
| --- | --- |

### K394_0014 paper

| ****  K394_0014 paper accumulated spectrum    K394_0014 paper detail overlay of points 01 – 05  ****  K394_0014 paper detail overlay of points 01 – 05 | 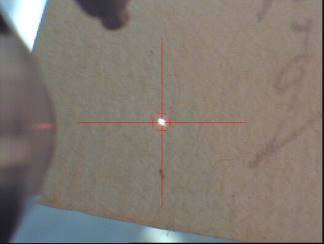  Point 01  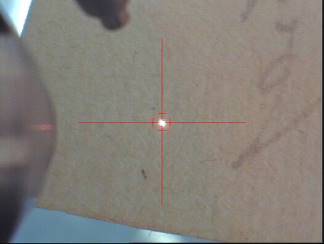  Point 05  Note: Cu and Zn were just discernible in the accumulated spectrum but not in the individual spectra.  All five spectra have very low counts; ca. 10 to 20 times lower than usual. No instrument settings were changed between this analysis and earlier lines on the same paper. However, the spectral parameters show that the detector deadtime was less than 1% when it is normally around 5-9%. |
| --- | --- |

## K396_266, Rome, April 1666, fair condition

| 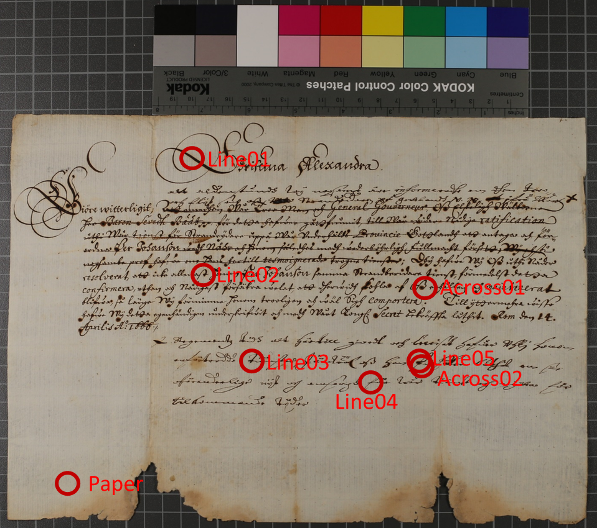 | Analysis area | Comment | Instrument settings |
| --- | --- | --- | --- |
|  | line01 | Ink thickness 4 | 5 points, 10 s live time,  0.24 mm spot distance, 0.91 mm length |
|  | line02 | Ink thickness 2 | 5 points, 10 s live time,  0.19 mm spot distance, 0.76 mm length |
|  | Across01 | Ink thickness 4, halo | 82 points, 10 s live time, 0.04 spot distance, 3.24 mm |
|  | line03 | Ink thickness 3 | 5 points, 10 s live time,  0.22 mm spot distance, 0.88 mm length |
|  | line04 | Ink thickness 2 | 5 points, 10 s live time,  0.15 mm spot distance, 0.60 mm length |
|  | Line05 | Ink thickness 3 | 5 points, 10 s live time, 0.15 mm spot distance, 0.60 mm length |
|  | Across02 | Ink thickness 3, halo | 49 points, 10 s live time, 0.03 spot distance, 1.44 mm |
|  | paper |  | 5 points, 10 s live time, 0.21 mm spot distance, 0.84 mm length |

### K396_266 line 01

| ****  K396_266 line 01 accumulated spectrum  ****  K396_266 line 01 detail overlay of points 01 – 05 | 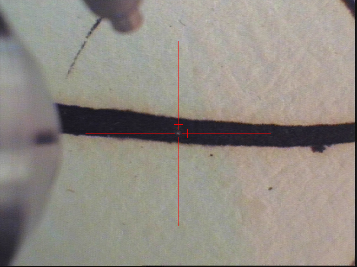  Point 01  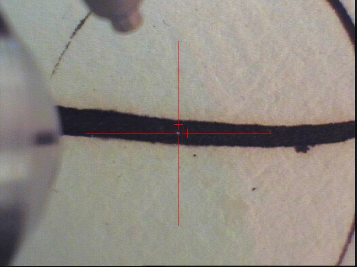  Point 05 |
| --- | --- |

### K396_266 line 02

| ****  K396_266 line 02 accumulated spectrum  ****  K396_266 line 02 detail overlay of points 01 – 05 | 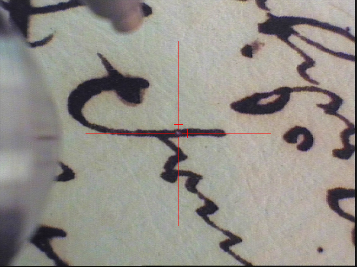  Point 01  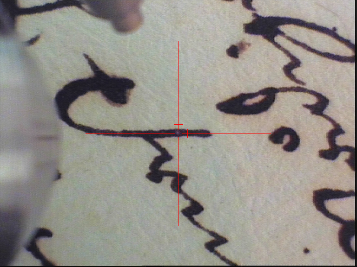  Point 05 |
| --- | --- |

### K396_266 line 03

| ****  K396_266 line 03 accumulated spectrum  ****  K396_266 line 03 detail overlay of points 01 – 05 | 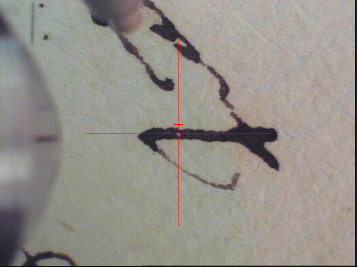  Point 01  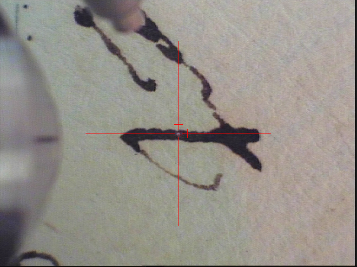  Point 05 |
| --- | --- |

### K396_266 line 04

| ****  K396_266 line 04 accumulated spectrum  ****  K396_266 line 04 detail overlay of points 01 – 05 | 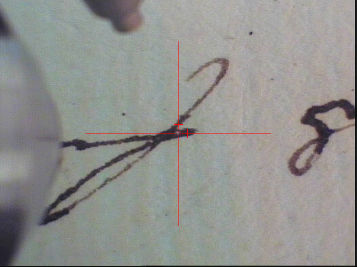  Point 01  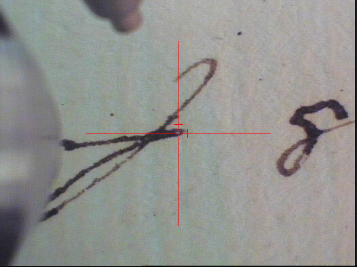  Point 05 |
| --- | --- |

### K396_266 line 05

| ****  K396_266 line 05 accumulated spectrum  ****  K396_266 line 05 detail overlay of points 01 – 05  **** | 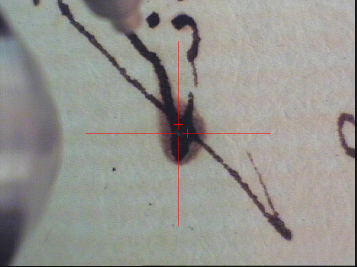  Point 01  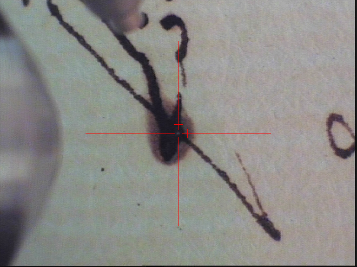  Point 05  Note:  Hg was only detected in one of the five points. A corrected accumulated spectrum without mercury was also calculated, using another point twice to accumulate a total of five points. |
| --- | --- |

### K396_266 across01

| K396_266 across01 accumulated spectrum **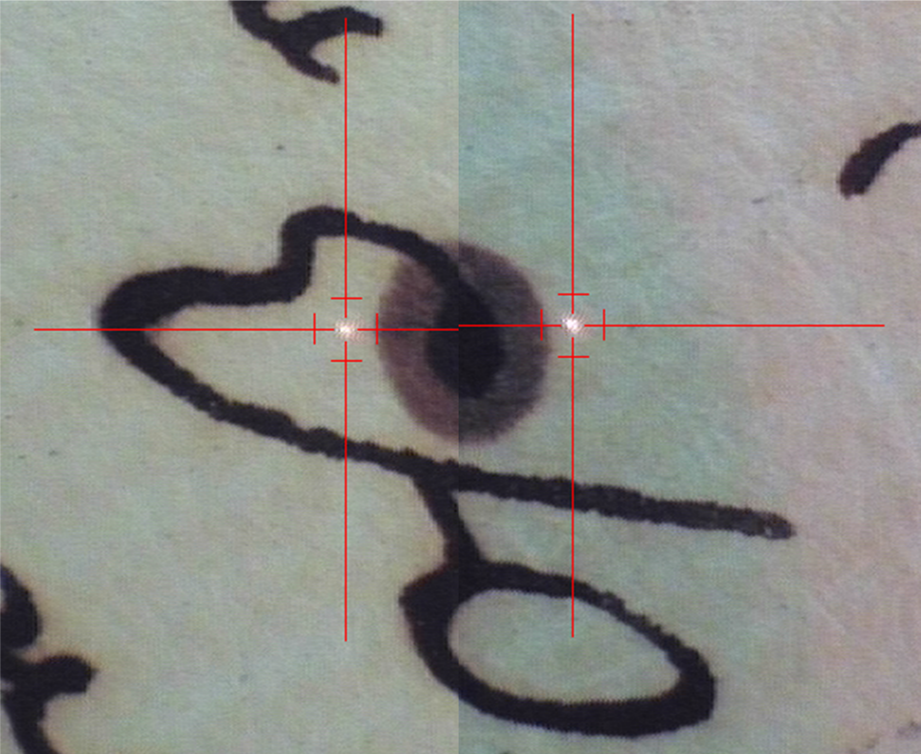**  Image of line scan area from point 01 to point 82 (with laser focus spots in the cross-hairs)  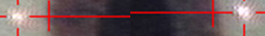  Image of line scan area from point 01 to point 82 (with laser focus spots in the cross-hairs) | 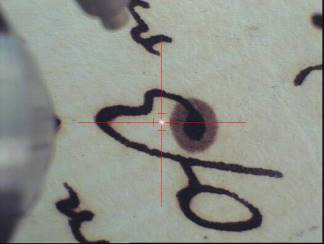  Point 01  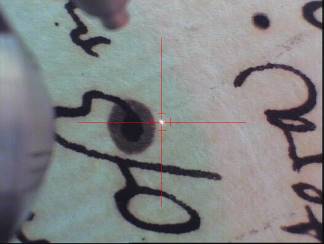  Point 82 |
| --- | --- |
| **** | Linescan results overview |
| **** | Linescan results detail |

### K396_266 paper

| ****  K396_266 paper accumulated spectrum  ****  K396_266 paper detail overlay of points 01 – 05 | 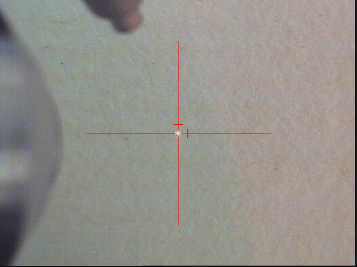  Point 01  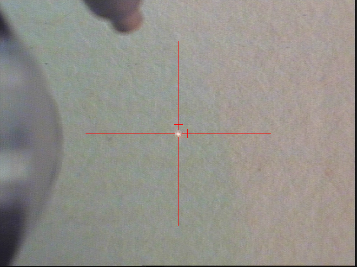  Point 05 |
| --- | --- |

## K397_364, Stropp, 1662, Stockholm, good condition

| 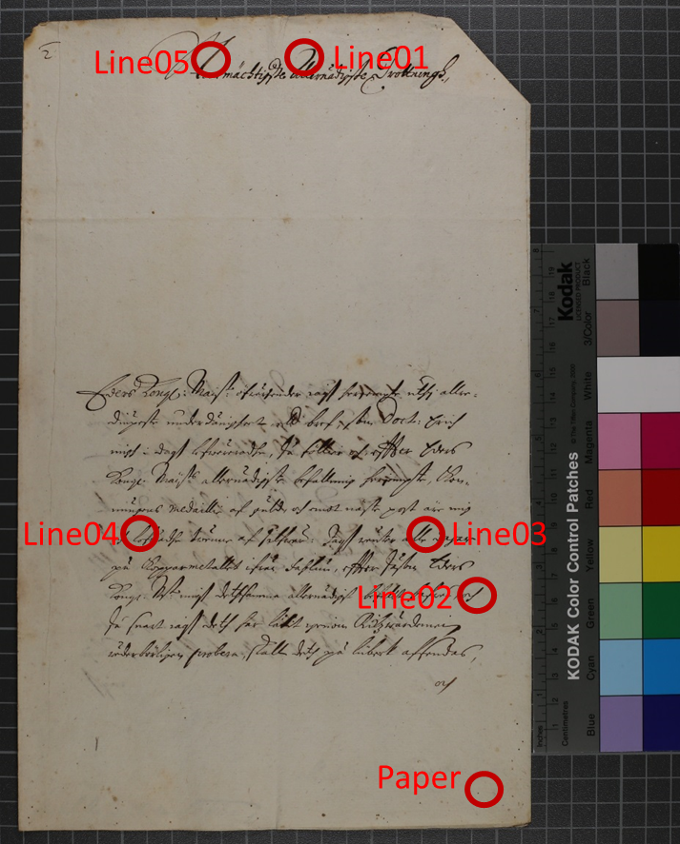 | Analysis area | Comment | Instrument settings |
| --- | --- | --- | --- |
|  | Line 01 | ink thickness 3 | 5 points, 10 s live time, 0.26 mm spot distance, 1.04 mm length |
|  | Line 02 | ink thickness 2 | 5 points, 10 s live time, 0.26 mm spot distance, 1.04 mm length |
|  | Line 03 | ink thickness 3 | 5 points, 10 s live time, 0.26 mm spot distance, 1.04 mm length |
|  | Line 04 | ink thickness 1 | 5 points, 10 s live time, 0.23 mm spot distance, 0.92 mm length |
|  | Line 05 | ink thickness 2 | 5 points, 10 s live time |
|  | paper |  | 5 points, 10 s live time, 0.32 mm spot distance, 1.28 mm length |

### K397_364 line 01

| ****  K397_364 line 01 accumulated spectrum  ****  K397_364 line 01 detail overlay of points 01 – 05  ****  K397_364 line 01 detail overlay of points 01 – 05 | 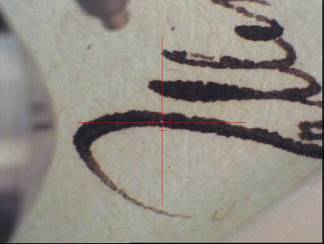  Point 01  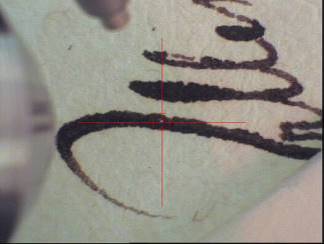  Point 05 |
| --- | --- |

### K397_364 line02

| ****  K397_364 line02 accumulated spectrum  ****  K397_364 line02 detail overlay of points 01 – 05 | 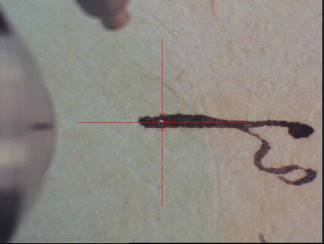  Point 01  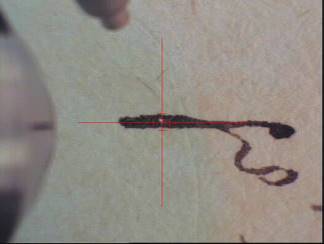  Point 05  Note:  Zn was just noticeable near background level in the accumulated spectrum. |
| --- | --- |

### K397_364 line03

| ****  K397_364 line03 accumulated spectrum  ****  K397_364 line03 detail overlay of points 01 – 05 | 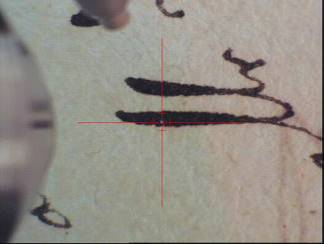  Point 01  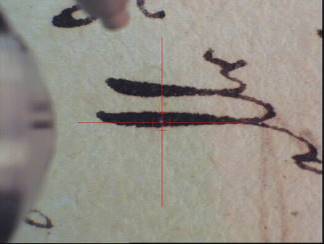  Point 05 |
| --- | --- |

### K397_364 line04

| ****  K397_364 line04 accumulated spectrum  ****  K397_364 line04 detail overlay of points 01 – 05 | 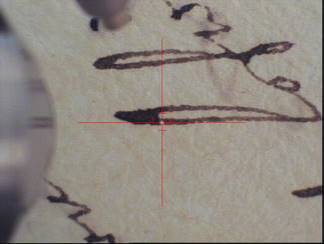  Point 01  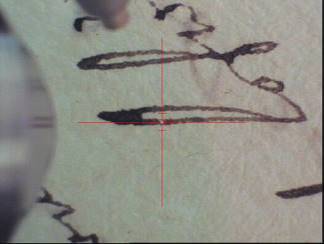  Point 05  Note:  Zn was just noticeable near background level in the accumulated spectrum. |
| --- | --- |

### K397_364 line05

| ****  K397_364 line05 accumulated spectrum  ****  K397_364 line05detail overlay of points 01 – 05 | 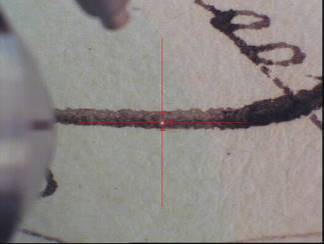  Point 01  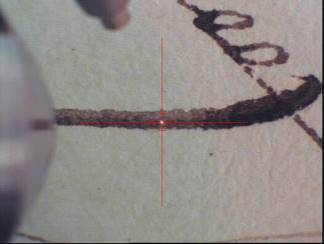  Point 05  Note:  Zn was just noticeable near background level in the accumulated spectrum. |
| --- | --- |

### K397_364 paper

| ****  K397_364 paper accumulated spectrum  ****  K397_364 paper detail overlay of points 01 – 05 | 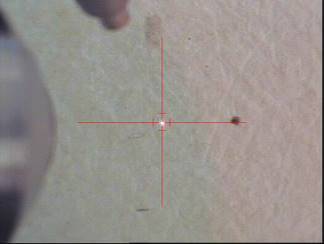  Point 01  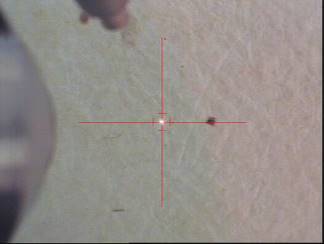  Point 05 |
| --- | --- |

## K399_470, Niclas Marcus, 31 March 1676, Visby, good condition

| 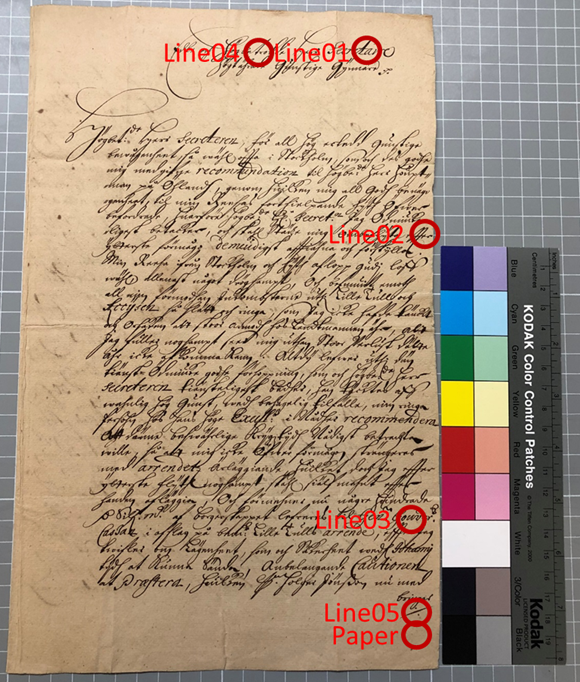 | Analysis area | Comment | Instrument settings |
| --- | --- | --- | --- |
|  | line01 | ink thickness 3 | 5 points, 10 s live time, 0.20 mm spot distance, 0.80 mm length |
|  | line02 | ink thickness 2 | 5 points, 10 s live time, 0.20 mm spot distance, 0.80 mm length |
|  | line03 | ink thickness 2 | 5 points, 10 s live time, 0.20 mm spot distance, 0.80 mm length |
|  | line04 | ink thickness 2 | 5 points, 10 s live time, 0.26 mm spot distance, 1.04 mm length |
|  | Line05 | ink thickness 2 | 5 points, 10 s live time, 0.26 mm spot distance, 1.04 mm length |
|  | paper |  | 5 points, 10 s live time, 0.26 mm spot distance, 1.04 mm length |

### K399_470 line01

| ****  K399_470 line01 accumulated spectrum    K399_470 line01 detail overlay of points 01 – 05 | 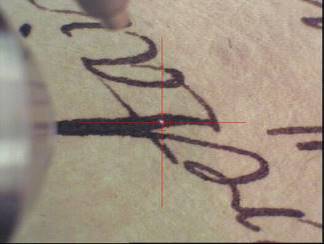  Point 01  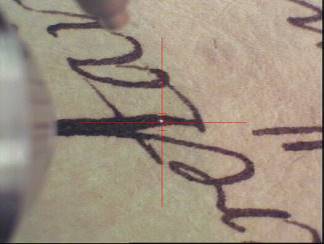  Point 05 |
| --- | --- |

### K399_470 line02

| ****  K399_470 line02 accumulated spectrum    K399_470 line02 detail overlay of points 01 – 05 | 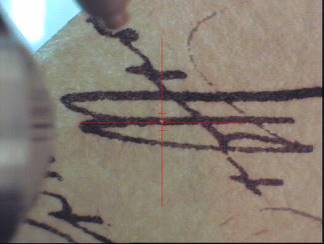  Point 01  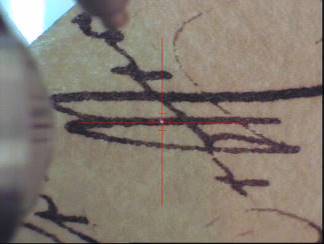  Point 05 |
| --- | --- |

### K399_470 line03

| ****  K399_470 line03 accumulated spectrum    K399_470 line03 detail overlay of points 01 – 05 | 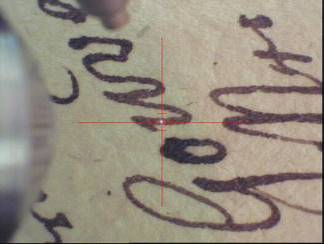  Point 01  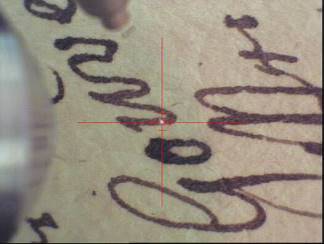  Point 05 |
| --- | --- |

### K399_470 line04

| ****  K399_470 line04 accumulated spectrum    K399_470 line04 detail overlay of points 01 – 05 | 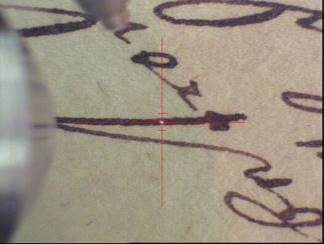  Point 01  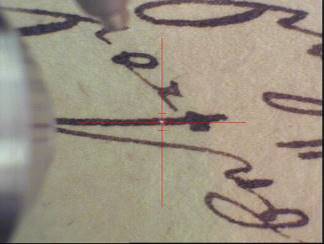  Point 05 |
| --- | --- |

### K399_470 line05

| ****  K399_470 line05 accumulated spectrum    K399_470 line05 detail overlay of points 01 – 05 | 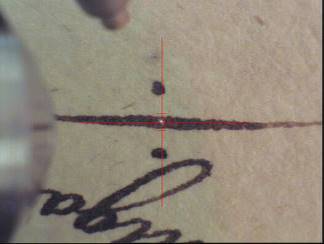  Point 01  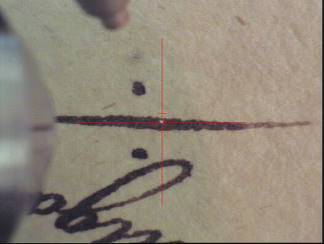  Point 05 |
| --- | --- |

### K399_470 paper

| ****  K399_470 paper accumulated spectrum    K399_470 paper detail overlay of points 01 – 05 | 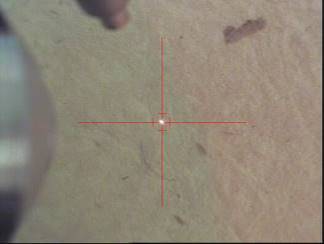  Point 01  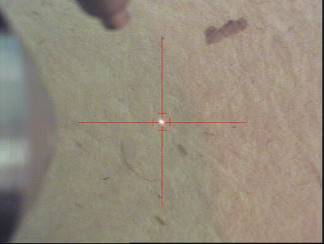  Point 05 |
| --- | --- |

## K401_658, Seved Bååt, 19 Jan 1659, Stockholm, fair condition

| 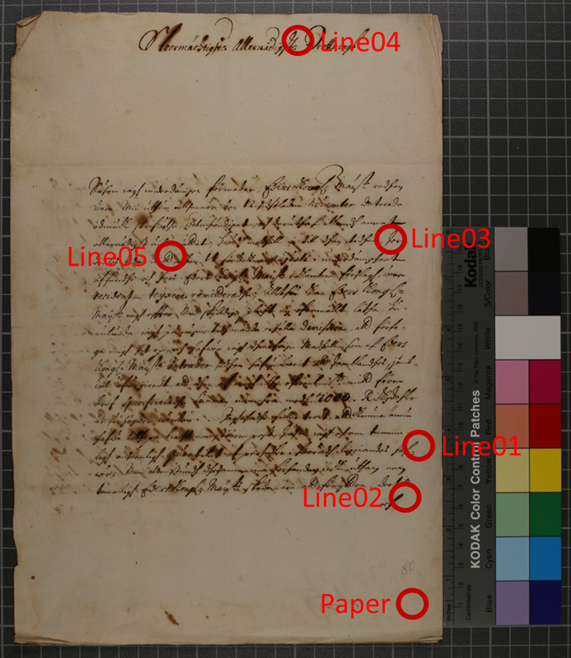 | Analysis area | Comment | Instrument settings |
| --- | --- | --- | --- |
|  | line01 | ink thickness 2 | 5 points, 10 s live time, 0.18 mm spot distance, 0.72 mm length |
|  | line02 | ink thickness 4 | 5 points, 10 s live time, 0.28 mm spot distance, 1.12 mm length |
|  | line03 | ink thickness 2 | 5 points, 10 s live time, 0.15 mm spot distance, 0.60 mm length |
|  | line04 | ink thickness 3 | 5 points, 10 s live time, 0.18 mm spot distance, 0.72 mm length |
|  | Line05 | ink thickness 2 | 5 points, 10 s live time, 0.26 mm spot distance, 1.04 mm length |
|  | paper |  | 5 points, 10 s live time, 0.26 mm spot distance, 1.04 mm length |

### K401_658 line01

| ****  K401_658 line01 accumulated spectrum  ****  K401_658 line01 detail overlay of points 01 – 05 | 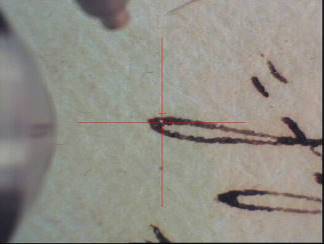  Point 01  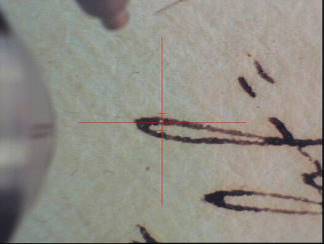  Point 05  Detector dead time was low at 3.7%. |
| --- | --- |

### K401_658 line02

| ****  K401_658 line02 accumulated spectrum  ****  K401_658 line02 detail overlay of points 01 – 05 | 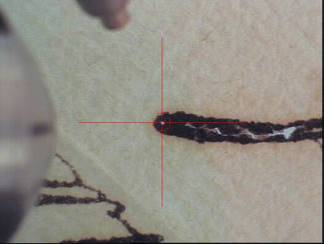  Point 01  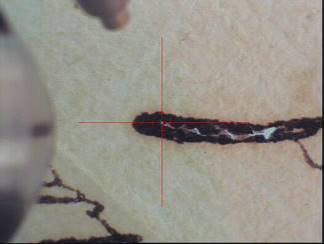  Point 05 |
| --- | --- |

### K401_658 line03

| ****  K401_658 line03 accumulated spectrum  ****  K401_658 line03 detail overlay of points 01 – 05 | 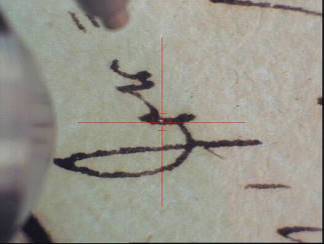  Point 01  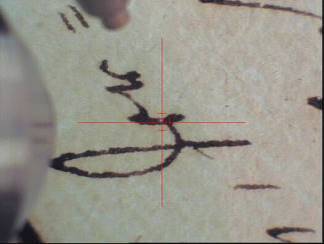  Point 05 |
| --- | --- |

### K401_658 line04

| ****  K401_658 line04 accumulated spectrum  ****  K401_658 line04 detail overlay of points 01 – 05 | 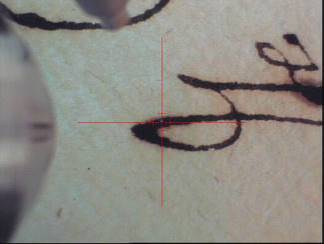  Point 01  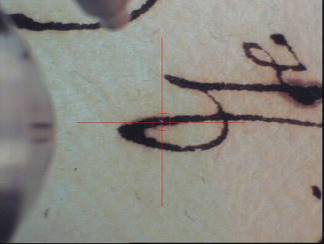  Point 05 |
| --- | --- |

### K401_658 line05

| ****  K401_658 line05 accumulated spectrum  ****  K401_658 line05 detail overlay of points 01 – 05 | 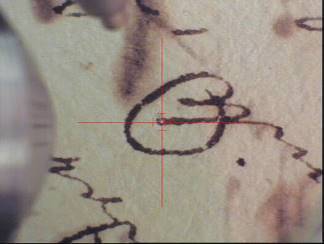  Point 01  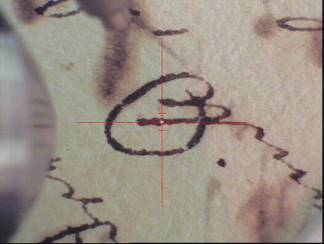  Point 05 |
| --- | --- |

### K401_658 paper

| ****  K401_658 paper accumulated spectrum  ****  K401_658 paper detail overlay of points 01 – 05 | 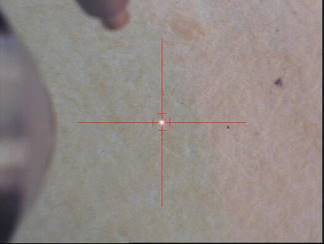  Point 01  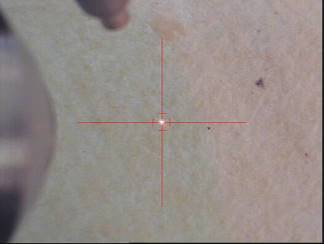  Point 05 |
| --- | --- |

## K403_828, page 1: Azzolino and page 2: Christina (?), good condition, 1665

| Analysis area | Comment | Instrument settings |
| --- | --- | --- |
| Page 1 line 01 | ink thickness 2, Azzolino | 5 points, 10 s live time, 0.17 mm spot distance, 0.68 mm length |
| Page 1 line 02 | ink thickness 1, Azzolino | 5 points, 10 s live time, 0.28 mm spot distance, 1.21 mm length |
| Page 1 line 03 | ink thickness 3, Azzolino | 5 points, 10 s live time, 0.25 mm spot distance, 1 mm length |
| Page 2 line 04 | ink thickness 1, Christina (?) | 5 points, 10 s live time, 0.18 mm spot distance, 0.72 mm length |
| Page 2 line 05 | ink thickness 3, Christina (?) | 5 points, 10 s live time, 0.19 mm spot distance, 0.76 mm length |
| Page 2 line 06 | ink thickness 2, Christina (?) | 5 points, 10 s live time, 0.18 mm spot distance, 0.72 mm length |
| Page 1 paper |  | 5 points, 10 s live time, 0.27 mm spot distance, 1.08 mm length |
| Page 2 map |  | 6532 points, 3 s live time, 0.10 mm spot distance, 7 mm x 9.1 mm area, total scan time 16:19 h |

**
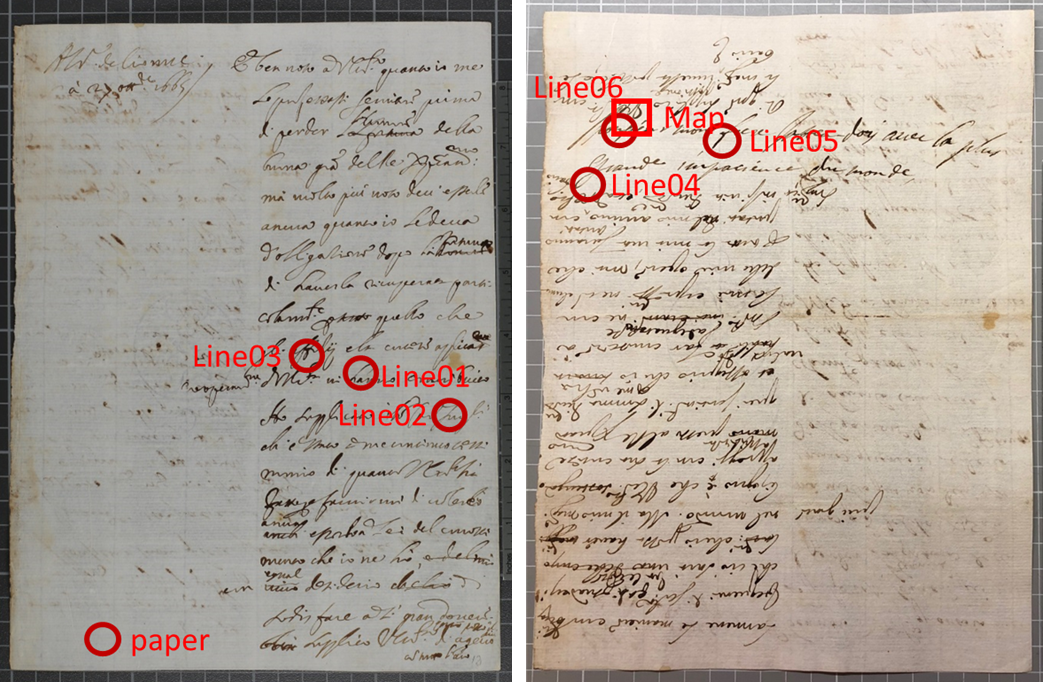
**

### K403_828 page 1 line 01

| ****  K403_828 page 1 line 01 accumulated spectrum  ****  K403_828 page 1 line 01 detail overlay of points 01 – 05 | 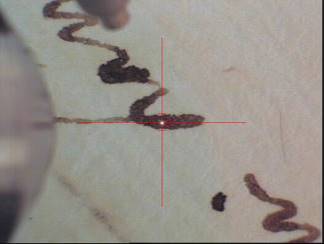  Point 01  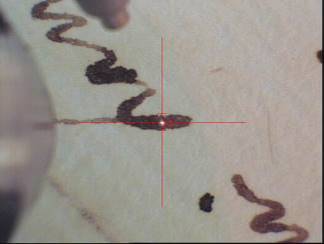  Point 05 |
| --- | --- |

### K403_828 page 1 line 02

|   K403_828 page 1 line 02 accumulated spectrum    K403_828 page 1 line 02 detail overlay of points 01 – 05 | 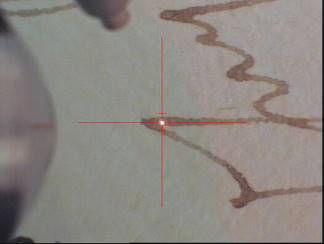  Point 01  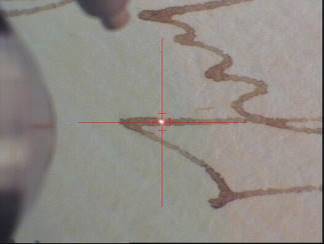  Point 05 |
| --- | --- |

### K403_828 page 1 line 03

| ****  K403_828 page 1 line 03 accumulated spectrum  ****  K403_828 page 1 line 03 detail overlay of points 01 – 05 | 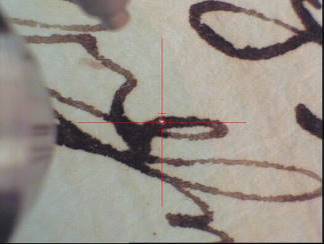  Point 01  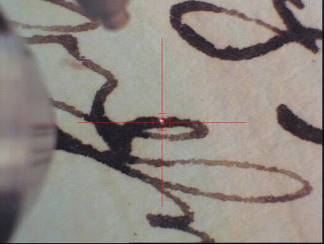  Point 05 |
| --- | --- |

### K403_828 page 2 line 04

| ****  K403_828 page 2 line 04 accumulated spectrum  ****  K403_828 page 2 line 04 detail overlay of points 01 – 05 | 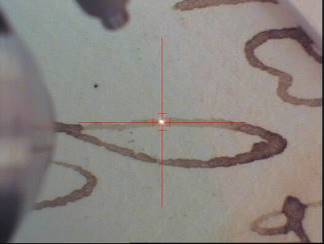  Point 01  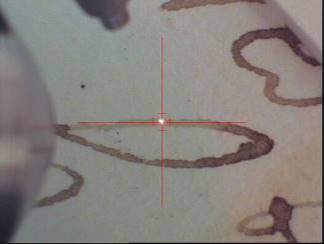  Point 05 |
| --- | --- |

### K403_828 page 2 line 05

| ****  K403_828 page 2 line 05 accumulated spectrum    K403_828 page 2 line 05 detail overlay of points 01 – 05 | 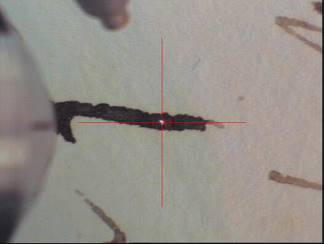  Point 01  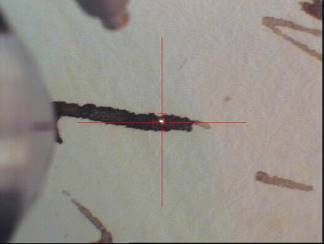  Point 05 |
| --- | --- |

### K403_828 page 2 line 06

| ****  K403_828 page 2 line 06 accumulated spectrum  ****  K403_828 page 2 line 06 detail overlay of points 01 – 05 | 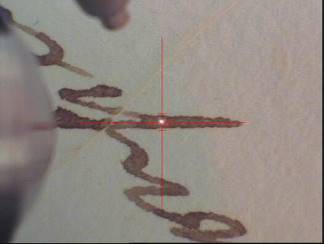  Point 01  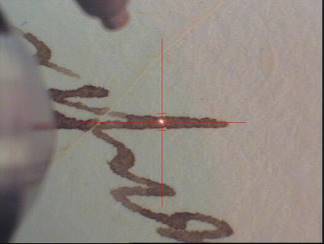  Point 05 |
| --- | --- |

### K403_828 page 1 paper

| ****  K403_828 page 1 paper accumulated spectrum  ****  K403_828 page 1 paper detail overlay of points 01 – 05 | 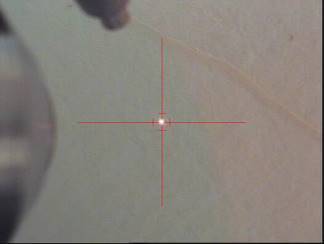  Point 01  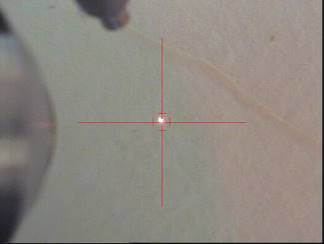  Point 05 |
| --- | --- |

### K403_828 page 2 map

| **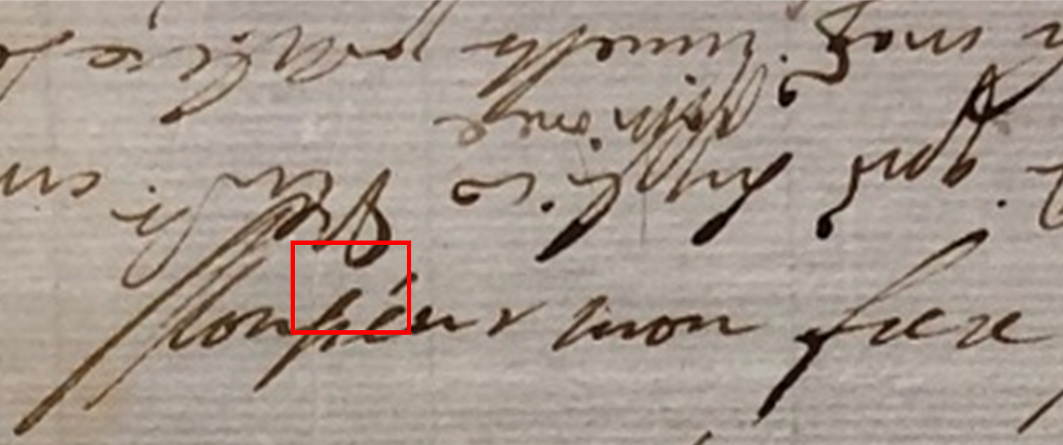**  K403_828 page 2 with XRF map area marked in red    K403_828 page 2 map accumulated spectrum    K403_828 page 1 map | Point 01    Point 92    Point 6441    Point 6532 |
| --- | --- |

| K403_828 page 2 XRF mapping results with separate images for the detected elements Cl, K, Ca and Fe. | |
| --- | --- |
|  |  |
| Note: S and Mo overlap in the XRF spectrum which makes detection of sulphur difficult. Molybdenum is instrument inherent due to the incident Mo X-rays. The major Mo K peak did not show any particular map distribution while the Mo L map shows accumulation as tidelines around the edges of the ink; this could be attributable to sulphur. | |

## K405_1092, Texeira, June 1670, Hamburg, good condition

|  | Analysis area | Comment | Instrument settings |
| --- | --- | --- | --- |
|  | Line 01 | ink thickness 2 | 5 points, 10 s live time, 0.18 mm spot distance, 0.72 mm length |
|  | Line 02 | ink thickness 1 | 5 points, 10 s live time, 0.24 mm spot distance, 0.96 mm length |
|  | Line 03 | ink thickness 2 | 5 points, 10 s live time, 0.20 mm spot distance, 0.80 mm length |
|  | Line 04 | Page 3, ink thickness 1 | 5 points, 10 s live time, 0.20 mm spot distance, 0.80 mm length |
|  | Line 05 | Page 3, ink thickness 2 | 5 points, 10 s live time, 0.21 mm spot distance, 0.84 mm length |
|  | paper |  | 5 points, 10 s live time, 0.20 mm spot distance, 0.80 mm length |
|  | | | |

### K405_1092 line01

| K405_1092 line 01 accumulated spectrum    K405_1092 line 01 detail overlay of points 01 – 05 | Point 01    Point 05 |
| --- | --- |

### K405_1092 line 02

| K405_1092 line 02 accumulated spectrum    K405_1092 line 02 accumulated spectrum without point 3 (high Ca and P)    K405_1092 line 02 detail overlay of points 01 – 05    K405_1092 line 02 detail overlay of points 01 – 05 | Point 01    Point 05  Note: only one of the five points shows an unusually high peak for calcium and a small peak for phosphorous. A corrected accumulated spectrum was calculated with this outlier removed. |
| --- | --- |

### K405_1092 line 03

| K405_1092 line 03 accumulated spectrum    K405_1092 line 03 detail overlay of points 01 – 05    K405_1092 line 03 detail overlay of points 01 – 05 | Point 01    Point 05  Note:  One point showed an unusually high calcium peak. Phosphorous was discernible near background level in the accumulated spectrum but not in the individual points. |
| --- | --- |

### K405_1092 line 04

| K405_1092 line 04 accumulated spectrum    K405_1092 line 04 detail overlay of points 01 – 05 | Point 01    Point 05 |
| --- | --- |

### K405_1092 line 05

| K405_1092 line 05 accumulated spectrum    K405_1092 line 05 detail overlay of points 01 – 05    K405_1092 line 05 detail overlay of points 01 – 05 | Point 01    Point 05  Note:  One point showed an unusually high calcium peak. |
| --- | --- |

### K405_1092 paper

| K405_1092 paper accumulated spectrum    K405_1092 paper detail overlay of points 01 – 05 | Point 01    Point 05 |
| --- | --- |

## K407_1298, good condition, Christina, Rome, 1677

|  | Analysis area | Comment | Instrument settings |
| --- | --- | --- | --- |
|  | line01 | ink thickness 2 | 6 points, 10 s live time, 0.16 mm spot distance, 0.80 mm length |
|  | line02 | ink thickness 4 | 5 points, 10 s live time, 0.20 mm spot distance, 0.80 mm length |
|  | line03 | ink thickness 2 | 12 points, 10 s live time, 0.09 mm spot distance, 0.99 mm length |
|  | line04 | ink thickness 1 | 7 points, 10 s live time, 0.09 mm spot distance, 0.54 mm length |
|  | line05 | ink thickness 2 | 5 points, 10 s live time, 0.23 mm spot distance, 0.92 mm length |
|  | across | paper, halo, ink thickness 4 | 54 points, 10 s live time, 0.09 mm spot distance, 4.77 mm length |
|  | paper |  | 5 points, 10 s live time, 0.21 mm spot distance, 0.84 mm length |

### K407_1298 line 01

| K407_1298 line 01 accumulated spectrum    K407_1298 line 01 detail overlay of points 01 – 06    K407_1298 line 01 detail overlay of points 01 - 06 | Point 01    Point 06  Note:  Zn is not detected in the individual points of analyses. The small raise in background noticed in the accumulated spectrum is most likely an artefact. Cu and Mn are indicated just above the limit of detection.  A corrected accumulated spectrum of just five points was also calculated. |
| --- | --- |

### K407_1298 line 02

| K407_1298 line 02 accumulated spectrum    K407_1298 line 02 detail overlay of points 01 – 05    K407_1298 line 02 detail overlay of points 01 – 05 | Point 01    Point 05  Note: Titanium is only present in one of the five points analysed in the line scan. It is therefore omitted from the corrected accumulated spectrum where another point is copied instead to make five points. |
| --- | --- |

### K407_1298 across

| K407_1298 across accumulated spectrum | Point 01    Point 54 |
| --- | --- |
| Image of line scan area from point 01 to point 54 (illuminated laser focus spots are visible in the middle of the cross-hairs on the first and last points) | |
| Linescan results overview | |
| Linescan results detail view | |

### K407_1298 line 03

| K407_1298 line 03 accumulated spectrum    K407_1298 line 03 detail overlay of points 01- 12    K407_1298 line 03 detail overlay of points 01- 12 | Point 01    Point 12  Note: No Cu is not detected in the individual points of analyses. The small raise in background noticed in the accumulated spectrum is most likely an artefact. A corrected accumulated spectrum of just five points was also calculated. |
| --- | --- |

### K407_1298 line 04

| K407_1298 line 04 accumulated spectrum    K407_1298 line 04 detail overlay of points 01 - 07 | Point 01    Point 07 |
| --- | --- |

### K407_1298 line 05

| K407_1298 line 05 accumulated spectrum    K407_1298 line 05 detail overlay of points 01 - 05    K407_1298 line 05 detail overlay of points 01 - 05 | Point 01    Point 05 |
| --- | --- |

### K407_1298 paper

| K407_1298 paper accumulated spectrum    K407_1298 paper detail overlay of points 01 – 05 | Point 01    Point 05 |
| --- | --- |

## K408_1556 page 2, fair condition, Christina (?) and secretary (?), 1685

|  | **Analysis area** | **Comment** | **Instrument settings** |
| --- | --- | --- | --- |
|  | line01 | ink thickness 2, secretary (?) | 5 points, 10 s live time, 0.19 mm spot distance, 0.76 mm length |
|  | line02 | ink thickness 2, sectretary (?) | 5 points, 10 s live time, 0.19 mm spot distance, 0.76 mm length |
|  | line03 | ink thickness 1, secretary (?) | 5 points, 10 s live time, 0.15 mm spot distance, 0.60 mm length |
|  | line04 | ink thickness 2, Christina (?) | 5 points, 10 s live time, 0.15 mm spot distance, 0.60 mm length |
|  | Line05 | ink thickness 2, Christina (?) | 5 points, 10 s live time, 0.19 mm spot distance, 0.76 mm length |
|  | Line06 | ink thickness 2, Christina (?) | 5 points, 10 s live time, 0.17 mm spot distance, 0.68 mm length |
|  | paper |  | 5 points, 10 s live time, 0.25 mm spot distance, 1 mm length |
|  | map | In area written by Secretary(?) with inserted note by Christina(?) | 5600 points, 3 s live time, 0.09 mm spot distance, 5 mm x 8.9 mm area, total scan time 14:00 h |

### K408_1556 page 2 line 01

| K408_1556 page 2 line 01 accumulated spectrum    K408_1556 page 2 line 01 detail overlay of points 01 – 05    K408_1556 page 2 line 01 detail overlay of points 01 – 05 | Point 01    Point 05 |
| --- | --- |

### K408_1556 page 2 line 02

| K408_1556 page 2 line 02 accumulated spectrum    K408_1556 page 2 line 02 detail overlay of points 01 – 05    K408_1556 page 2 line 02 detail overlay of points 01 – 05 | Point 01    Point 05 |
| --- | --- |

### K408_1556 page 2 line 03

| K408_1556 page 2 line 03 accumulated spectrum    K408_1556 page 2 line 03 detail overlay of points 01 – 05 | Point 01    Point 05  Note: Mn and Cu were just noticable at background level in the accumulated spectrum but were not detected in the individual spectra. |
| --- | --- |

### K408_1556 page 2 line 04

| K408_1556 page 2 line 04 accumulated spectrum    K408_1556 page 2 line 04 detail overlay of points 01 – 05    K408_1556 page 2 line 04 detail overlay of points 01 – 05 | Point 01    Point 05  Note:  The small Zn peak observed in the accumulated spectrum was not detected in the individual spectra.  Detector dead time was low at 3.7%. |
| --- | --- |

### K408_1556 page 2 line 05

| K408_1556 page 2 line 05 accumulated spectrum    K408_1556 page 2 line 05 detail overlay of points 01 – 05 | Point 01    Point 05  Note:  The small Mn peak observed in the accumulated spectrum was not detected in the individual spectra. |
| --- | --- |

### K408_1556 page 2 line 06

| K408_1556 page 2 line 06 accumulated spectrum    K408_1556 page 2 line 06 detail overlay of points 01 – 05 | Point 01    Point 05 |
| --- | --- |

### K408_1556 page 2 paper

| K408_1556 page 2 paper accumulated spectrum    K408_1556 page 2 paper detail overlay of points 01 – 05 | Point 01    Point 05 |
| --- | --- |

### K408_1556 page 2 map

| Secretary’s(?) writing and inserted word by Christina(?) with the XRF map area marked in red  K408_1556 map accumulated spectrum    K408_1556 page 2 XRF map area with overlay of images from the map corner points showing the illuminated focus spots. | Point 01    Point 100    Point 5501    Point 5600 |
| --- | --- |
| K408_1556 page 2 XRF mapping results with separate images for the detected elements K, Ca, Mn, Fe, Cu and Pb | |

## K409_1608, good condition, Christina, Rome, 1687

|  | Analysis area | Comment | Instrument settings |
| --- | --- | --- | --- |
|  | line01 | ink thickness 3 | 8 points, 10 s live time, 0.14 mm spot distance, 0.98 mm length |
|  | line02 | ink thickness 2 | Data error; no XRF spectra saved |
|  | line03 | ink thickness 1 | 5 points, 10 s live time, 0.25 mm spot distance, 1 mm length |
|  | line04 | ink thickness 1 | 5 points, 10 s live time, 0.2 mm spot distance, 0.8 mm length |
|  | line05 | ink thickness 3 | 5 points, 10 s live time, 0.13 mm spot distance, 0.54 mm length |
|  | across | ink thickness 3 | 40 points, 10 s live time, 0.04 mm spot distance, 1.56 mm length |
|  | paper | on folded back page of letter circa 1.5 cm in from lower right corner | 5 points, 10 s live time, 0.19 mm spot distance, 0.76 mm length |

### K409_1608 line 01

| K409_1608 line01 accumulated spectrum    K409_1608 line01 detail overlay of points 01-08 | point 01    point 08  Note:  A corrected accumulated spectrum of just five points was also calculated. |
| --- | --- |

### K409_1608 line 03

| K409_1608 line03 accumulated spectrum    K409_1608 line03 detail overlay of points 01-05 | Point 01    Point 05 |
| --- | --- |

### K409_1608 line 04

| K409_1608 line04 accumulated spectrum    K409_1608 line04 detail overlay of points 01-05 | Point 01    Point 05 |
| --- | --- |

### K409_1608 line 05

| K409_1608 line05 accumulated spectrum    K409_1608 line05 detail overlay of points 01-05 | Point 01    Point 05 |
| --- | --- |

### K409_1608 across

|  | | Point 01 Point 40 |
| --- | --- | --- |
| Image of line scan area from point 01 to point 40 (laser pointers are visible in the middle of the cross-hairs) | | |
|  | Linescan results overview | |
|  | Linescan results detail | |

### K409_1608 paper

| K409_1608 paper accumulated spectrum    K409_1608 paper detail overlay of points 01-05 | Point 01    Point 05 |
| --- | --- |

## K412_1902, Adami Aug 1665, Stockholm, good condition

|  | Analysis area | Comment | Instrument settings |
| --- | --- | --- | --- |
|  | Line 01 | Ink thickness, 2 | 5 points, 10 s live time,  0.20 mm spot distance, 0.80 mm length |
|  | Line 02 | Ink thickness, 2 | 4 points, 10 s live time,  0.20 mm spot distance, 0.60 mm length |
|  | Line 03 | Ink thickness, 2 | 5 points, 10 s live time,  0.18 mm spot distance, 0.72 mm length |
|  | Line 04 | Ink thickness, 3 | 5 points, 10 s live time,  0.18 mm spot distance, 0.72mm length |
|  | Across | Ink thickness, 3 and halo | 40 points, 10 s live time, 0,05 spot distance, 1,95 mm length Total mapping time: 10 min |
|  | Paper |  | 3 points, 10 s live time,  0.39 mm spot distance, 0.78mm length |
|  |  |  |  |

### K412 1902 line 01

| K412_1902 line 01 accumulated spectrum    K412_1902 line 01 detail overlay of points 01 – 05 | Point 01    Point 05 |
| --- | --- |

### K412 1902 line 02

| K412_1902 line 02 accumulated spectrum    K412_1902 line 02 detail overlay of points 01 – 04 | Point 01    Point 04  Note:  The linescan contained only four points. A corrected accumulated spectrum was also calculated by copying one of the points to make five points for better comparison with all other accumulated spectra. |
| --- | --- |

### K412 1902 line 03

| K412_1902 line 03 accumulated spectrum    K412_1902 line 03 detail overlay of points 01 – 05 | Point 01    Point 05 |
| --- | --- |

### K412 1902 line 04

| K412_1902 line 04 accumulated spectrum    K412_1902 line 04 detail overlay of points 01 – 05 | Point 01    Point 05 |
| --- | --- |

### K412 1902 across

| K412 1902 across accumulated spectrum | Point 01    Point 40 |
| --- | --- |
| Image of line scan area from point 01 to point 40 (laser pointers are visible in the middle of the cross-hairs) | |
|  | Linescan results overview |
|  | Linescan results detail |

### K412 1902 paper

| K412_1902 paper accumulated spectrum    K412_1902 paper detail overlay of points 01 – 03 | Point 01    Point 03  Note:  The linescan contained only three points. A corrected accumulated spectrum was also calculated by copying two of the points to make five points for better comparison with all other accumulated spectra. |
| --- | --- |

## K415_2098, fair condition, Azzolino, 1661

|  | Analysis area | Comment | Instrument settings |
| --- | --- | --- | --- |
|  | line01 | ink thickness 2 | 5 points, 10 s live time, 0.27 mm spot distance, 1.08 mm length |
|  | line02 | ink thickness 2 | 3 points, 10 s live time, 0.27 mm spot distance, 0.54 mm length |
|  | line03 | ink thickness 3 | 5 points, 10 s live time, 0.14 mm spot distance, 0.56 mm length |
|  | line04 | ink thickness 1 | 5 points, 10 s live time, 0.19 mm spot distance, 0.76 mm length |
|  | paper |  | 5 points, 10 s live time, 0.19 mm spot distance, 0.76 mm length |
|  |  |  |  |
|  |  |  |  |

### K415_2098 line 01

| K415_2098 line 01 accumulated spectrum    K415_2098 line 01 detail overlay of points 01 – 05    K415_2098 line 01 detail overlay of points 01 – 05 | Point 01    Point 05 |
| --- | --- |

### K415_2098 line 02

| K415_2098 line 02 accumulated spectrum    K415_2098 line 02 detail overlay of points 01 – 05 | Point 01    Point 05  Note:  The linescan contained only three points. A corrected accumulated spectrum was also calculated by copying two of the points to make five points for better comparison with all other accumulated spectra. |
| --- | --- |

### K415_2098 line 03

| K415_2098 line 03 accumulated spectrum    K415_2098 line 03 detail overlay of points 01 – 05    K415_2098 line 03 detail overlay of points 01 – 05 | Point 01    Point 05 |
| --- | --- |

### K415_2098 line 04

| K415_2098 line 04 accumulated spectrum    K415_2098 line 04 detail overlay of points 01 – 05    K415_2098 line 04 detail overlay of points 01 – 05 | Point 01    Point 05  Note:  Zn and Mn were not detected in the individual spectra although the accumulated spectrum does show a small peak near background level for Mn. |
| --- | --- |

### K415_2098 paper

| K415_2098 paper accumulated spectrum    K415_2098 paper detail overlay of points 01 – 05 | Point 01    Point 05  Note:  Cu was not detected in the individual spectra and the small peak in the accumulated spectrum has a poor background fit that would lead to overestimation of peak area.  Detector dead time was low at 1.8%. |
| --- | --- |

## K415_2182 fair condition, Azzolino, 1671

|  | Analysis area | Comment | Instrument settings |
| --- | --- | --- | --- |
|  | Line 01 | ink thickness 2 | 5 points, 10 s live time, 0.27 mm spot distance, 1.08 mm length |
|  | Line 02 | ink thickness 3 | 3 points, 10 s live time, 0.27 mm spot distance, 0.54 mm length |
|  | Line 03 | ink thickness 2, halo | 5 points, 10 s live time, 0.18 mm  spot distance, 0.72 mm length |
|  | Line 04 | ink thickness 2 | 5 points, 10 s live time, 0.21 mm  spot distance, 0.84 mm length |
|  | Line 05 | ink thickness 1 (?)  Line position not marked on image | 5 points, 10 s live time, 0.19 mm  spot distance, 0.76 mm length |
|  | across | ink thickness 2, halo, paper | 54 points, 10 s live time, 0.08 mm spot distance, 4.24 mm |
|  | paper |  | 5 points, 10 s live time, 0.19 mm  spot distance, 0.76 mm length |

### K415_2182 line 01

| K415_2182 line 01 accumulated spectrum    K415_2182 line 01 detail overlay of points 01 – 05 | Point 01    Point 05 |
| --- | --- |

### K415_2182 line 02

| K415_2182 line 02 accumulated spectrum    K415_2182 line 02 detail overlay of points 01 – 03    K415_2182 line 02 detail overlay of points 01 – 03 | Point 01    Point 03  Note:  The linescan contained only three points. A corrected accumulated spectrum was also calculated by copying two of the points to make five points for better comparison with all other accumulated spectra.  Detector dead time was low at 4.0%. |
| --- | --- |

### vK415_2182 line 03

| K415_2182 line 03 accumulated spectrum    K415_2182 line 03 detail overlay of points 01 – 05    K415_2182 line 03 detail overlay of points 01 – 05 | Point 01    Point 05  Note:  A small Zn peak was observable in the accumulated spectrum but not in the individual spectra. |
| --- | --- |

### K415_2182 across

| K415_2182 across    Image of line scan area from point 01 to point 54 (with laser focus spots in the cross-hairs)  K415_2182 across linescan overview    K415_2182 across linescan detail | Point 01    Point 54 |
| --- | --- |

### K415_2182 line 04

| K415_2182 line 04 accumulated spectrum    K415_2182 line 04 detail overlay of points 01 – 05    K415_2182 line 04 detail overlay of points 01 – 05 | Point 01    Point 05 |
| --- | --- |

### K415_2182 line 05

| K415_2182 line 05 accumulated spectrum    K415_2182 line 05 detail overlay of points 01 – 05 | Point 01    Point 05 |
| --- | --- |

### K415_2182 paper

| K415_2182 paper accumulated spectrum    K415_2182 paper detail overlay of points 01 – 05 | Point 01    Point 05  Note:  The XRF spectrum of one of the five points in the line scan showed the presence of lead (Pb). |
| --- | --- |

## K419_2574, Texeira, 6 Aug 1664, Hamburg, good condition

|  | Analysis area | Comment | Instrument settings |
| --- | --- | --- | --- |
|  | line01 | ink thickness 1 | 5 points, 10 s live time, 0.19 mm  spot distance, 0.76 mm length |
|  | line02 | ink thickness 2 | 5 points, 10 s live time, 0.24 mm  spot distance, 0.96 mm length |
|  | line03 | ink thickness 2 | 5 points, 10 s live time, 0.24 mm  spot distance, 0.96 mm length |
|  | line04 | ink thickness 1 | 5 points, 10 s live time, 0.20 mm  spot distance, 0.80 mm length |
|  | Line05 | Page 3, ink thickness 2 | 5 points, 10 s live time, 0.16 mm  spot distance, 0.64 mm length |
|  | paper |  | 5 points, 10 s live time |

### K419_2574 line 01

| K419_2574 line 01 accumulated spectrum    K419_2574 line 01 detail overlay of points 01 – 05    K419_2574 line 01 detail overlay of points 01 – 05 | Point 01    Point 05 |
| --- | --- |

### K419_2574 line 02

| K419_2574 line 02 accumulated spectrum    K419_2574 line 02 detail overlay of points 01 – 05 | Point 01    Point 05 |
| --- | --- |

### K419_2574 line 03

| K419_2574 line 03 accumulated spectrum    K419_2574 line 03 detail overlay of points 01 – 05 | Point 01    Point 05 |
| --- | --- |

### K419_2574 line 04

| K419_2574 line 04 accumulated spectrum    K419_2574 line 04 detail overlay of points 01 – 05 | Point 01    Point 05 |
| --- | --- |

### K419_2574 line 05

| K419_2574 line 05 accumulated spectrum    K419_2574 line 05 detail overlay of points 01 – 05 | Point 01    Point 05 |
| --- | --- |

### K419_2574 paper

| K419_2574 paper accumulated spectrum    K419_2574 paper detail overlay of points 01 – 05 | Point 01    Point 05 |
| --- | --- |

## K420_2868, Texeira June 1689, Hamburg, good condition, deposits present

|  | Analysis area | Comment | Instrument settings |
| --- | --- | --- | --- |
|  | line01 | ink thickness 2 | 5 points, 10 s live time, 0.20 mm  spot distance, 0.80 mm length |
|  | line02 | ink thickness 2 | 5 points, 10 s live time, 0.19 mm  spot distance, 0.76 mm length |
|  | line03 | ink thickness 2 | 5 points, 10 s live time, 0.19 mm  spot distance, 0.76 mm length |
|  | line04 | Page 2, ink thickness 2 | 5 points, 10 s live time, 0.22 mm  spot distance, 0.88 mm length |
|  | Line05 | Page 2, ink thickness 3 | No data |
|  | paper |  | 5 points, 10 s live time, 0.17 mm  spot distance, 0.68 mm length |

### K420_2868 line 01

| K420_2868 line 01 accumulated spectrum    K420_2868 line 01 detail overlay of points 01 – 05 | Point 01    Point 05  Detector dead time was low at 4.1%. |
| --- | --- |

### K420_2868 line 02

| K420_2868 line 02 accumulated spectrum    K420_2868 line 02 detail overlay of points 01 – 05 | Point 01    Point 05 |
| --- | --- |

### K420_2868 line 03

| K420_2868 line 03 accumulated spectrum    K420_2868 line 03 detail overlay of points 01 – 05 | Point 01    Point 05 |
| --- | --- |

### K420_2868 line 04

| K420_2868 line 04 accumulated spectrum  K420_2868 line 04 corrected accumulated spectrum without point 2    K420_2868 line 04 detail overlay of points 01 – 05  K420_2868 line 04 detail overlay of points 01 – 05 | Point 01    Point 05  Note:  Only one point showed the presence of Si, Zn, Pb and higher than usual levels of K, Ca, Fe and Cu. A corrected accumulated spectrum was calculated without that point. |
| --- | --- |

### K420_2868 paper

| K420_2868 paper accumulated spectrum    K420_2868 paper detail overlay of points 01 – 05 | Point 01    Point 05 |
| --- | --- |

## K421_3064, “Comptes”, water damage, fair condition

|  | Analysis area | Comment | Instrument settings |
| --- | --- | --- | --- |
|  | Line 01 | ink thickness 1 | 5 points, 10 s live time, 0.22 mm spot distance, 0.88 mm length |
|  | Line 02 | ink thickness 2 | 5 points, 10 s live time, 0.22 mm spot distance, 0.88 mm length |
|  | Line 03 | ink thickness 2 | 5 points, 10 s live time, 0.22 mm spot distance, 0.88 mm length |
|  | Line 04 | ink thickness 1, water damage | 5 points, 10 s live time, 0.23 mm spot distance, 0.92 mm length |
|  | Line 05 | ink thickness 2, water damage | 5 points, 10 s live time, 0.29 mm spot distance, 1.16 mm length |
|  | Line 06 | ink thickness 1, water damage | 5 points, 10 s live time, 0.15 mm spot distance, 0.60 mm length |
|  | Across | ink thickness 2, water damage | 47 points, 10 s live time, 0.04 mm spot distance, 1.84 mm |
|  | Paper |  | 5 points, 10 s live time, 0.24 mm spot distance, 0.96 mm length |

### K421_3064 line 01

| K421_3064 line 01 accumulated spectrum    K421_3064 line 01 detail overlay of points 01 – 05 | Point 01    Point 05 |
| --- | --- |

### K421_3064 line 02

| K421_3064 line 02 accumulated spectrum    K421_3064 line 02 detail overlay of points 01 – 05 | Point 01    Point 05 |
| --- | --- |

### K421_3064 line 03

| K421_3064 line 03 accumulated spectrum    K421_3064 line 03 detail overlay of points 01 – 05    K421_3064 line 03 detail overlay of points 01 – 05 | Point 01    Point 05 |
| --- | --- |

### K421_3064 line 04

| K421_3064 line 04 accumulated spectrum    K421_3064 line 04 detail overlay of points 01 – 05 | Point 01    Point 05 |
| --- | --- |

### K421_3064 line 05

| K421_3064 line 05 accumulated spectrum    K421_3064 line 05 detail overlay of points 01 – 05 | Point 01    Point 05 |
| --- | --- |

### K421_3064 line 06

| K421_3064 line 06 accumulated spectrum    K421_3064 line 06 detail overlay of points 01 – 05 | Point 01    Point 05 |
| --- | --- |

### K421_3064 across

| K421_3064 across accumulated spectrum    Image of linescan area with the two endpoints marked by the laser focus points | Point 01    Point 47 |
| --- | --- |
| Image of linescan area with the two endpoints marked by the laser focus points | |
|  | Linescan results overview |
|  | Linescan results detail |

### K421_3064 paper

| K421_3064 paper accumulated spectrum    K421_3064 paper detail overlay of points 01 – 05 | Point 01    Point 05 |
| --- | --- |

## K422_3078, fair condition, Christina signature, Rome, 1669

|  | Analysis area | Comment | Instrument settings |
| --- | --- | --- | --- |
|  | line01 | ink thickness 3, Christina signature in poor condition | 5 points, 10 s live time, 0.21 mm spot distance, 0.84 mm length |
|  | line02 | ink thickness 3, Christina signature in poor condition | 5 points, 10 s live time, 0.21 mm spot distance, 0.84 mm length |
|  | line03 | ink thickness 2 | 5 points, 10 s live time, 0.21 mm spot distance, 0.84 mm length |
|  | line04 | ink thickness 2 | 5 points, 10 s live time, 0.18 mm spot distance, 0.72 mm length |
|  | paper |  | No data |
|  | map | Christina signature, cracks, water damage | 5472 points, 3 s live time, 0.16 mm spot distance, 23 mm x 6 mm area, total scan time 13:40 h |

### K422_3078 line 01

| K422_3078 line 01 accumulated spectrum    K422_3078 line 01 detail overlay of points 01 - 05 | Point 01    Point 05 |
| --- | --- |

### K422_3078 line 02

| K422_3078 line 02 accumulated spectrum    K422_3078 line 02 detail overlay of points 01 – 05 | Point 01    Point 05 |
| --- | --- |

### K422_3078 line 03

| K422_3078 line 03 accumulated spectrum    K422_3078 line 03 detail overlay of points 01 – 05 | Point 01    Point 05 |
| --- | --- |

### K422_3078 line 04

| K422_3078 line 04 accumulated spectrum    K422_3078 line 04 detail overlay of points 01 – 05 | Point 01    Point 05 |
| --- | --- |

### K422_3078 map

| Christina’s signature with the XRF map area marked in red |
| --- |
| K422_3078 accumulated spectrum |
| Detail of Christina’s signature with the XRF map area marked in red and overlay of images from the map corner points showing the illuminated focus spots. |
| K422_3078 XRF mapping results with separate images for the detected elements K, Ca, Mn, Fe and Cu. |
|  |
|  |
|  |
|  |

## K422_3190, Mémoire Christina and secretary, strong fluorescence, good condition

|  | Analysis area | Comment | Instrument settings |
| --- | --- | --- | --- |
|  | Line 01 | Ink thickness 2 | 5 points, 10 s live time, 0.14 mm spot distance, 0.56 mm length |
|  | Line 02 | Ink thickness 2 | 5 points, 10 s live time, 0.24 mm spot distance, 0.96 mm length |
|  | Line 03 | Ink thickness 2 | 5 points, 10 s live time, 0.13 mm spot distance, 0.52 mm length |
|  | Line 04 |  | No data |
|  | Line 05 | Ink thickness 1 | 5 points, 10 s live time, 0.19 mm spot distance, 0.76 mm length |
|  | Line 06 | Ink thickness 1 | 5 points, 10 s live time, 0.25 mm spot distance, 1.00 mm length |
|  | Line 07 Across | Across fluorescence, cold yellow from adjacent text | 55 points, 0.06 spot distance, 3.24 mm length, total scanning time: 14 min |
|  | Paper |  | No data |
|  | Map | In area with fluorescence of warm yellow (from the back) and cold yellow (from contact with adjacent text) | 5183 points, 0.1 mm spot distance, 3 s live time, 7.0 mm x 7.2 mm map area, total scanning time: 13 hours |

### K422_3190 line 01

| K422 3190 line 01 accumulated spectrum    K422 3190 line 01 detail overlay of points 01 – 05 | Point 01    Point 05 |
| --- | --- |

### K422_3190 line 02

| K422 3190 line 02 accumulated spectrum    K422 3190 line 02 detail overlay of points 01 – 05 | Point 01    Point 05 |
| --- | --- |

### K422_3190 line 03

| K422 3190 line 03 accumulated spectrum    K422 3190 line 03 detail overlay of points 01 – 05 | Point 01    Point 05  Note:  The last point of the scan contained significantly higher copper and zinc peaks. This point was placed on the line that strikes through and is therefore not related to the other four points of the scan. A corrected accumulated spectrum was calculated without that last point and instead one of the other points was copied to make up for the missing spectrum. |
| --- | --- |

### K422_3190 line 05

| K422 3190 line 05 accumulated spectrum    K422 3190 line 05 detail overlay of points 01 – 05 | Point 01    Point 05 |
| --- | --- |

### K422_3190 line 06

| K422 3190 line 06 accumulated spectrum    K422 3190 line 06 detail overlay of points 01 – 05 | Point 01    Point 05 |
| --- | --- |

### K422_3190 line 07 across

| K422_3190 across accumulated spectrum    Image of linescan area with the two endpoints marked by the laser focus points | Point 01    Point 55 |
| --- | --- |
| Image of linescan area with the two endpoints marked by the laser focus points | |
|  | Linescan results overview |
|  | Linescan results detail |

### K422_3190 map

| Approximate XRF map marked in red in area with fluorescence of warm yellow (from the back) and cold yellow (from contact with adjacent text) | Point 01    Point 73    Point 5111    Point 5183 |
| --- | --- |
| K422_3190 accumulated spectrum |  |
| Detail of XRF map area marked in red and overlay of images from the map corner points showing the illuminated focus spots. | |

| K422_3190 XRF mapping results  Note: a small copper containing grain in the mapping area was cut from the results as it was obscuring the data. The deleted pixels can be seen as a small black “hole” in some of the maps. | |
| --- | --- |
|  |  |
|  |  |
|  |  |
|  |  |
|  |  |
|  |  |

## K423_3234, fair condition, Azzolino, Rome, 1669

|  | Analysis area | Comment | Instrument settings |
| --- | --- | --- | --- |
|  | line01 | ink thickness 2 | 5 points, 10 s live time, 0.28 mm spot distance, 1.12 mm length |
|  | line02 | ink thickness 1 | 5 points, 10 s live time, 0.21 mm spot distance, 0.84 mm length |
|  | line03 | ink thickness 3 | 5 points, 10 s live time, 0.18 mm spot distance, 0.72 mm length |
|  | line04 | ink thickness 2 | 5 points, 10 s live time, 0.23 mm spot distance, 0.92 mm length |
|  | paper |  | 5 points, 10 s live time, 0.29 mm spot distance, 1.16 mm length |
|  |  |  |  |
|  |  |  |  |

### K423_3234 line 01

| K423_3234 line 01 accumulated spectrum    K423_3234 line 01 detail overlay of points 01 – 05    K423_3234 line 01 detail overlay of points 01 – 05 | Point 01    Point 05  Note: Zn was not noticable in the accumulated spectrum but possibly in individual spectra |
| --- | --- |

### K423_3234 line 02

| K423_3234 line 02 accumulated spectrum    K423_3234 line 02 detail overlay of points 01 – 05 | Point 01    Point 05 |
| --- | --- |

### K423_3234 line 03

| K423_3234 line 03 accumulated spectrum    K423_3234 line 03 detail overlay of points 01 – 05    K423_3234 line 03 detail overlay of points 01 – 05 | Point 01    Point 05  Note: Cu and Zn were not detected while Ni was possibly present just above background level. |
| --- | --- |

### K423_3234 line 04

| K423_3234 line 04 accumulated spectrum    K423_3234 line 04 detail overlay of points 01 – 05 | Point 01    Point 05 |
| --- | --- |

### K423_3234 paper

| K423_3234 paper accumulated spectrum    K423_3234 paper detail overlay of points 01 – 05 | Point 01    Point 05  Note:  Mn was possibly just below the limit of detection. A small Mn peak appears in the accumulated spectrum but it is not evident in the individual spectra. |
| --- | --- |

## K429_3767, fair condition, Christina, Alchemy drawing

|  | Analysis area | Comment | Instrument settings |
| --- | --- | --- | --- |
|  | line01 | ink thickness 2 | 5 points, 10 s live time, 0.17 mm spot distance, 0.68 length |
|  | line02 | ink thickness 2 | 5 points, 10 s live time, 0.14 mm spot distance, 0.56 length |
|  | line03 | ink thickness 2 | 5 points, 10 s live time, 0.23 mm spot distance, 0.92 length |
|  | line04 | ink thickness 2, drawing | 5 points, 10 s live time, 0.23 mm spot distance, 0.92 length |
|  | line05 | brown wash | 5 points, 10 s live time, 0.17 mm spot distance, 0.68 length |
|  | across | ink thickness, paper, ink, wash | 52 points, 10 s live time, 0.14 mm spot distance, 7 mm length |
|  | paper |  | 5 points, 10 s live time, 0.14 mm spot distance, 0.54 mm length |

### K429_3767 line 01

| K429_3767 line 01 accumulated spectrum    K429_3767 line 01 detail overlay of points 01 – 05 | Point 01    Point 05 |
| --- | --- |

### K429_3767 line 02

| K429_3767 line 02 accumulated spectrum    K429_3767 line 02 detail overlay of points 01 – 05 | Point 01    Point 05 |
| --- | --- |

### K429_3767 line 03

| K429_3767 line 03 accumulated spectrum    K429_3767 line 03 detail overlay of points 01 – 05    K429_3767 line 03 detail overlay of points 01 – 05 | Point 01    Point 05 |
| --- | --- |

### K429_3767 line 04

| K429_3767 line 04 accumulated spectrum    K429_3767 line 04 detail overlay of points 01 – 05 | Point 01    Point 05 |
| --- | --- |

### K429_3767 line 05

| K429_3767 line 05 accumulated spectrum    K429_3767 line 05 detail overlay of points 01 – 05 | Point 01    Point 05  Note:  The ink-wash is very thin, hence the very low Cu signal observed in the other line scans on this object may be lost in the background here. |
| --- | --- |

### K409_3767 paper

| K429_3767 paper accumulated spectrum    K429_3767 paper detail overlay of points 01 – 05 | Point 01    Point 05  Note:  All five spectra have very low counts; ca. 5 to 8 times lower than usual. No instrument settings were changed between this analysis and earlier lines on the same paper. However, the spectral parameters show that the detector deadtime was less than 1,4% when it is normally around 5-9%. |
| --- | --- |

## K429_3800, Christina (?), fair condition

|  | Analysis area | Comment | Instrument settings |
| --- | --- | --- | --- |
|  | Line 01 | ink thickness 3 | 5 points, 10 s live time, 0.14 mm spot distance, 0.56 mm length |
|  | Line 02 | halo | 5 points, 10 s live time, 0.14 mm spot distance, 0.56 mm length |
|  | Line 03 | ink thickness 2 | 5 points, 10 s live time, 0.25 mm spot distance, 1.00 mm length |
|  | Line 04 | ink thickness 3 | 5 points, 10 s live time, 0.25 mm spot distance, 1 mm length |
|  | across | ink thickness, 3, halo and outer halo | 51 points, 10 s live time, 0.09 mm spot distance, 4.5 mm length |
|  | Line 05 | halo | 5 points, 10 s live time, 0.14 mm spot distance, 0.56 mm length |
|  | Line 06 | outer halo | 5 points, 10 s live time, 0.17 mm spot distance, 0.68 mm length |
|  | paper |  | 4 points, 10 s live time, 10 s live time, 0.17 mm spot distance, 0.51 mm length |

### K429_3800 line 01

| K429_3800 line 01 accumulated spectrum    K429_3800 line 01 detail overlay of points 01 – 05    K429_3800 line 01 detail overlay of points 01 – 05 | Point 01    Point 05 |
| --- | --- |

### K429_3800 line 02

| K429_3800 line 02 accumulated spectrum    K429_3800 line 02 detail overlay of points 01 – 05 | Point 01    Point 05 |
| --- | --- |

### K429_3800 line 03

| K429_3800 line 03 accumulated spectrum    K429_3800 line 03 detail overlay of points 01 – 05    K429_3800 line 03 detail overlay of points 01 – 05 | Point 01    Point 05 |
| --- | --- |

### K429_3800 line 04

| K429_3800 line 04 accumulated spectrum    K429_3800 line 04 detail overlay of points 01 – 05    K429_3800 line 04 detail overlay of points 01 – 05 | Point 01    Point 05  Note: A peak for zinc is not discernible in the individual spectra. |
| --- | --- |

### K429_3800 line across

| K429_3800 line across accumulated spectrum  Image of linescan area with the two endpoints marked by the laser focus points | Point 01    Point 51 |
| --- | --- |
|  | Linescan results overview |
|  | Linescan resultsdetail |

### K429_3800 line 05

| K429_3800 line 05 accumulated spectrum    K429_3800 line 05 detail overlay of points 01 – 05 | Point 01    Point 05 |
| --- | --- |

### K429_3800 line 06

| K429_3800 line 06 accumulated spectrum    K429_3800 line 06 detail overlay of points 01 – 05 | Point 01    Point 05 |
| --- | --- |

### K429_3800 line paper

| K429_3800 line paper accumulated spectrum    K429_3800 line paper detail overlay of points 01 – 04 | Point 01    Point 04  Note:  The linescan contained only four points. A corrected accumulated spectrum was also calculated by copying one of the points to make five points for better comparison with all other accumulated spectra. |
| --- | --- |

## K429_3801, Christina (?), fair condition

|  | Analysis area | Comment | Instrument settings |
| --- | --- | --- | --- |
|  | line01 | ink thickness 3 | 5 points, 10 s live time, 0.20 mm spot distance, 0.80 mm length |
|  | line02 | halo | 5 points, 10 s live time, 0.56 mm spot distance, 0.14 mm length |
|  | line03 | ink thickness 2 | 5 points, 10 s live time, 0.21 mm spot distance, 0.84 mm length |
|  | line04 | halo | 5 points, 10 s live time, 0.04 mm spot distance, 0.16 mm length |
|  | line05 | ink thickness 2 | 5 points, 10 s live time, 0.26 mm spot distance, 1.04 mm length |
|  | paper |  | 21 points, 10 s live time, 0.04 mm spot distance, 0.80 mm length |

### K429_3801 line 01

| K429_3801 line 01 accumulated spectrum    K429_3801 line 01 detail overlay of points 01 – 05    K429_3801 line 01 detail overlay of points 01 – 05 | Point 01    Point 05 |
| --- | --- |

### K429_3801 line 02

| K429_3801 line 02 accumulated spectrum    K429_3801 line 02 detail overlay of points 01 – 05 | Point 01    Point 05 |
| --- | --- |

### K429_3801 line 03

| K429_3801 line 03 accumulated spectrum    K429_3801 line 03 detail overlay of points 01 – 05 | Point 01    Point 05 |
| --- | --- |

### K429_3801 line 04

| K429_3801 line 04 accumulated spectrum    K429_3801 line 04 detail overlay of points 01 – 05 | Point 01    Point 05 |
| --- | --- |

### K429_3801 line 05

| K429_3801 line 05 accumulated spectrum    K429_3801 line 05 detail overlay of points 01 – 05 | Point 01    Point 05 |
| --- | --- |

### K429_3801 paper

| K429_3801 paper accumulated spectrum    K429_3801 paper corrected accumulated spectrum    K429_3801 paper detail overlay of points 01 – 05 | Point 01    Point 21  Note:  A corrected accumulated spectrum with only 5 points was also calculated. |
| --- | --- |

## K430_3842 Part 1 fair condition, Christina and secretary

|  | **Analysis area** | **Comment** | **Instrument settings** |
| --- | --- | --- | --- |
|  | line01 | ink thickness 2, Christina | 5 points, 10 s live time, 0.26 mm spot distance, 1.04 mm length |
|  | line02 | ink thickness 3, Christina | 5 points, 10 s live time, 0.26 mm spot distance, 1.04 mm length |
|  | line03 | ink thickness 3, Christina | 5 points, 10 s live time, 0.17 mm spot distance, 0.68 mm length |
|  | line04 | ink thickness 2, secretary | 6 points, 10 s live time, 0.22 mm spot distance, 1.10 length |
|  | Line05 | ink thickness 2, secretary | 6 points, 10 s live time, 0.22 mm spot distance, 1.10 length |
|  | Line06 | ink thickness 2, secretary | 5 points, 10 s live time, 0.22 mm spot distance, 0.88 length |
|  | paper |  | 5 points, 10 s live time, 0.22 mm spot distance, 0.88 length |
|  |  |  |  |

### K430_3842 Part 1 line 01

| K430_3842 Part 1 line 01 accumulated spectrum    K430_3842 Part 1 line 01 detail overlay of points 01 – 05 | Point 01    Point 05 |
| --- | --- |

### K430_3842 Part 1 line 02

| K430_3842 Part 1 line 02 accumulated spectrum    K430_3842 Part 1 line 02 detail overlay of points 01 – 05    K430_3842 Part 1 line 02 detail overlay of points 01 – 05 | Point 01    Point 05  Note:  Ni and Zn appeared in the accumulated spectrum but were almost unobservable in the individual spectra.  Detector dead time was low at 4.1%. |
| --- | --- |

### K430_3842 Part 1 line 03

| K430_3842 Part 1 line 03 accumulated spectrum    K430_3842 Part 1 line 03 detail overlay of points 01 – 05 | Point 01    Point 05 |
| --- | --- |

### K430_3842 Part 1 line 04

| K430_3842 Part 1 line 04 accumulated spectrum    K430_3842 Part 1 line 04 detail overlay of points 01 – 06 | Point 01    Point 06  Note:  The Mn peak was discernible in the accumulated spectrum but not in the individual spectra.  The linescan contains six points. A corrected accumulated spectrum was also calculated from five points only. |
| --- | --- |

### K430_3842 Part 1 line 05

| K430_3842 Part 1 line 05 accumulated spectrum    K430_3842 Part 1 line 05 detail overlay of points 01 – 05    K430_3842 Part 1 line 05 detail overlay of points 01 – 05 | Point 01    Point 06  Note:  The linescan contains six points. A corrected accumulated spectrum was also calculated from five points only. |
| --- | --- |

### K430_3842 Part 1 line 06

| K430_3842 Part 1 line 06 accumulated spectrum    K430_3842 Part 1 line 06 detail overlay of points 01 – 05 | Point 01    Point 05 |
| --- | --- |

### K430_3842 Part 1 paper

| K430_3842 Part 1 paper accumulated spectrum    K430_3842 Part 1 paper detail overlay of points 01 – 05 | Point 01    Point 05 |
| --- | --- |

# Results table

Net peak areas calculated from regions of interest

| **Spectrum** | **Si** | **P** | **S** | **Cl** | **Ar** | **K** | **Ca** | **Mn** | **Fe** | **Ni** | **Cu** | **Zn** | **Hg** | **Pb** | **Compton** | **Mo** |
| --- | --- | --- | --- | --- | --- | --- | --- | --- | --- | --- | --- | --- | --- | --- | --- | --- |
| Accu_Line Del Monte Vol II A1 No 17 line 01 | 44 | 278 | 458 | 191 | 1790 | 28648 | 26219 | 1617 | 143444 | 152 | 2185 | 3627 | 2701 | 2329 | 181703 | 22186 |
| Accu_Line Del Monte Vol II A1 No 17 line 02 | 120 | 165 | 427 | 27 | 1624 | 31940 | 21193 | 1609 | 163023 | -328 | 1774 | 3351 | 2312 | 2086 | 180849 | 21741 |
| Accu_Line Del Monte Vol II A1 No 17 line 03 | 104 | 248 | 398 | 83 | 1679 | 26105 | 25542 | 1409 | 178056 | 38 | 1899 | 3223 | 2988 | 3040 | 199239 | 24354 |
| Accu_Line Del Monte Vol II A1 No 17 line 04 | 149 | 231 | 529 | -45 | 1701 | 34222 | 21279 | 1842 | 264498 | -287 | 1539 | 3970 | 2649 | 2193 | 194402 | 23773 |
| Accu_Line Del Monte Vol II A1 No 17 paper | 61 | 116 | 340 | 1030 | 1838 | 1671 | 19234 | 1022 | 4060 | -84 | 1990 | 2817 | 2777 | 2666 | 203069 | 24203 |
| Accu_Line K394 0014 line 01 | 75 | 101 | 581 | 101 | 1895 | 24381 | 11648 | 3005 | 74351 | -84 | 8022 | 10803 | 1746 | 2037 | 182195 | 22021 |
| Accu_Line K394 0014 line 02 | 203 | 149 | 930 | 43 | 1848 | 64970 | 17534 | 4886 | 212247 | 32 | 21141 | 23393 | 1874 | 1320 | 158623 | 19258 |
| Accu_Line K394 0014 line 03 | 138 | 114 | 390 | 115 | 2146 | 5631 | 10751 | 3051 | 23100 | 134 | 2435 | 3821 | 1179 | 1300 | 93154 | 11055 |
| Accu_Line K394 0014 line 04 | 87 | 158 | 494 | 33 | 1834 | 22820 | 12419 | 3119 | 78561 | 269 | 7935 | 10598 | 2841 | 2286 | 198283 | 24483 |
| Accu_Line K394 0014 line 05 | 145 | 137 | 1187 | 91 | 1738 | 39247 | 15112 | 3828 | 112006 | -201 | 12026 | 17212 | 2794 | 2403 | 202103 | 25054 |
| Accu_Line K394 0014 paper | 94 | 99 | 605 | 462 | 2387 | 3366 | 8212 | 2667 | 6532 | 133 | 411 | 523 | 124 | 231 | 9372 | 1472 |
| Accu_Line K396 266 line 01 | 245 | 285 | 652 | 61 | 1823 | 54902 | 30843 | 1401 | 215054 | 560 | 2191 | 1405 | 477 | 1191 | 84518 | 10095 |
| Accu_Line K396 266 line 02 | 210 | 207 | 480 | 80 | 1648 | 26341 | 22673 | 1021 | 141336 | 50 | 2290 | 2800 | 2688 | 2491 | 199347 | 24167 |
| Accu_Line K396 266 line 03 | 315 | 677 | 688 | 262 | 1622 | 26653 | 80187 | 1288 | 189122 | 248 | 2852 | 2074 | 2276 | 2911 | 173724 | 21392 |
| Accu_Line K396 266 line 04 | 266 | 723 | 457 | 369 | 1721 | 17681 | 88486 | 1007 | 208373 | 312 | 2809 | 2108 | 2213 | 1571 | 143386 | 17066 |
| Accu_Line K396 266 line 05 corrected | 262 | 214 | 451 | 0 | 1542 | 82184 | 25269 | 2056 | 305356 | 1413 | 5599 | 1703 | 869 | 560 | 85725 | 11389 |
| Accu_Line K396 266 paper | 152 | 159 | 309 | 1330 | 1849 | 2322 | 21025 | 736 | 4699 | 126 | 1676 | 2954 | 2058 | 1532 | 140497 | 17065 |
| Accu_Line K397 364 line 01 | 217 | 318 | 611 | 2 | 1651 | 32712 | 27788 | 3953 | 177338 | 245 | 1733 | 5134 | 2229 | 1649 | 156648 | 18870 |
| Accu_Line K397 364 line 02 | 200 | 370 | 353 | 110 | 1647 | 19578 | 31612 | 3499 | 127651 | 228 | 1855 | 3897 | 1816 | 2098 | 165919 | 20225 |
| Accu_Line K397 364 line 03 | 218 | 248 | 451 | 74 | 1635 | 42317 | 24537 | 4172 | 187908 | -202 | 1789 | 5107 | 2829 | 2412 | 190518 | 23228 |
| Accu_Line K397 364 line 04 | 149 | 429 | 661 | 178 | 1792 | 8366 | 44378 | 2908 | 109701 | 315 | 1583 | 3579 | 2926 | 2137 | 195547 | 24085 |
| Accu_Line K397 364 line 05 | 139 | 416 | 636 | 159 | 1753 | 8040 | 43835 | 2400 | 108170 | 240 | 988 | 2615 | 1978 | 950 | 175813 | 23436 |
| Accu_Line K397 364 paper | 148 | 93 | 653 | 442 | 1783 | 4084 | 10751 | 2327 | 6312 | 537 | 2347 | 2767 | 2459 | 2129 | 179428 | 21692 |
| Accu_Line K399 470 line 01 | 80 | 203 | 918 | 35 | 1748 | 20580 | 18753 | 1330 | 95848 | 183 | 1651 | 2163 | 1882 | 1773 | 130593 | 15628 |
| Accu_Line K399 470 line 02 | 67 | 161 | 1189 | 71 | 2094 | 18048 | 13624 | 811 | 97921 | 109 | 1652 | 2124 | 740 | 352 | 78493 | 8987 |
| Accu_Line K399 470 line 03 | 62 | 117 | 1285 | 65 | 1868 | 13231 | 12974 | 567 | 63477 | 32 | 1555 | 2792 | 2489 | 1775 | 159942 | 19355 |
| Accu_Line K399 470 line 04 | 198 | 267 | 1143 | 33 | 1728 | 24313 | 18039 | 1431 | 118485 | 46 | 2188 | 2782 | 1515 | 1556 | 135067 | 16136 |
| Accu_Line K399 470 line 05 | 108 | 126 | 1236 | 64 | 1769 | 18179 | 16431 | 1070 | 75423 | -19 | 2266 | 2715 | 2180 | 1924 | 146139 | 17263 |
| Accu_Line K399 470 paper | 152 | 58 | 1449 | 83 | 1920 | 4836 | 8198 | 571 | 5265 | 498 | 2386 | 2642 | 2859 | 1815 | 181545 | 22084 |
| Accu_Line K401 658 line 01 | 240 | 170 | 556 | 93 | 2198 | 39660 | 12978 | 2545 | 217073 | -114 | 2694 | 3434 | 644 | 339 | 60818 | 7608 |
| Accu_Line K401 658 line 02 | 282 | 112 | 709 | 32 | 2034 | 63202 | 13935 | 2796 | 226574 | 172 | 3379 | 4032 | 882 | 626 | 71309 | 8388 |
| Accu_Line K401 658 line 03 | 112 | 257 | 692 | 19 | 1642 | 32212 | 14541 | 2791 | 231927 | 191 | 3108 | 5275 | 2897 | 1991 | 195727 | 23828 |
| Accu_Line K401 658 line 04 | 131 | 107 | 808 | 123 | 1872 | 60795 | 15363 | 3909 | 377872 | 6 | 5233 | 6623 | 2255 | 1622 | 174263 | 21344 |
| Accu_Line K401 658 line 05 | 127 | 311 | 767 | 86 | 1697 | 9408 | 30261 | 2804 | 180220 | 84 | 3629 | 4576 | 2769 | 2515 | 198401 | 24342 |
| Accu_Line K401 658 paper | 47 | 155 | 451 | 310 | 1903 | 1421 | 7713 | 1441 | 5589 | 231 | 1722 | 2496 | 1908 | 1641 | 148674 | 17709 |
| Accu_Line K403 828 line 01 (page 1) | 75 | 189 | 333 | 45 | 1740 | 5580 | 18061 | 804 | 76859 | 104 | 1925 | 3485 | 2459 | 2723 | 185158 | 23141 |
| Accu_Line K403 828 line 02 (page 1) | 157 | 202 | 362 | 277 | 1654 | 1664 | 22114 | 614 | 41847 | -186 | 1933 | 3070 | 2560 | 1979 | 183488 | 21685 |
| Accu_Line K403 828 line 03 (page 1) | 237 | 215 | 493 | 32 | 1715 | 9696 | 16882 | 938 | 117100 | 99 | 1358 | 2506 | 2468 | 1736 | 161056 | 19679 |
| Accu_Line K403 828 line 04 (page 2) | 220 | 158 | 418 | 383 | 1923 | 1676 | 23483 | 1094 | 26891 | 58 | 1807 | 2003 | 1749 | 1767 | 129855 | 15812 |
| Accu_Line K403 828 line 05 (page 2) | 70 | 133 | 528 | 134 | 1779 | 7861 | 20786 | 1144 | 112988 | 389 | 1425 | 3026 | 2891 | 2958 | 204438 | 25140 |
| Accu_Line K403 828 line 06 (page 2) | 253 | 262 | 1166 | 216 | 1718 | 3194 | 27933 | 1063 | 81339 | -77 | 1372 | 2985 | 2932 | 1688 | 186995 | 22424 |
| Accu_Line K403 828 paper (page 1) | 4 | 82 | 234 | 874 | 1728 | 880 | 16915 | 653 | 3969 | 172 | 1692 | 2610 | 1943 | 1547 | 155796 | 18890 |
| Accu_Line K405 1092 line 01 | 92 | 428 | 252 | 31 | 1805 | 15862 | 26563 | 1401 | 59636 | 129 | 1920 | 3053 | 2602 | 2893 | 189599 | 22941 |
| Accu_Line K405 1092 line 02 corrected | 24 | 540 | 165 | 228 | 1510 | 7019 | 31752 | 838 | 38498 | 297 | 1410 | 1825 | 1232 | 1307 | 94382 | 11295 |
| Accu_Line K405 1092 line 03 | 109 | 610 | 314 | 30 | 1826 | 13077 | 28297 | 1440 | 70061 | 298 | 1681 | 2965 | 2652 | 2553 | 197899 | 24426 |
| Accu_Line K405 1092 line 04 (page 3) | 122 | 316 | 311 | 162 | 1550 | 6021 | 24646 | 603 | 24884 | -16 | 1610 | 3118 | 2862 | 3288 | 201058 | 24375 |
| Accu_Line K405 1092 line 05 (page 3) | 70 | 385 | 385 | 223 | 1912 | 13898 | 30397 | 1051 | 60017 | -138 | 1763 | 2549 | 2371 | 1588 | 157063 | 19216 |
| Accu_Line K405 1092 paper | 53 | 116 | 459 | 681 | 1758 | 1945 | 14734 | 882 | 3699 | 167 | 1783 | 2891 | 3003 | 2958 | 200140 | 24735 |
| Accu_Line K407 1298 line 01 corrected | 88 | 238 | 585 | -9 | 1530 | 26189 | 21330 | 786 | 233049 | -118 | 2605 | 2166 | 2321 | 1868 | 173288 | 23227 |
| Accu_Line K407 1298 line 02 corrected | 349 | 268 | 1694 | 76 | 1523 | 106167 | 27397 | 4449 | 849014 | 479 | 5989 | 5195 | 2096 | 2067 | 158244 | 21165 |
| Accu_Line K407 1298 line 03 corrected | 88 | 226 | 490 | -19 | 1792 | 25543 | 21690 | 676 | 147509 | 122 | 2072 | 2012 | 1993 | 1351 | 164098 | 21658 |
| Accu_Line K407 1298 line 04 corrected | 70 | 349 | 483 | 315 | 1653 | 4610 | 33288 | 771 | 51857 | -55 | 1349 | 2099 | 1568 | 1912 | 170835 | 22457 |
| Accu_Line K407 1298 line 05 | 109 | 244 | 617 | 176 | 1790 | 27416 | 19134 | 1509 | 222935 | 147 | 2729 | 3478 | 2782 | 2599 | 200248 | 24940 |
| Accu_Line K407 1298 paper | 50 | 132 | 442 | 783 | 1848 | 1599 | 15739 | 1229 | 4555 | 352 | 1878 | 1577 | 1227 | 949 | 101825 | 12255 |
| Accu_Line K408 1556 line 01 | 216 | 361 | 3244 | 745 | 1811 | 60076 | 34056 | 2957 | 411492 | -59 | 2385 | 1632 | 744 | 13208 | 102492 | 12675 |
| Accu_Line K408 1556 line 02 | 193 | 396 | 2288 | 487 | 1717 | 55636 | 31975 | 2687 | 353427 | 20 | 2428 | 2212 | 1636 | 10409 | 128401 | 16137 |
| Accu_Line K408 1556 line 03 | 96 | 574 | 786 | 104 | 1881 | 7995 | 56608 | 1291 | 114007 | 338 | 2103 | 2368 | 1490 | 1668 | 129222 | 15869 |
| Accu_Line K408 1556 line 04 | 173 | 251 | 576 | 24 | 2013 | 36951 | 23610 | 1618 | 174265 | 77 | 4229 | 1224 | 576 | 558 | 56770 | 6677 |
| Accu_Line K408 1556 line 05 | 69 | 249 | 453 | 21 | 1688 | 20677 | 28398 | 1341 | 121854 | 286 | 3937 | 2771 | 2636 | 2784 | 184744 | 22323 |
| Accu_Line K408 1556 line 06 | 84 | 188 | 700 | 53 | 1708 | 27811 | 25039 | 1336 | 189604 | 364 | 4926 | 2868 | 3169 | 3078 | 207376 | 25790 |
| Accu_Line K408 1556 paper | 6 | 187 | 461 | 568 | 1700 | 1044 | 22195 | 641 | 3194 | 262 | 2205 | 2624 | 2335 | 1995 | 173179 | 21314 |
| Accu_Line K409 1608 line 01 corrected | 132 | 271 | 3494 | 168 | 1518 | 27718 | 27469 | 997 | 261086 | 773 | 38552 | 4479 | 2730 | 2881 | 183490 | 24367 |
| Accu_Line K409 1608 line 03 | 83 | 312 | 1429 | 515 | 1689 | 3184 | 40325 | 1091 | 61749 | 183 | 10027 | 2641 | 2981 | 1955 | 150521 | 17584 |
| Accu_Line K409 1608 line 04 | 40 | 232 | 1053 | 372 | 1584 | 1475 | 33790 | 760 | 39104 | 186 | 6611 | 2398 | 3906 | 2213 | 191293 | 22615 |
| Accu_Line K409 1608 line 05 | 85 | 314 | 4134 | 358 | 1820 | 26282 | 26796 | 2069 | 282372 | 536 | 39586 | 5404 | 5687 | 2984 | 169119 | 21038 |
| Accu_Line K409 1608 paper | 81 | 201 | 390 | 1170 | 1624 | 1284 | 21382 | 977 | 4361 | 602 | 2404 | 2587 | 2242 | 1412 | 131977 | 15920 |
| Accu_Line K412 1902 line 01 | 263 | 340 | 1630 | 929 | 1640 | 25900 | 45163 | 5413 | 477368 | -139 | 1476 | 14712 | 2626 | 2731 | 202463 | 24526 |
| Accu_Line K412 1902 line 02 corrected | 33 | 227 | 997 | 894 | 1654 | 11281 | 30586 | 3104 | 221792 | 41 | 884 | 12218 | 759 | 1063 | 87288 | 11543 |
| Accu_Line K412 1902 line 03 | 123 | 238 | 2191 | 1055 | 1766 | 17672 | 30936 | 3613 | 279980 | -55 | 986 | 11464 | 2082 | 2381 | 161505 | 19488 |
| Accu_Line K412 1902 line 04 | 186 | 382 | 3752 | 414 | 1726 | 30689 | 47497 | 6126 | 502839 | 106 | 1189 | 14085 | 1588 | 1989 | 150785 | 18848 |
| Accu_Line K412 1902 paper corrected | 218 | 238 | 1022 | 1719 | 1803 | 3148 | 14904 | 1218 | 4896 | 47 | 1914 | 7702 | 2084 | 2119 | 161577 | 19293 |
| Accu_Line K415 2098 line 01 | 133 | 198 | 1753 | 105 | 1874 | 31278 | 27327 | 1847 | 194708 | 530 | 9019 | 3224 | 7614 | 863 | 99050 | 12093 |
| Accu_Line K415 2098 line 02 corrected | 164 | 173 | 895 | 66 | 2026 | 27197 | 18749 | 2101 | 144320 | 742 | 8579 | 3931 | 5383 | 2603 | 183193 | 21921 |
| Accu_Line K415 2098 line 03 | 135 | 532 | 2725 | 632 | 1896 | 2643 | 52908 | 853 | 135832 | -4 | 1924 | 2435 | 2313 | 2055 | 159678 | 19144 |
| Accu_Line K415 2098 line 04 | 161 | 259 | 1158 | 365 | 1966 | 6992 | 24961 | 983 | 84589 | 36 | 4530 | 3039 | 5641 | 1879 | 168474 | 20813 |
| Accu_Line K415 2098 paper | 84 | 123 | 415 | 1227 | 2204 | 1800 | 15628 | 756 | 3715 | 227 | 1070 | 618 | 245 | 107 | 37338 | 4688 |
| Accu_Line K415 2182 line 01 | 121 | 256 | 419 | 143 | 1884 | 6771 | 16777 | 1426 | 149245 | 485 | 2831 | 2641 | 1803 | 1570 | 151945 | 17901 |
| Accu_Line K415 2182 line 02 corrected | 123 | 363 | 734 | 55 | 2045 | 11911 | 32007 | 3956 | 288683 | 1107 | 3147 | 1399 | 617 | 438 | 30308 | 4167 |
| Accu_Line K415 2182 line 03 | 135 | 532 | 2725 | 632 | 1896 | 2643 | 52908 | 853 | 135832 | -4 | 1924 | 2435 | 2313 | 2055 | 159678 | 19144 |
| Accu_Line K415 2182 line 04 | 137 | 408 | 1440 | 502 | 1921 | 2198 | 49013 | 1116 | 117652 | 255 | 2049 | 1967 | 618 | 1011 | 92484 | 11380 |
| Accu_Line K415 2182 line 05 | 85 | 208 | 1018 | 497 | 1841 | 945 | 26935 | 869 | 54581 | 241 | 2871 | 2944 | 2504 | 1844 | 170612 | 20626 |
| Accu_Line K415 2182 paper | 101 | 126 | 342 | 1143 | 1848 | 700 | 16650 | 781 | 3017 | -153 | 1120 | 835 | 460 | 1093 | 69745 | 9151 |
| Accu_Line K419 2574 line 01 | 176 | 280 | 505 | 816 | 1585 | 1223 | 30861 | 679 | 23161 | -132 | 963 | 2709 | 1762 | 986 | 183683 | 24303 |
| Accu_Line K419 2574 line 02 | 113 | 326 | 554 | 979 | 1915 | 1492 | 35791 | 597 | 38467 | -282 | 902 | 2546 | 1372 | 681 | 135726 | 17264 |
| Accu_Line K419 2574 line 03 | 75 | 363 | 625 | 607 | 1544 | 1774 | 45040 | 910 | 50838 | 98 | 1245 | 3233 | 1018 | 848 | 151322 | 19889 |
| Accu_Line K419 2574 line 04 | 69 | 390 | 533 | 619 | 1859 | 2266 | 44776 | 1074 | 47962 | -188 | 1158 | 2972 | 492 | 136 | 88734 | 11429 |
| Accu_Line K419 2574 line 05 (page 3) | 170 | 309 | 703 | 915 | 1536 | 1289 | 31299 | 1526 | 30315 | 161 | 1528 | 4836 | 1443 | 1293 | 169588 | 22528 |
| Accu_Line K419 2574 paper | 90 | 201 | 392 | 1089 | 1699 | 1495 | 25796 | 675 | 3006 | 508 | 1336 | 1913 | 1314 | 927 | 165754 | 21813 |
| Accu_Line K420 2868 line 01 | 262 | 725 | 803 | 789 | 1992 | 7592 | 95667 | 824 | 215103 | 258 | 311 | 633 | 249 | -79 | 41934 | 5828 |
| Accu_Line K420 2868 line 02 | 136 | 853 | 782 | 1304 | 1475 | 9588 | 111640 | 1121 | 286572 | 23 | 2007 | 1746 | 1133 | 1541 | 164707 | 21672 |
| Accu_Line K420 2868 line 03 | 152 | 657 | 694 | 958 | 1547 | 6089 | 84722 | 864 | 158908 | 72 | 2296 | 1441 | 1669 | 1742 | 183088 | 24323 |
| Accu_Line K420 2868 line 04 corrected | 216 | 443 | 790 | 562 | 1337 | 7310 | 43620 | 1474 | 111382 | 230 | 2033 | 719 | 376 | 484 | 59985 | 8034 |
| Accu_Line K420 2868 paper | 3 | 84 | 360 | 2291 | 1779 | 1855 | 15765 | 479 | 3827 | -234 | 1051 | 1658 | 1936 | 1097 | 170578 | 22208 |
| Accu_Line K421 3064 line 01 | 178 | 336 | 590 | 144 | 1715 | 2485 | 36749 | 585 | 87763 | 394 | 1203 | 2382 | 1422 | 1239 | 167484 | 22394 |
| Accu_Line K421 3064 line 02 | 107 | 392 | 310 | 94 | 1670 | 5372 | 47805 | 744 | 164205 | -22 | 1078 | 1961 | 1750 | 1672 | 176361 | 23454 |
| Accu_Line K421 3064 line 03 | 72 | 324 | 492 | 23 | 1509 | 1543 | 36105 | 969 | 76615 | -247 | 1695 | 1666 | 2488 | 1458 | 178705 | 23482 |
| Accu_Line K421 3064 line 04 (water damage) | 58 | 300 | 861 | 95 | 1653 | 2208 | 38805 | 1190 | 48194 | -64 | 1992 | 2089 | 2015 | 1440 | 169310 | 22081 |
| Accu_Line K421 3064 line 05 (water damage) | 128 | 616 | 890 | 73 | 1697 | 3685 | 84868 | 453 | 142998 | -174 | 1777 | 1560 | 870 | 1215 | 126410 | 16759 |
| Accu_Line K421 3064 line 06 (water damage) | 33 | 377 | 532 | 19 | 1695 | 1467 | 42987 | 917 | 49619 | -138 | 1397 | 2190 | 2138 | 1381 | 175899 | 23041 |
| Accu_Line K421 3064 paper | 120 | 197 | 371 | 142 | 1718 | 460 | 20009 | 963 | 5355 | 154 | 1306 | 1263 | 757 | 592 | 93806 | 12596 |
| Accu_Line K422 3078 line 01 (signature in poor condition) | 270 | 159 | 399 | 16 | 1692 | 60404 | 20081 | 1049 | 310730 | 1112 | 3736 | 1403 | 1040 | 343 | 117887 | 15244 |
| Accu_Line K422 3078 line 02 (signature in poor condition) | 174 | 263 | 680 | -18 | 1629 | 31406 | 20826 | 775 | 190426 | 456 | 2775 | 2112 | 1418 | 1160 | 155292 | 19824 |
| Accu_Line K422 3078 line 03 | 60 | 169 | 738 | 27 | 1922 | 10370 | 19542 | 471 | 85700 | -137 | 5341 | 5369 | 475 | 1236 | 100473 | 13243 |
| Accu_Line K422 3078 line 04 | 60 | 143 | 1282 | 49 | 2004 | 7769 | 16341 | 828 | 62811 | 159 | 4020 | 4077 | 3471 | 585 | 84979 | 11178 |
| Accu_Line K422 3190 line 01 | 209 | 302 | 2357 | 159 | 1613 | 45157 | 30407 | 1246 | 410731 | 543 | 3125 | 1765 | 1153 | 1321 | 126928 | 16922 |
| Accu_Line K422 3190 line 02 | 601 | 437 | 849 | -1 | 1709 | 36856 | 51892 | 1473 | 321735 | 401 | 2737 | 2261 | 1245 | 1179 | 143290 | 18831 |
| Accu_Line K422 3190 line 03 corrected | 300 | 281 | 383 | 28 | 1675 | 44508 | 41734 | 2111 | 409579 | 681 | 3420 | 2058 | 709 | 766 | 94719 | 12782 |
| Accu_Line K422 3190 line 05 | 260 | 406 | 3131 | 194 | 1843 | 5436 | 33352 | 228 | 100522 | -136 | 18855 | 7662 | 1437 | 925 | 134951 | 17585 |
| Accu_Line K422 3190 line 06 | 34 | 219 | 929 | 225 | 1978 | 2235 | 19265 | 279 | 80923 | 459 | 16985 | 7567 | 1026 | 978 | 155267 | 20325 |
| Accu_Line K423 3234 line 01 | 181 | 179 | 612 | 2 | 1906 | 22179 | 24415 | 1166 | 153491 | 939 | 1687 | 1624 | 552 | 373 | 76501 | 10517 |
| Accu_Line K423 3234 line 02 | 144 | 219 | 618 | 304 | 2110 | 3882 | 26231 | 766 | 44706 | 130 | 1264 | 928 | 579 | 472 | 70039 | 9100 |
| Accu_Line K423 3234 line 03 | 115 | 134 | 830 | 73 | 1939 | 32207 | 14996 | 2041 | 212455 | 1280 | 795 | 2070 | 1189 | 432 | 120689 | 15725 |
| Accu_Line K423 3234 line 04 | 92 | 170 | 517 | 93 | 1758 | 16754 | 23109 | 777 | 128147 | 635 | 1332 | 1700 | 1221 | 1608 | 160928 | 21194 |
| Accu_Line K423 3234 paper | 103 | 116 | 519 | 670 | 1909 | 1245 | 17014 | 896 | 3318 | 234 | 994 | 2083 | 1550 | 1638 | 135020 | 17087 |
| Accu_Line K429 3767 line 01 | 235 | 480 | 821 | 597 | 1644 | 4353 | 65510 | 759 | 228760 | 209 | 4594 | 2421 | 1359 | 1858 | 141426 | 18819 |
| Accu_Line K429 3767 line 02 | 261 | 398 | 1192 | 694 | 1650 | 4256 | 46208 | 450 | 168176 | -50 | 3484 | 1871 | 1242 | 1852 | 137218 | 18507 |
| Accu_Line K429 3767 line 03 | 164 | 479 | 1320 | 480 | 1882 | 5049 | 72727 | 1164 | 278802 | 31 | 5138 | 2629 | 1190 | 914 | 126690 | 16958 |
| Accu_Line K429 3767 line 04 (drawing) | 187 | 645 | 1508 | 357 | 1974 | 5437 | 74111 | 621 | 275512 | -65 | 4358 | 2682 | 1666 | 1379 | 167079 | 21946 |
| Accu_Line K429 3767 line 05 (brown wash) | 79 | 194 | 1314 | 363 | 1862 | 1948 | 27307 | 766 | 29502 | -76 | 1673 | 1930 | 1832 | 1256 | 160096 | 20768 |
| Accu_Line K429 3767 paper | 183 | 174 | 479 | 967 | 2019 | 1613 | 18101 | 812 | 2820 | 139 | 953 | 198 | 171 | 41 | 24369 | 3382 |
| Accu_Line K429 3800 line 01 | 165 | 238 | 2076 | 75 | 1478 | 10960 | 35337 | 1836 | 270346 | -215 | 2282 | 1973 | 1718 | 1295 | 153479 | 20130 |
| Accu_Line K429 3800 line 02 | 130 | 307 | 535 | 112 | 1608 | 4650 | 37998 | 353 | 75665 | 383 | 1362 | 1626 | 1311 | 804 | 154640 | 20647 |
| Accu_Line K429 3800 line 03 | 59 | 345 | 1242 | 155 | 1625 | 9179 | 35210 | 1317 | 174478 | 101 | 2230 | 2333 | 1721 | 1047 | 172131 | 22977 |
| Accu_Line K429 3800 line 04 | 29 | 287 | 1670 | 154 | 1602 | 15364 | 33953 | 2157 | 249257 | 436 | 2600 | 2169 | 974 | 1810 | 147598 | 19995 |
| Accu_Line K429 3800 line 05 | 81 | 207 | 497 | 372 | 1804 | 4271 | 22875 | 417 | 48846 | -189 | 1306 | 2022 | 1958 | 1311 | 175230 | 23085 |
| Accu_Line K429 3800 line 06 | 90 | 140 | 472 | 577 | 1660 | 3023 | 17974 | 293 | 30551 | 28 | 1519 | 1886 | 1968 | 1420 | 176025 | 23344 |
| Accu_Line K429 3800 paper corrected | 226 | 119 | 358 | 1196 | 1629 | 1374 | 15504 | 716 | 3928 | 97 | 1274 | 1868 | 1701 | 2344 | 147543 | 19303 |
| Accu_Line K429 3801 line 01 | 70 | 146 | 2554 | 157 | 1807 | 30853 | 15717 | 1017 | 340306 | 150 | 1681 | 2064 | 2342 | 1329 | 166241 | 22333 |
| Accu_Line K429 3801 line 02 | 61 | 71 | 814 | 159 | 1974 | 5610 | 8619 | 140 | 43064 | -245 | 626 | 1983 | 1532 | 1592 | 170288 | 22362 |
| Accu_Line K429 3801 line 03 | 153 | 159 | 840 | 89 | 1668 | 31415 | 13111 | 548 | 203560 | -18 | 1349 | 1684 | 1875 | 1164 | 150895 | 19897 |
| Accu_Line K429 3801 line 04 | 102 | 3 | 522 | 17 | 1946 | 6469 | 5619 | -2 | 23344 | 25 | 1215 | 1729 | 1568 | 639 | 161481 | 20962 |
| Accu_Line K429 3801 line 05 | 70 | 142 | 629 | 14 | 1938 | 14342 | 20001 | 110 | 130257 | 46 | 1545 | 1512 | 1263 | 521 | 126181 | 16045 |
| Accu_Line K429 3801 paper corrected | 87 | 122 | 328 | 220 | 1992 | 573 | 11701 | 360 | 2804 | -53 | 1555 | 1475 | 1306 | 667 | 148720 | 19709 |
| Accu_Line K430 3842 part 1 line 01 | 157 | 246 | 882 | 38 | 1827 | 19555 | 22461 | 909 | 240456 | 434 | 3539 | 1205 | 571 | 684 | 70851 | 9485 |
| Accu_Line K430 3842 part 1 line 02 | 224 | 290 | 1294 | -13 | 1858 | 42972 | 31957 | 1182 | 422861 | 510 | 6271 | 936 | 203 | 524 | 37885 | 5326 |
| Accu_Line K430 3842 part 1 line 03 | 222 | 276 | 909 | 24 | 1542 | 45653 | 29769 | 1737 | 446842 | 630 | 8345 | 2268 | 1378 | 1365 | 168709 | 22875 |
| Accu_Line K430 3842 part 1 line 04 corrected | 124 | 301 | 943 | -8 | 1593 | 29506 | 34571 | 333 | 136440 | 154 | 2380 | 1650 | 1509 | 1155 | 150088 | 19728 |
| Accu_Line K430 3842 part 1 line 05 corrected | 176 | 413 | 739 | 92 | 1768 | 16465 | 30454 | 583 | 92621 | 31 | 1637 | 987 | 687 | 234 | 91187 | 11840 |
| Accu_Line K430 3842 part 1 line 06 | 135 | 271 | 618 | 76 | 1699 | 17543 | 28935 | 522 | 99186 | 198 | 1869 | 1208 | 625 | 610 | 74854 | 9554 |
| Accu_Line K430 3842 part 1 paper | 105 | 139 | 517 | 275 | 1877 | 1407 | 18939 | 510 | 3511 | 207 | 1247 | 1719 | 1216 | 893 | 110076 | 14063 |

# Results graphs

Compton ratios: [net peak area*1000/Compton net peak area] except for iron where [net peak area*100/Compton net peak area] in order to reduce the size of the iron bars in the chart to improve legibility. All absent peaks were set to 0. In some cases the difference between a peak near background level or an absent peak is marginal and not always obvious. Absent peaks can simply mean present below limit of detection.
