# Supplementary material for: A closer look at the Azzolino collection
Source: PLoS One. 2023 Apr 12;18(4):e0283539. doi: 10.1371/journal.pone.0283539 (PMC10096476; doi:10.1371/journal.pone.0283539)

**Datum** 2020-07-21

**Dnr** 3.5.1-04051-2017

**Fyndnr.**

**Löpnr:**

**Handläggare**: Tom Sandström

**Författare**: Marei Hacke

**Riksantikvarieämbetet**

Artillerigatan 33
Box 1114
621 22 Visby

**Tel** 08-5191 8000

**E-post** riksant@raa.se

**Hemsida** www.raa.se

**Org.nr** 202100-1090

**Plusgiro** 59994-4

**Bankgiro** 5052-3620

**µ- XRF instrument report quantitative analysis**

**Date of analysis** 2019-04-03

**Analyst** Tom Sandström and Kaj Thuresson

**Table of contents**

Samples 2

Purpose 3

Method 3

Sample preparation 3

Instrument parameters 4

Method for quantifying metal content ink on paper 4

Method for calculating ink ratios 5

Results 7

Calibration slopes for metal impregnated papers 7

Iron quantification 7

Copper quantification 9

Manganese quantification 11

Zinc quantification 13

Net counts quantification results for Azzolino collection analyses 15

Net counts and concentration metal/paper (mg/g) 15

Compton ratios quantification results for Azzolino collection analyses 19

Compton ratios and concentration metal/paper (mg/g) 19

Results of ink ratios based on net peak quantification 23

Results of ink ratios based on Compton normalisation quantification 24

Discussion 25

Net peak area versus Compton normalisation for quantification of concentration metal/paper (mg/g) 26

Ink ratios calculated from quantification slopes giving weight/weight content of manganese, copper and zinc in relation to iron 27

# Samples

**RAÄ Dnr 3.5.1-04051-2017**

**Riksarkivet Dnr 2017/7960**

**Description of samples** Metal impregnated papers were prepared in the laboratory of Veronique Rouchon, Centre de Recherche sur la Conservation (CNRS) and posted to the heritage laboratory in September 2018.

**Age** new

**Material** filter paper impregnated with solutions of iron, copper, manganese and zinc

**Concentrations** were determined by CNRS and are listed in the tables on the images below.

**Point of analysis** five points randomly selected on each paper


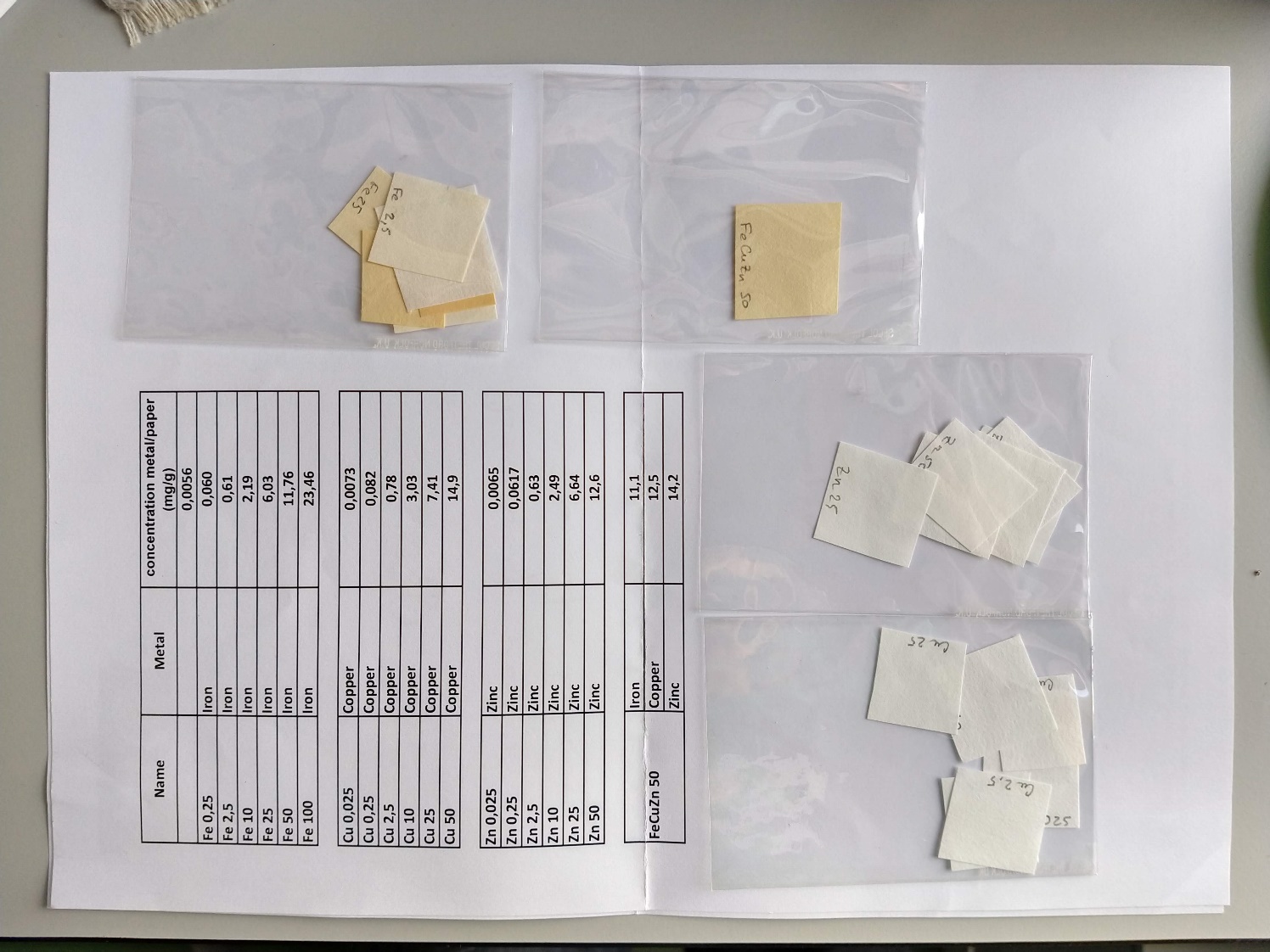


Photograph of the samples with iron, copper and zinc as received from CNRS (photo: Thea Winther)


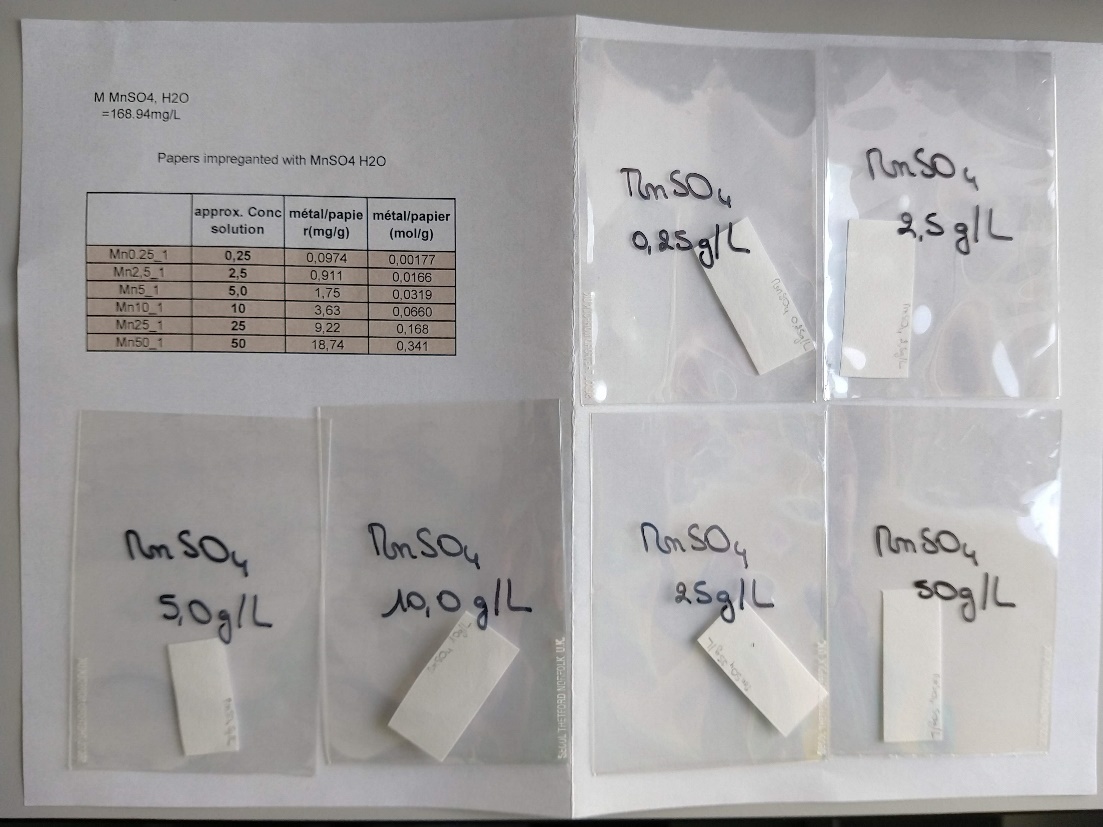


Photograph of the samples with manganese as received from CNRS (photo: Thea Winther)

# Purpose

XRF analyses of the papers impregnated with metals were carried out in order to establish quantification reference slopes that would allow quantification of the metallic components in the iron gall inks in the Azzolino collection.

# Method

## Sample preparation

The single sheets were supported on a plastic box made of PET, which gave a near blank background of Bremsstrahlung. Note that molybdenum and argon are associated with the XRF instrument and are present in every spectrum.

XRF spectrum of background plastic box (PET)

## Instrument parameters

**x**  µ-XRF Artax 800, Mo X-ray tube with polycapillary lens, Bruker; Berlin, Germany

  single point analysis (spot size <100µm)

**x**  line scan (lateral resolution <100µm)

**x**  elemental 2D mapping

  quantification, MQuant Calib, Bruker; Berlin, Germany

  quantification with standards

**Voltage** 50 KV

**Current** 600 µA

**Filter x**  no filter   Al 315 µm   Mo 12.50 µm   other ______

**Lens** 0.060

**Atmosphere x**  air   He for light element detection

**Life time** 10 s

**Number of measurement points** 5

## Method for quantifying metal content ink on paper

- The XRF spectra from 5 measurement points on each of the metal impregnated papers were accumulated and then automatically corrected for escape peaks and background using cycle setting 1. The live time was 10 s for all spectra, i.e. 50 s total in the accumulation spectra.
- Net peak areas were calculated from the accumulated spectra using the same ROI (regions of interest) as those used for the investigated Azzolino collection objects. The start and end keV for each ROI was set as follows:
  - Start/keV End/keV Peak name
  - 2,179 2,444 S_K
  - 2,449 2,737 Cl_K
  - 2,774 3,115 Ar_K
  - 3,099 3,492 K_K
  - 3,484 3,873 Ca_K
  - 5,698 6,07 Mn_K
  - 6,082 6,698 Fe_K
  - 7,789 8,327 Cu_K
  - 8,425 8,836 Zn_K
  - 15,774 17,128 Compton
  - 17,155 17,797 Mo_K
  - 10,183 10,871 Pb_L
  - 7,307 7,672 Ni_K
  - 9,799 10,175 Hg_L
  - 1,907 2,099 P_K
  - 1,642 1,847 Si_K
- In calculating net peak areas from ROIs values are obtained even for non-existent peaks as some counts are present between the noise of the spectral line and the generated background line. Only values obtained for the elements of interest (Fe, Mn, Cu, Zn) are listed in the results tables.
- The overall count rate may be affected by variations in the paper, the composition of the inks and/or impregnation materials (matrix effects) and by variations in detector deadtime. This can be normalised by dividing the net counts of the peaks of interest by the net counts of the Compton peak. The Compton peak is a part of the Bremsstrahlung background.
- Two sets of linear regression calibration lines for quantification were calculated using:
  - Net counts
  - Compton ratios
- The results from the Azzolino collection XRF analyses were plotted on both sets of calibration slopes.

Net counts quantification slopes of metal impregnated papers

| Mn quantification linear regression | Fe quantification linear regression | Cu quantification linear regression | Zn quantification linear regression |
| --- | --- | --- | --- |
| y = 0.0000658370x - 0.2905381676 | y = 0.0000575263x - 0.3766793858 | y = 0.0000584179x - 0.2087439957 | y = 0.0000702614x - 0.3688741330 |
| R² = 0.9992365074 | R² = 0.9960310307 | R² = 0.9997659494 | R² = 0.9954618958 |

Compton ratios quantification slopes of metal impregnated papers

| Mn quantification linear regression | Fe quantification linear regression | Cu quantification linear regression | Zn quantification linear regression |
| --- | --- | --- | --- |
| y = 0.0053970068x - 0.4830118483 | y = 0.0048044480x - 0.4587093236 | y = 0.0047860850x - 0.2453681408 | y = 0.0058732263x - 0.3008625924 |
| R² = 0.9876043400 | R² = 0.9988929449 | R² = 0.9987489891 | R² = 0.9973044119 |

## Method for calculating ink ratios

The method for calculating the weight/weight ratio of Mn, Cu and Zn in relation to Fe is based on calculations of slope ratios (as suggested by Veronique Rouchon during a project meeting on 2019-09-09 in Paris).

Ratios of weight metal to weight iron were calculated by multiplying the slope of metal/iron net peak areas by the slope of the quantification metal divided by the slope of quantification iron.

For example: slope Mn/Fe * Quants_slopeMn/Quant_slopeFe

Question:

- What happens to the constants (b values)? Can you really disregard them totally?

Example for object (letter K394 0014) where five areas were measured by XRF (accumulation of five points per area)

Mn/Fe slope for K394 0014
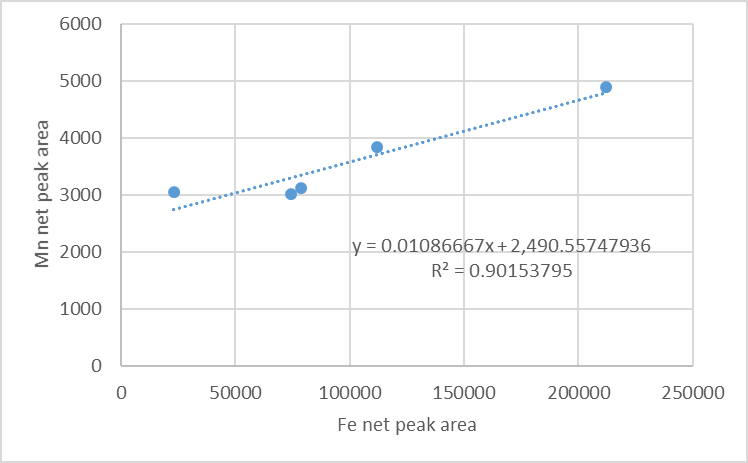


Cu/Fe slope for K394 0014
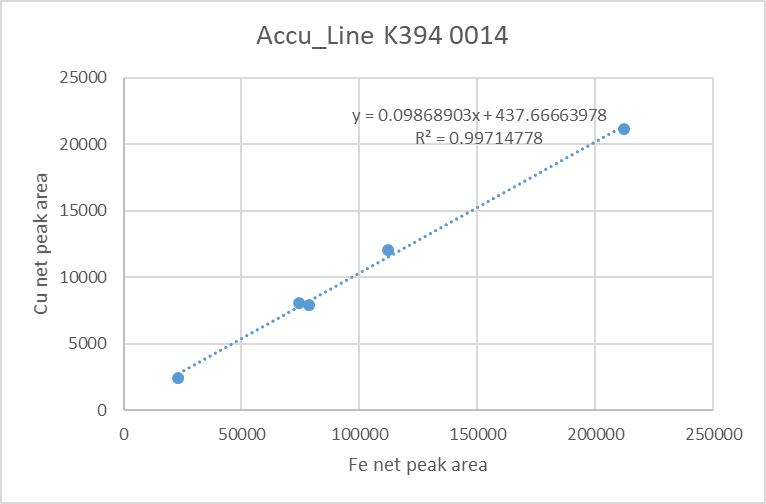


Zn/Fe slope for K394 0014
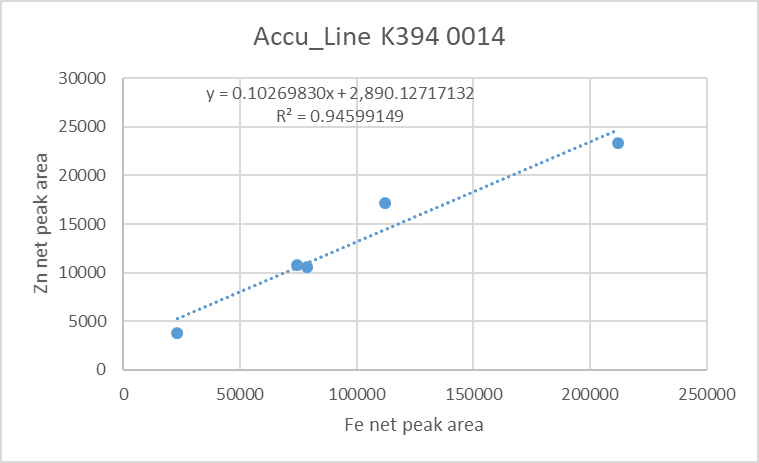


K394 0014 slope Mn/Fe * Quants_slopeMn/Quant_slopeFe

0.01086667*0.000065837/0.0000575263 = 0.012436554

K394 0014 slope Cu/Fe * Quants_slopeCu/Quant_slopeFe

0.09868903*0.0000584179/0.0000575263 = 0.100218611

K394 0014 slope Zn/Fe * Quants_slopeZn/Quant_slopeFe

0.10269830*0.0000702614/0.0000575263 = 0.125433521

# Results

## Calibration slopes for metal impregnated papers

### Iron quantification


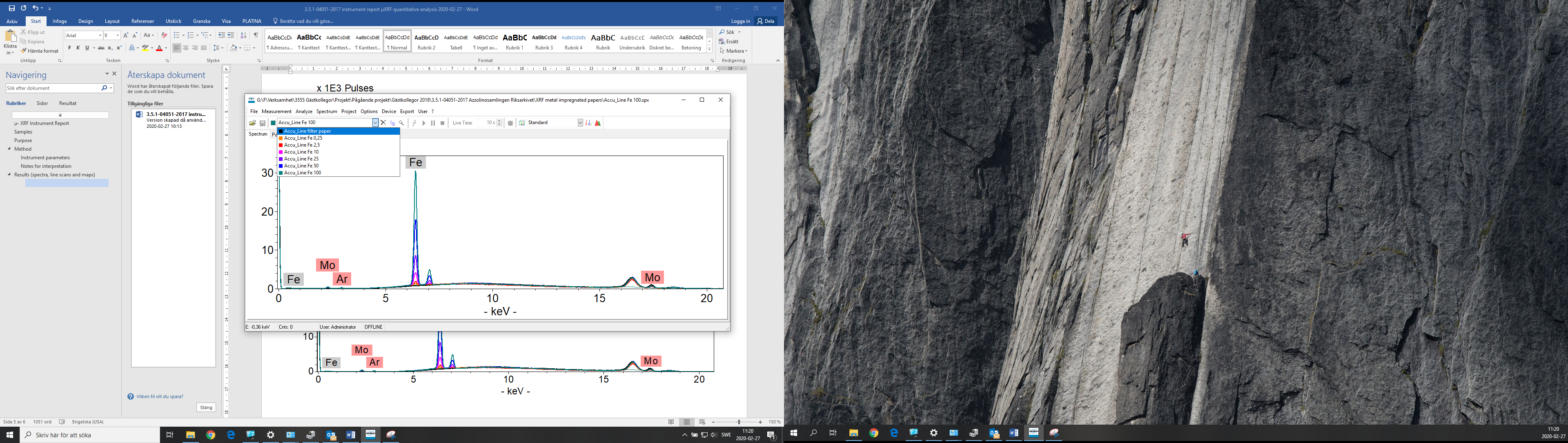


| **Gross** | **Net** | **Backgr.** | **Spectrum** | **Concentration metal/paper (mg/g)** |
| --- | --- | --- | --- | --- |
| 38447 | 1708 | 36739 | Accu_Line filter paper | 0 |
| 49378 | 6917 | 42461 | Accu_Line Fe 0,25 | 0.06 |
| 54718 | 16063 | 38655 | Accu_Line Fe 2,5 | 0.61 |
| 88641 | 45739 | 42902 | Accu_Line Fe 10 | 2.19 |
| 150592 | 107323 | 43269 | Accu_Line Fe 25 | 6.03 |
| 280137 | 230812 | 49325 | Accu_Line Fe 50 | 11.76 |
| 453366 | 404054 | 49312 | Accu_Line Fe 100 | 23.46 |


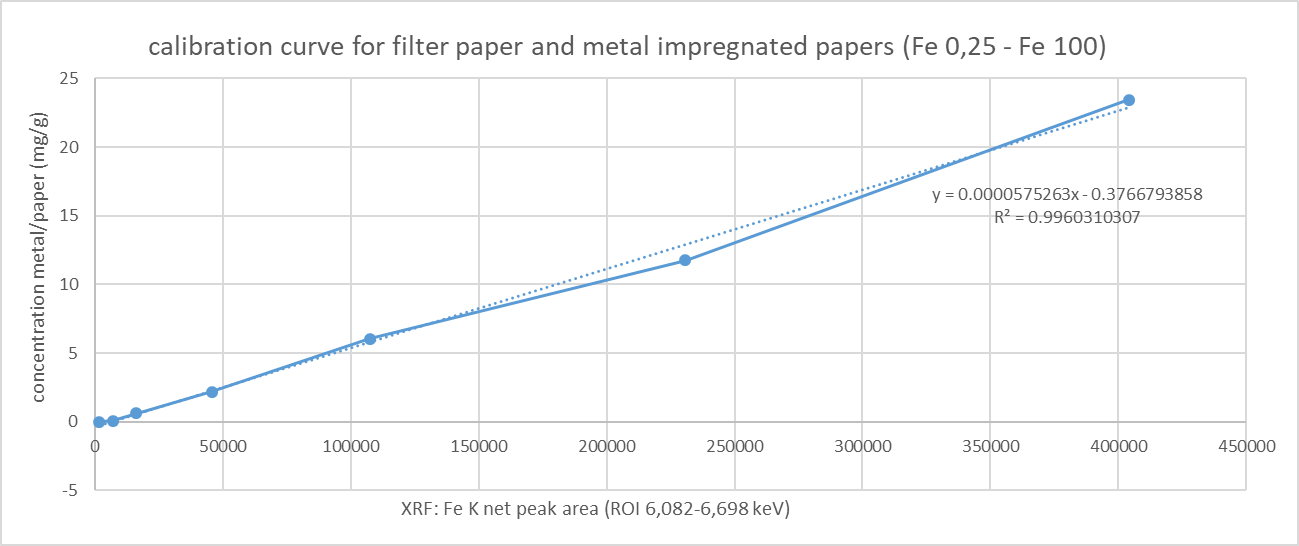


| **Compton net counts** | **Fe_K**  **net counts** | **Compton ratio x1000** | **Spectrum** | **Concentration metal/paper (mg/g)** |
| --- | --- | --- | --- | --- |
| 89491 | 1708 | 19.08572 | Accu_Line filter paper | 0 |
| 78883 | 6917 | 87.68683 | Accu_Line Fe 0,25 | 0.06 |
| 69461 | 16063 | 231.2521 | Accu_Line Fe 2,5 | 0.61 |
| 78045 | 45739 | 586.0593 | Accu_Line Fe 10 | 2.19 |
| 78616 | 107323 | 1365.155 | Accu_Line Fe 25 | 6.03 |
| 87306 | 230812 | 2643.713 | Accu_Line Fe 50 | 11.76 |
| 82184 | 404054 | 4916.456 | Accu_Line Fe 100 | 23.46 |


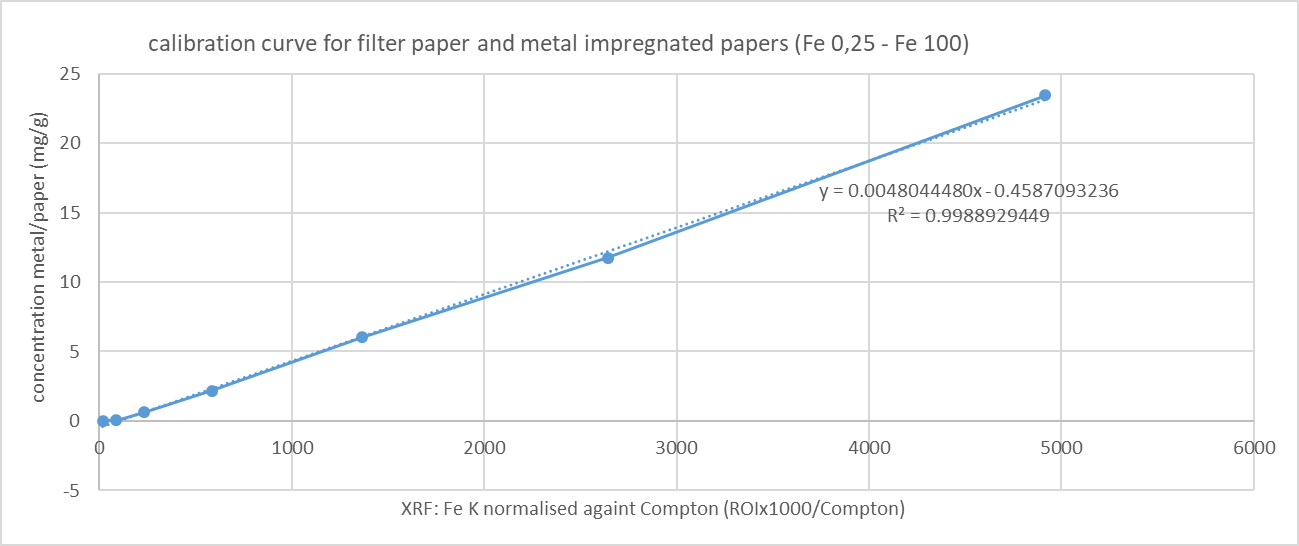


### Copper quantification


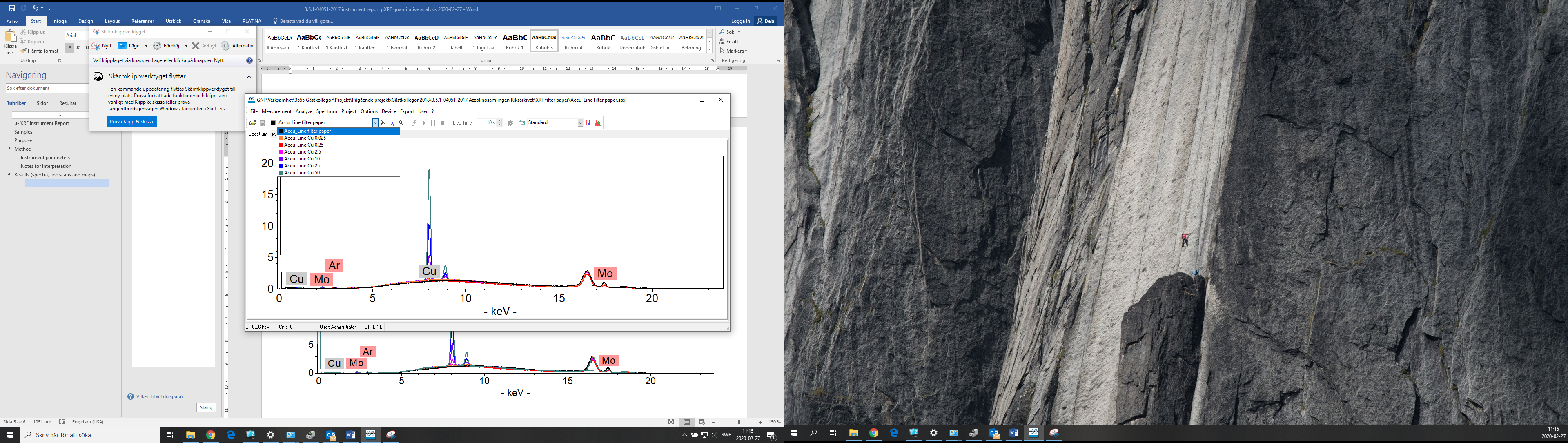


| **Gross** | **Net** | **Backgr.** | **Spectrum** | **Concentration metal/paper (mg/g)** |
| --- | --- | --- | --- | --- |
| 51730 | 983 | 50747 | Accu_Line filter paper | 0 |
| 61559 | 3062 | 58497 | Accu_Line Cu 0,025 | 0.0073 |
| 56653 | 7223 | 49430 | Accu_Line Cu 0,25 | 0.082 |
| 72672 | 17408 | 55264 | Accu_Line Cu 2,5 | 0.78 |
| 115842 | 56119 | 59723 | Accu_Line Cu 10 | 3.03 |
| 187494 | 130509 | 56985 | Accu_Line Cu 25 | 7.41 |
| 318599 | 258361 | 60238 | Accu_Line Cu 50 | 14.9 |


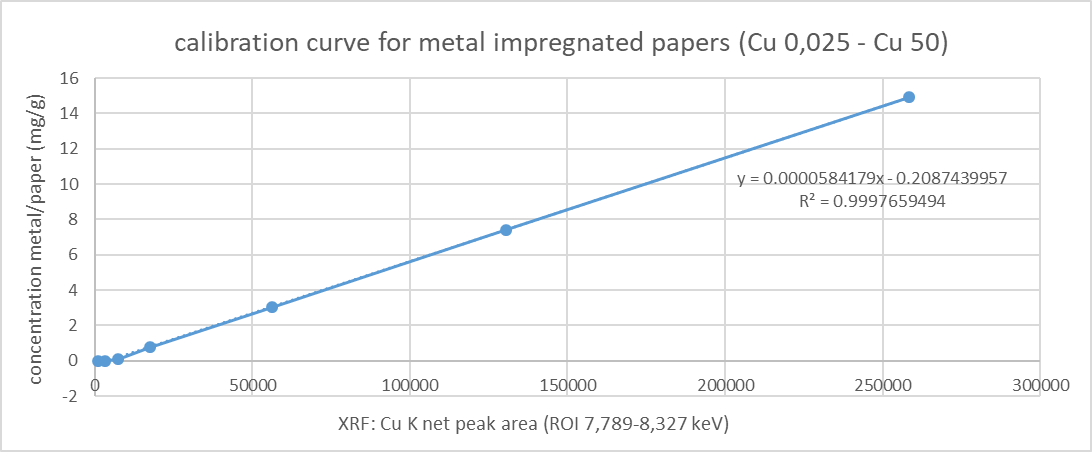


| **Compton net counts** | **Cu_K**  **net counts** | **Compton ratio x1000** | **Spectrum** | **Concentration metal/paper (mg/g)** |
| --- | --- | --- | --- | --- |
| 89491 | 983 | 10.98434 | Accu_Line filter paper | 0 |
| 85297 | 3062 | 35.8981 | Accu_Line Cu 0,025 | 0.0073 |
| 69911 | 7223 | 103.3171 | Accu_Line Cu 0,25 | 0.082 |
| 78863 | 17408 | 220.7372 | Accu_Line Cu 2,5 | 0.78 |
| 84683 | 56119 | 662.695 | Accu_Line Cu 10 | 3.03 |
| 78018 | 130509 | 1672.806 | Accu_Line Cu 25 | 7.41 |
| 82581 | 258361 | 3128.577 | Accu_Line Cu 50 | 14.9 |


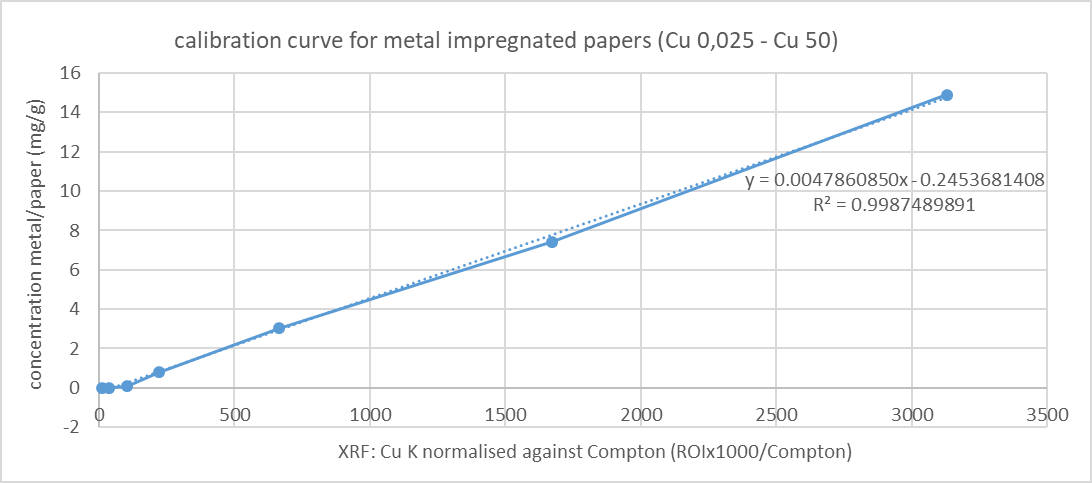


### Manganese quantification


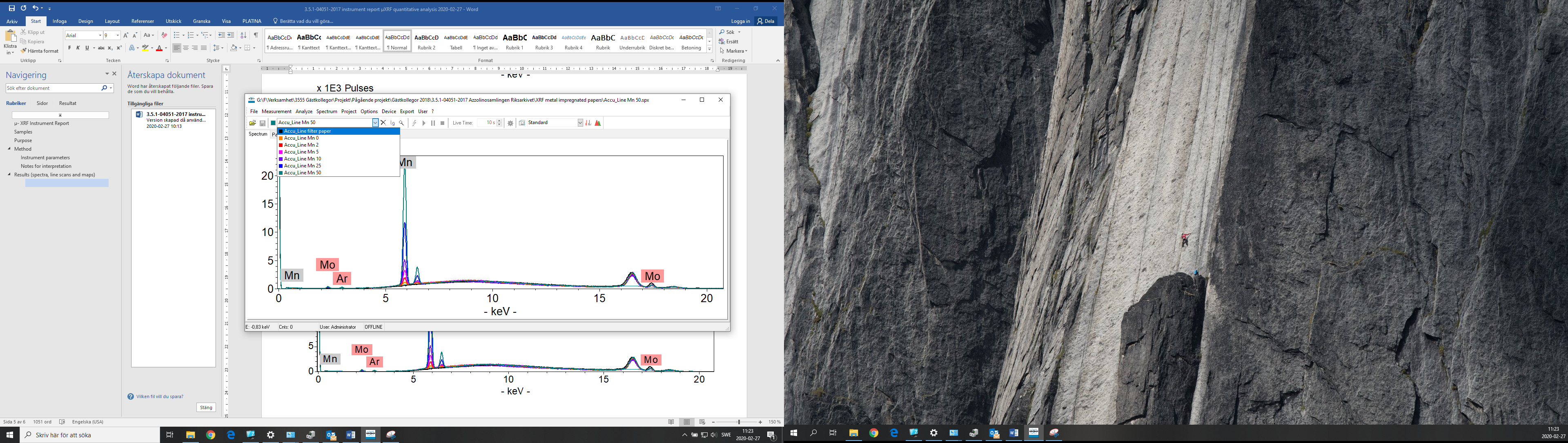


| **Gross** | **Net** | **Backgr.** | **Spectrum** | **Concentration metal/paper (mg/g)** |
| --- | --- | --- | --- | --- |
| 16859 | 356 | 16503 | Accu_Line filter paper | 0 |
| 26371 | 6396 | 19975 | Accu_Line Mn 0 | 0.0974 |
| 37138 | 16857 | 20281 | Accu_Line Mn 2 | 0.911 |
| 55296 | 34005 | 21291 | Accu_Line Mn 5 | 1.75 |
| 78584 | 59890 | 18694 | Accu_Line Mn 10 | 3.63 |
| 168775 | 148497 | 20278 | Accu_Line Mn 25 | 9.22 |
| 309907 | 286609 | 23298 | Accu_Line Mn 50 | 18.74 |


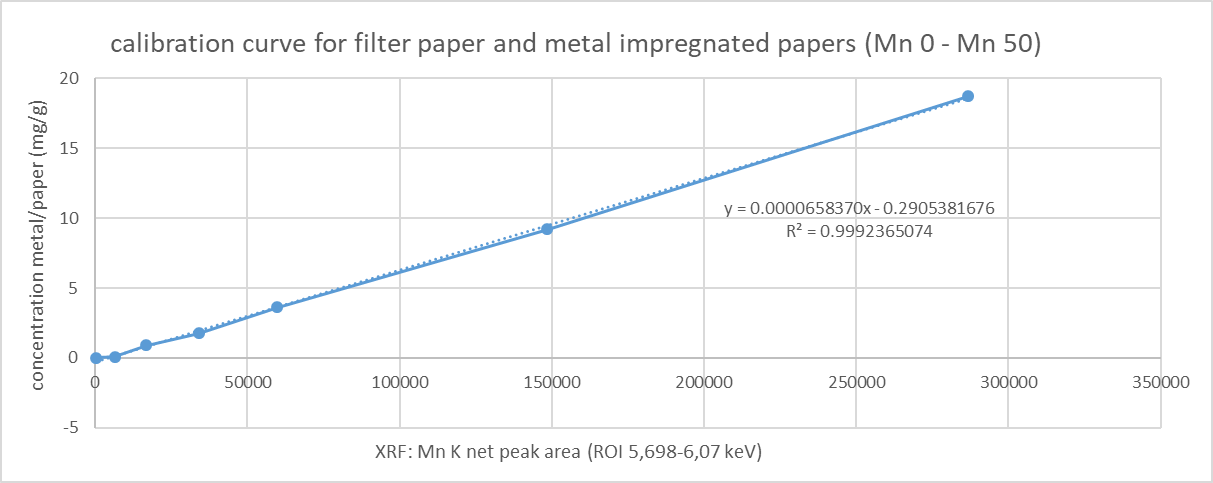


| **Compton net counts** | **Mn_K**  **net counts** | **Compton ratio x1000** | **Spectrum** | **Concentration metal/paper (mg/g)** |
| --- | --- | --- | --- | --- |
| 89491 | 356 | 3.978054 | Accu_Line filter paper | 0 |
| 80425 | 6396 | 79.52751 | Accu_Line Mn 0 | 0.0974 |
| 81403 | 16857 | 207.0808 | Accu_Line Mn 2 | 0.911 |
| 86297 | 34005 | 394.0461 | Accu_Line Mn 5 | 1.75 |
| 69270 | 59890 | 864.5878 | Accu_Line Mn 10 | 3.63 |
| 72151 | 148497 | 2058.142 | Accu_Line Mn 25 | 9.22 |
| 84709 | 286609 | 3383.454 | Accu_Line Mn 50 | 18.74 |


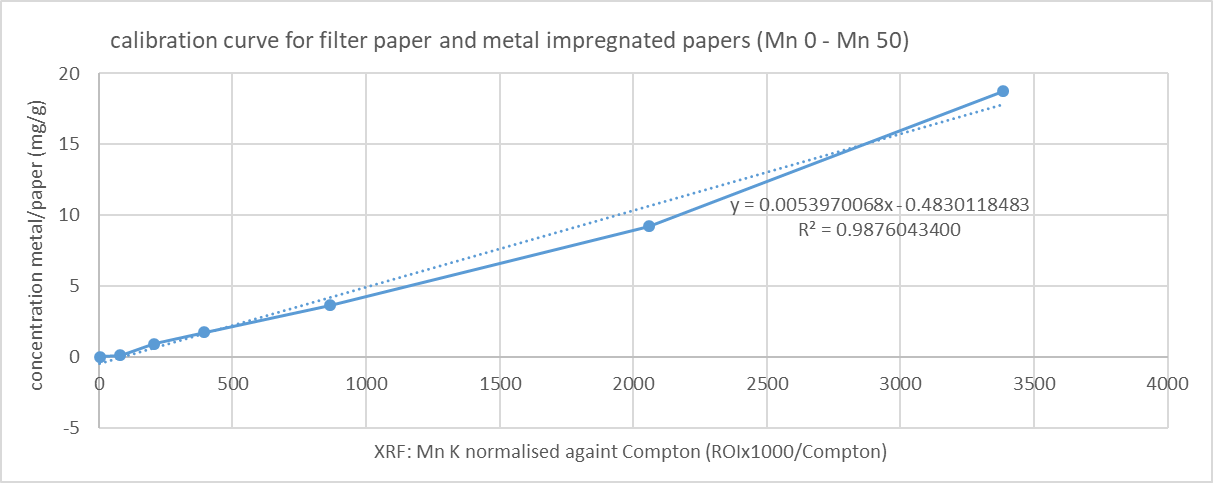


### Zinc quantification


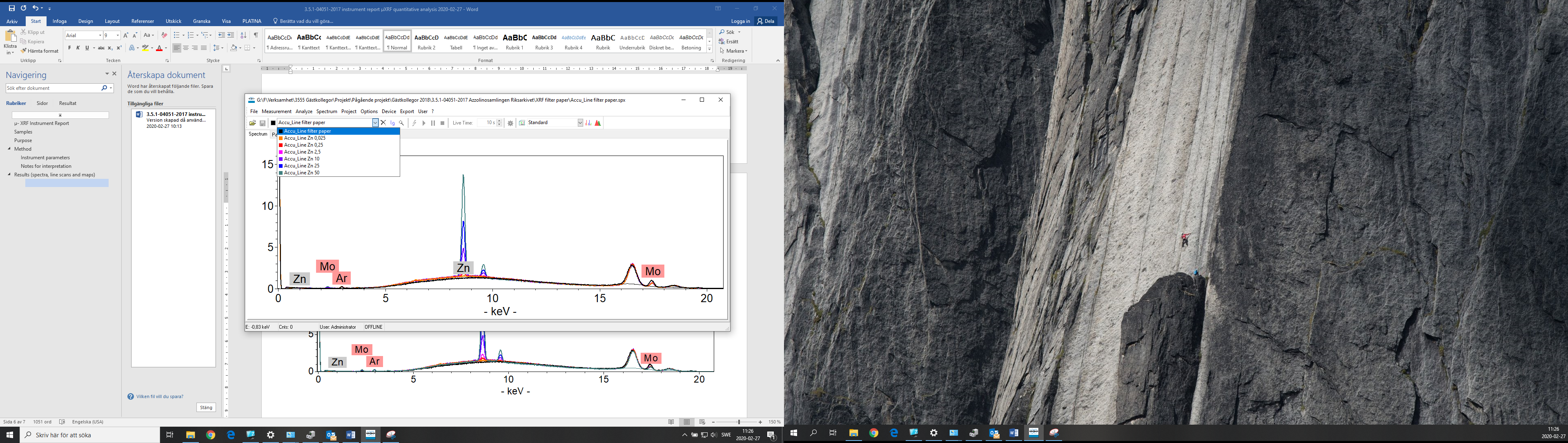


| **Gross** | **Net** | **Backgr.** | **Spectrum** | **Concentration metal/paper (mg/g)** |
| --- | --- | --- | --- | --- |
| 42484 | 639 | 41845 | Accu_Line filter paper | 0 |
| 50106 | 2634 | 47472 | Accu_Line Zn 0,025 | 0.0065 |
| 55377 | 6364 | 49013 | Accu_Line Zn 0,25 | 0.0617 |
| 62330 | 14693 | 47637 | Accu_Line Zn 2,5 | 0.63 |
| 100857 | 50285 | 50572 | Accu_Line Zn 10 | 2.49 |
| 148815 | 98986 | 49829 | Accu_Line Zn 25 | 6.64 |
| 230552 | 182360 | 48192 | Accu_Line Zn 50 | 12.6 |


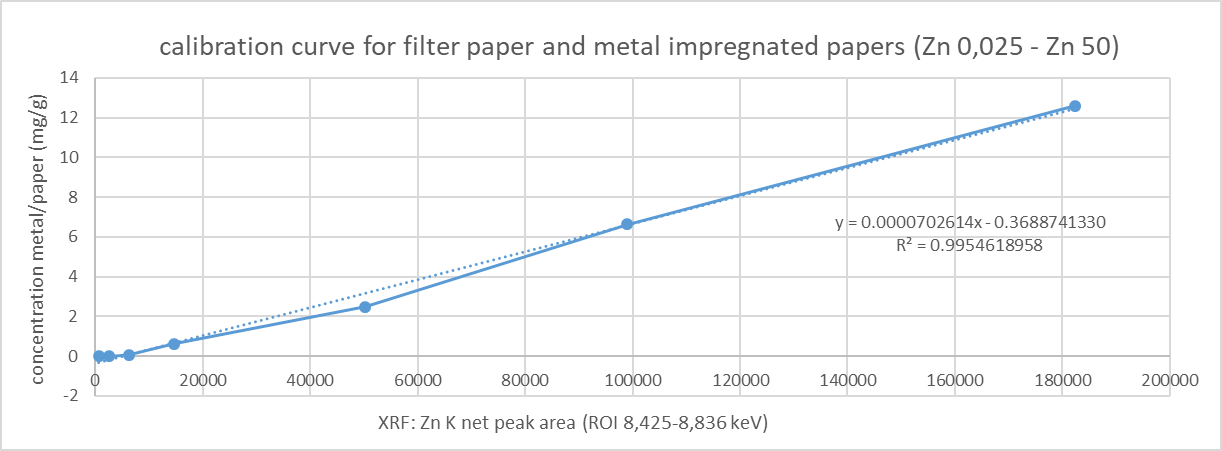


| **Compton net counts** | **Zn_K**  **net counts** | **Compton ratio x1000** | **Spectrum** | **Concentration metal/paper (mg/g)** |
| --- | --- | --- | --- | --- |
| 89491 | 639 | 7.140383 | Accu_Line filter paper | 0 |
| 86759 | 2634 | 30.35996 | Accu_Line Zn 0,025 | 0.0065 |
| 89378 | 6364 | 71.2032 | Accu_Line Zn 0,25 | 0.0617 |
| 86750 | 14693 | 169.3718 | Accu_Line Zn 2,5 | 0.63 |
| 89993 | 50285 | 558.7657 | Accu_Line Zn 10 | 2.49 |
| 86191 | 98986 | 1148.449 | Accu_Line Zn 25 | 6.64 |
| 83193 | 182360 | 2192.011 | Accu_Line Zn 50 | 12.6 |


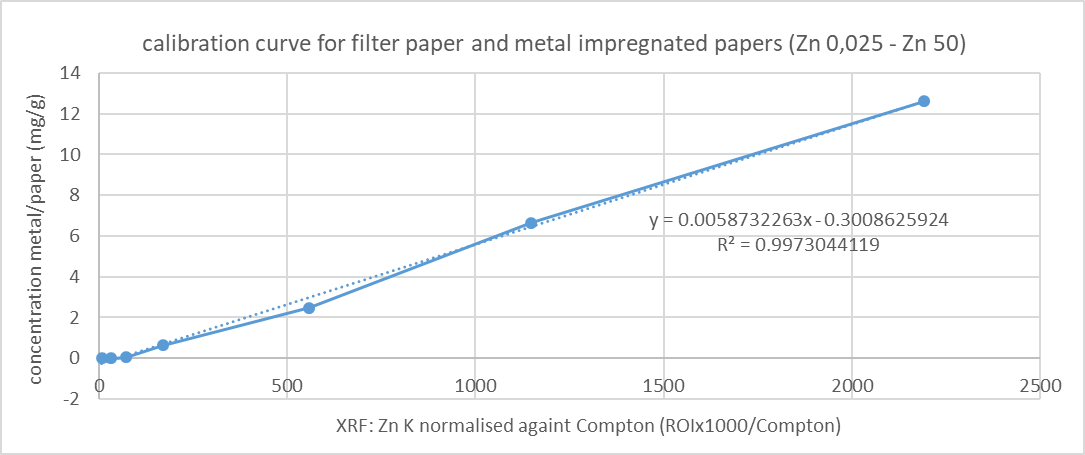


## Net counts quantification results for Azzolino collection analyses

| Mn quantification linear regression | Fe quantification linear regression | Cu quantification linear regression | Zn quantification linear regression |
| --- | --- | --- | --- |
| y = 0.0000658370x - 0.2905381676 | y = 0.0000575263x - 0.3766793858 | y = 0.0000584179x - 0.2087439957 | y = 0.0000702614x - 0.3688741330 |
| R² = 0.9992365074 | R² = 0.9960310307 | R² = 0.9997659494 | R² = 0.9954618958 |

### Net counts and concentration metal/paper (mg/g)

| Spectrum | Mn_K Net counts | Mn concentration metal/paper (mg/g) | Fe_K Net counts | Fe concentration metal/paper (mg/g) | Cu_K Net counts | Cu concentration metal/paper (mg/g) | Zn_K Net counts | Zn concentration metal/paper (mg/g) |
| --- | --- | --- | --- | --- | --- | --- | --- | --- |
| Del Monte Vol II A1 No 17 _ 01 | 1617 | -0.18 | 143444 | 7.88 | 2185 | -0.08 | 3627 | -0.11 |
| Del Monte Vol II A1 No 17 _ 02 | 1609 | -0.18 | 163023 | 9.00 | 1774 | -0.11 | 3351 | -0.13 |
| Del Monte Vol II A1 No 17 _ 03 | 1409 | -0.20 | 178056 | 9.87 | 1899 | -0.10 | 3223 | -0.14 |
| Del Monte Vol II A1 No 17 _ 04 | 1842 | -0.17 | 264498 | 14.84 | 1539 | -0.12 | 3970 | -0.09 |
| Del Monte Vol II A1 No 17 paper | 1022 | -0.22 | 4060 | -0.14 | 1990 | -0.09 | 2817 | -0.17 |
| K394 0014 _ 01 | 3005 | -0.09 | 74351 | 3.90 | 8022 | 0.26 | 10803 | 0.39 |
| K394 0014 _ 02 | 4886 | 0.03 | 212247 | 11.83 | 21141 | 1.03 | 23393 | 1.27 |
| K394 0014 _ 03 | 3051 | -0.09 | 23100 | 0.95 | 2435 | -0.07 | 3821 | -0.10 |
| K394 0014 _ 04 | 3119 | -0.09 | 78561 | 4.14 | 7935 | 0.25 | 10598 | 0.38 |
| K394 0014 _ 05 | 3828 | -0.04 | 112006 | 6.07 | 12026 | 0.49 | 17212 | 0.84 |
| K394 0014 paper | 2667 | -0.11 | 6532 | 0.00 | 411 | -0.18 | 523 | -0.33 |
| K396 266 _ 01 | 1401 | -0.20 | 215054 | 11.99 | 2191 | -0.08 | 1405 | -0.27 |
| K396 266 _ 02 | 1021 | -0.22 | 141336 | 7.75 | 2290 | -0.07 | 2800 | -0.17 |
| K396 266 _ 03 | 1288 | -0.21 | 189122 | 10.50 | 2852 | -0.04 | 2074 | -0.22 |
| K396 266 _ 04 | 1007 | -0.22 | 208373 | 11.61 | 2809 | -0.04 | 2108 | -0.22 |
| K396 266 _ 05 corrected | 2056 | -0.16 | 305356 | 17.19 | 5599 | 0.12 | 1703 | -0.25 |
| K396 266 paper | 736 | -0.24 | 4699 | -0.11 | 1676 | -0.11 | 2954 | -0.16 |
| K397 364 _ 01 | 3953 | -0.03 | 177338 | 9.82 | 1733 | -0.11 | 5134 | -0.01 |
| K397 364 _ 02 | 3499 | -0.06 | 127651 | 6.97 | 1855 | -0.10 | 3897 | -0.10 |
| K397 364 _ 03 | 4172 | -0.02 | 187908 | 10.43 | 1789 | -0.10 | 5107 | -0.01 |
| K397 364 _ 04 | 2908 | -0.10 | 109701 | 5.93 | 1583 | -0.12 | 3579 | -0.12 |
| K397 364 _ 05 | 2400 | -0.13 | 108170 | 5.85 | 988 | -0.15 | 2615 | -0.19 |
| K397 364 paper | 2327 | -0.14 | 6312 | -0.01 | 2347 | -0.07 | 2767 | -0.17 |
| K399 470 _ 01 | 1330 | -0.20 | 95848 | 5.14 | 1651 | -0.11 | 2163 | -0.22 |
| K399 470 _ 02 | 811 | -0.24 | 97921 | 5.26 | 1652 | -0.11 | 2124 | -0.22 |
| K399 470 _ 03 | 567 | -0.25 | 63477 | 3.27 | 1555 | -0.12 | 2792 | -0.17 |
| K399 470 _ 04 | 1431 | -0.20 | 118485 | 6.44 | 2188 | -0.08 | 2782 | -0.17 |
| K399 470 _ 05 | 1070 | -0.22 | 75423 | 3.96 | 2266 | -0.08 | 2715 | -0.18 |
| K399 470 paper | 571 | -0.25 | 5265 | -0.07 | 2386 | -0.07 | 2642 | -0.18 |
| K401 658 _ 01 | 2545 | -0.12 | 217073 | 12.11 | 2694 | -0.05 | 3434 | -0.13 |
| K401 658 _ 02 | 2796 | -0.11 | 226574 | 12.66 | 3379 | -0.01 | 4032 | -0.09 |
| K401 658 _ 03 | 2791 | -0.11 | 231927 | 12.97 | 3108 | -0.03 | 5275 | 0.00 |
| K401 658 _ 04 | 3909 | -0.03 | 377872 | 21.36 | 5233 | 0.10 | 6623 | 0.10 |
| K401 658 _ 05 | 2804 | -0.11 | 180220 | 9.99 | 3629 | 0.00 | 4576 | -0.05 |
| K401 658 paper | 1441 | -0.20 | 5589 | -0.06 | 1722 | -0.11 | 2496 | -0.19 |
| K403 828 _ 01 | 804 | -0.24 | 76859 | 4.04 | 1925 | -0.10 | 3485 | -0.12 |
| K403 828 _ 02 | 614 | -0.25 | 41847 | 2.03 | 1933 | -0.10 | 3070 | -0.15 |
| K403 828 _ 03 | 938 | -0.23 | 117100 | 6.36 | 1358 | -0.13 | 2506 | -0.19 |
| K403 828 _ 04 | 1094 | -0.22 | 26891 | 1.17 | 1807 | -0.10 | 2003 | -0.23 |
| K403 828 _ 05 | 1144 | -0.22 | 112988 | 6.12 | 1425 | -0.13 | 3026 | -0.16 |
| K403 828 _ 06 | 1063 | -0.22 | 81339 | 4.30 | 1372 | -0.13 | 2985 | -0.16 |
| K403 828 paper | 653 | -0.25 | 3969 | -0.15 | 1692 | -0.11 | 2610 | -0.19 |
| K405 1092 _ 01 | 1401 | -0.20 | 59636 | 3.05 | 1920 | -0.10 | 3053 | -0.15 |
| K405 1092 _ 02 corrected | 838 | -0.24 | 38498 | 1.84 | 1410 | -0.13 | 1825 | -0.24 |
| K405 1092 _ 03 | 1440 | -0.20 | 70061 | 3.65 | 1681 | -0.11 | 2965 | -0.16 |
| K405 1092 _ 04 | 603 | -0.25 | 24884 | 1.05 | 1610 | -0.11 | 3118 | -0.15 |
| K405 1092 _ 05 | 1051 | -0.22 | 60017 | 3.08 | 1763 | -0.11 | 2549 | -0.19 |
| K405 1092 paper | 882 | -0.23 | 3699 | -0.16 | 1783 | -0.10 | 2891 | -0.17 |
| K407 1298 _ 01 corrected | 786 | -0.24 | 233049 | 13.03 | 2605 | -0.06 | 2166 | -0.22 |
| K407 1298 _ 02 corrected | 4449 | 0.00 | 849014 | 48.46 | 5989 | 0.14 | 5195 | 0.00 |
| K407 1298 _ 03 corrected | 676 | -0.25 | 147509 | 8.11 | 2072 | -0.09 | 2012 | -0.23 |
| K407 1298 _ 04 corrected | 771 | -0.24 | 51857 | 2.61 | 1349 | -0.13 | 2099 | -0.22 |
| K407 1298 _ 05 | 1509 | -0.19 | 222935 | 12.45 | 2729 | -0.05 | 3478 | -0.12 |
| K407 1298 paper | 1229 | -0.21 | 4555 | -0.11 | 1878 | -0.10 | 1577 | -0.26 |
| K408 1556 _ 01 | 2957 | -0.10 | 411492 | 23.29 | 2385 | -0.07 | 1632 | -0.25 |
| K408 1556 _ 02 | 2687 | -0.11 | 353427 | 19.95 | 2428 | -0.07 | 2212 | -0.21 |
| K408 1556 _ 03 | 1291 | -0.21 | 114007 | 6.18 | 2103 | -0.09 | 2368 | -0.20 |
| K408 1556 _ 04 | 1618 | -0.18 | 174265 | 9.65 | 4229 | 0.04 | 1224 | -0.28 |
| K408 1556 _ 05 | 1341 | -0.20 | 121854 | 6.63 | 3937 | 0.02 | 2771 | -0.17 |
| K408 1556 _ 06 | 1336 | -0.20 | 189604 | 10.53 | 4926 | 0.08 | 2868 | -0.17 |
| K408 1556 paper | 641 | -0.25 | 3194 | -0.19 | 2205 | -0.08 | 2624 | -0.18 |
| K409 1608 _ 01 corrected | 997 | -0.22 | 261086 | 14.64 | 38552 | 2.04 | 4479 | -0.05 |
| K409 1608 _ 03 | 1091 | -0.22 | 61749 | 3.18 | 10027 | 0.38 | 2641 | -0.18 |
| K409 1608 _ 04 | 760 | -0.24 | 39104 | 1.87 | 6611 | 0.18 | 2398 | -0.20 |
| K409 1608 _ 05 | 2069 | -0.15 | 282372 | 15.87 | 39586 | 2.10 | 5404 | 0.01 |
| K409 1608 paper | 977 | -0.23 | 4361 | -0.13 | 2404 | -0.07 | 2587 | -0.19 |
| K412 1902 _ 01 | 5413 | 0.07 | 477368 | 27.08 | 1476 | -0.12 | 14712 | 0.66 |
| K412 1902 _ 02 corrected | 3104 | -0.09 | 221792 | 12.38 | 884 | -0.16 | 12218 | 0.49 |
| K412 1902 _ 03 | 3613 | -0.05 | 279980 | 15.73 | 986 | -0.15 | 11464 | 0.44 |
| K412 1902 _ 04 | 6126 | 0.11 | 502839 | 28.55 | 1189 | -0.14 | 14085 | 0.62 |
| K412 1902 paper corrected | 1218 | -0.21 | 4896 | -0.10 | 1914 | -0.10 | 7702 | 0.17 |
| K415 2098 _ 01 | 1847 | -0.17 | 194708 | 10.82 | 9019 | 0.32 | 3224 | -0.14 |
| K415 2098 _ 02 corrected | 2101 | -0.15 | 144320 | 7.93 | 8579 | 0.29 | 3931 | -0.09 |
| K415 2098 _ 03 | 853 | -0.23 | 135832 | 7.44 | 1924 | -0.10 | 2435 | -0.20 |
| K415 2098 _ 04 | 983 | -0.23 | 84589 | 4.49 | 4530 | 0.06 | 3039 | -0.16 |
| K415 2098 paper | 756 | -0.24 | 3715 | -0.16 | 1070 | -0.15 | 618 | -0.33 |
| K415 2182 _ 01 | 1426 | -0.20 | 149245 | 8.21 | 2831 | -0.04 | 2641 | -0.18 |
| K415 2182 _ 02 corrected | 3956 | -0.03 | 288683 | 16.23 | 3147 | -0.02 | 1399 | -0.27 |
| K415 2182 _ 03 | 853 | -0.23 | 135832 | 7.44 | 1924 | -0.10 | 2435 | -0.20 |
| K415 2182 _ 04 | 1116 | -0.22 | 117652 | 6.39 | 2049 | -0.09 | 1967 | -0.23 |
| K415 2182 _ 05 | 869 | -0.23 | 54581 | 2.76 | 2871 | -0.04 | 2944 | -0.16 |
| K415 2182 paper | 781 | -0.24 | 3017 | -0.20 | 1120 | -0.14 | 835 | -0.31 |
| K419 2574 _ 01 | 679 | -0.25 | 23161 | 0.96 | 963 | -0.15 | 2709 | -0.18 |
| K419 2574 _ 02 | 597 | -0.25 | 38467 | 1.84 | 902 | -0.16 | 2546 | -0.19 |
| K419 2574 _ 03 | 910 | -0.23 | 50838 | 2.55 | 1245 | -0.14 | 3233 | -0.14 |
| K419 2574 _ 04 | 1074 | -0.22 | 47962 | 2.38 | 1158 | -0.14 | 2972 | -0.16 |
| K419 2574 _ 05 | 1526 | -0.19 | 30315 | 1.37 | 1528 | -0.12 | 4836 | -0.03 |
| K419 2574 paper | 675 | -0.25 | 3006 | -0.20 | 1336 | -0.13 | 1913 | -0.23 |
| K420 2868 _ 01 | 824 | -0.24 | 215103 | 12.00 | 311 | -0.19 | 633 | -0.32 |
| K420 2868 _ 02 | 1121 | -0.22 | 286572 | 16.11 | 2007 | -0.09 | 1746 | -0.25 |
| K420 2868 _ 03 | 864 | -0.23 | 158908 | 8.76 | 2296 | -0.07 | 1441 | -0.27 |
| K420 2868 _ 04 corrected | 1474 | -0.19 | 111382 | 6.03 | 2033 | -0.09 | 719 | -0.32 |
| K420 2868 paper | 479 | -0.26 | 3827 | -0.16 | 1051 | -0.15 | 1658 | -0.25 |
| K421 3064 _ 01 | 585 | -0.25 | 87763 | 4.67 | 1203 | -0.14 | 2382 | -0.20 |
| K421 3064 _ 02 | 744 | -0.24 | 164205 | 9.07 | 1078 | -0.15 | 1961 | -0.23 |
| K421 3064 _ 03 | 969 | -0.23 | 76615 | 4.03 | 1695 | -0.11 | 1666 | -0.25 |
| K421 3064 _ 04 | 1190 | -0.21 | 48194 | 2.40 | 1992 | -0.09 | 2089 | -0.22 |
| K421 3064 _ 05 | 453 | -0.26 | 142998 | 7.85 | 1777 | -0.10 | 1560 | -0.26 |
| K421 3064 _ 06 | 917 | -0.23 | 49619 | 2.48 | 1397 | -0.13 | 2190 | -0.22 |
| K421 3064 paper | 963 | -0.23 | 5355 | -0.07 | 1306 | -0.13 | 1263 | -0.28 |
| K422 3078 _ 01 | 1049 | -0.22 | 310730 | 17.50 | 3736 | 0.01 | 1403 | -0.27 |
| K422 3078 _ 02 | 775 | -0.24 | 190426 | 10.58 | 2775 | -0.05 | 2112 | -0.22 |
| K422 3078 _ 03 | 471 | -0.26 | 85700 | 4.55 | 5341 | 0.10 | 5369 | 0.01 |
| K422 3078 _ 04 | 828 | -0.24 | 62811 | 3.24 | 4020 | 0.03 | 4077 | -0.08 |
| K422 3190 _ 01 | 1246 | -0.21 | 410731 | 23.25 | 3125 | -0.03 | 1765 | -0.24 |
| K422 3190 _ 02 | 1473 | -0.19 | 321735 | 18.13 | 2737 | -0.05 | 2261 | -0.21 |
| K422 3190 _ 03 corrected | 2111 | -0.15 | 409579 | 23.18 | 3420 | -0.01 | 2058 | -0.22 |
| K422 3190 _ 05 | 228 | -0.28 | 100522 | 5.41 | 18855 | 0.89 | 7662 | 0.17 |
| K422 3190 _ 06 | 279 | -0.27 | 80923 | 4.28 | 16985 | 0.78 | 7567 | 0.16 |
| K423 3234 _ 01 | 1166 | -0.21 | 153491 | 8.45 | 1687 | -0.11 | 1624 | -0.25 |
| K423 3234 _ 02 | 766 | -0.24 | 44706 | 2.20 | 1264 | -0.13 | 928 | -0.30 |
| K423 3234 _ 03 | 2041 | -0.16 | 212455 | 11.85 | 795 | -0.16 | 2070 | -0.22 |
| K423 3234 _ 04 | 777 | -0.24 | 128147 | 7.00 | 1332 | -0.13 | 1700 | -0.25 |
| K423 3234 paper | 896 | -0.23 | 3318 | -0.19 | 994 | -0.15 | 2083 | -0.22 |
| K429 3767 _ 01 | 759 | -0.24 | 228760 | 12.78 | 4594 | 0.06 | 2421 | -0.20 |
| K429 3767 _ 02 | 450 | -0.26 | 168176 | 9.30 | 3484 | -0.01 | 1871 | -0.24 |
| K429 3767 _ 03 | 1164 | -0.21 | 278802 | 15.66 | 5138 | 0.09 | 2629 | -0.18 |
| K429 3767 _ 04 | 621 | -0.25 | 275512 | 15.47 | 4358 | 0.05 | 2682 | -0.18 |
| K429 3767 _ 05 | 766 | -0.24 | 29502 | 1.32 | 1673 | -0.11 | 1930 | -0.23 |
| K429 3767 paper | 812 | -0.24 | 2820 | -0.21 | 953 | -0.15 | 198 | -0.35 |
| K429 3800 _ 01 | 1836 | -0.17 | 270346 | 15.18 | 2282 | -0.08 | 1973 | -0.23 |
| K429 3800 _ 02 | 353 | -0.27 | 75665 | 3.98 | 1362 | -0.13 | 1626 | -0.25 |
| K429 3800 _ 03 | 1317 | -0.20 | 174478 | 9.66 | 2230 | -0.08 | 2333 | -0.20 |
| K429 3800 _ 04 | 2157 | -0.15 | 249257 | 13.96 | 2600 | -0.06 | 2169 | -0.22 |
| K429 3800 _ 05 | 417 | -0.26 | 48846 | 2.43 | 1306 | -0.13 | 2022 | -0.23 |
| K429 3800 _ 06 | 293 | -0.27 | 30551 | 1.38 | 1519 | -0.12 | 1886 | -0.24 |
| K429 3800 paper corrected | 716 | -0.24 | 3928 | -0.15 | 1274 | -0.13 | 1868 | -0.24 |
| K429 3801 _ 01 | 1017 | -0.22 | 340306 | 19.20 | 1681 | -0.11 | 2064 | -0.22 |
| K429 3801 _ 02 | 140 | -0.28 | 43064 | 2.10 | 626 | -0.17 | 1983 | -0.23 |
| K429 3801 _ 03 | 548 | -0.25 | 203560 | 11.33 | 1349 | -0.13 | 1684 | -0.25 |
| K429 3801 _ 04 | -2 | -0.29 | 23344 | 0.97 | 1215 | -0.14 | 1729 | -0.25 |
| K429 3801 _ 05 | 110 | -0.28 | 130257 | 7.12 | 1545 | -0.12 | 1512 | -0.26 |
| K429 3801 paper corrected | 360 | -0.27 | 2804 | -0.22 | 1555 | -0.12 | 1475 | -0.27 |
| K430 3842 part 1 _ 01 | 909 | -0.23 | 240456 | 13.46 | 3539 | 0.00 | 1205 | -0.28 |
| K430 3842 part 1 _ 02 | 1182 | -0.21 | 422861 | 23.95 | 6271 | 0.16 | 936 | -0.30 |
| K430 3842 part 1 _ 03 | 1737 | -0.18 | 446842 | 25.33 | 8345 | 0.28 | 2268 | -0.21 |
| K430 3842 part 1 _ 04 corrected | 333 | -0.27 | 136440 | 7.47 | 2380 | -0.07 | 1650 | -0.25 |
| K430 3842 part 1 _ 05 corrected | 583 | -0.25 | 92621 | 4.95 | 1637 | -0.11 | 987 | -0.30 |
| K430 3842 part 1 _ 06 | 522 | -0.26 | 99186 | 5.33 | 1869 | -0.10 | 1208 | -0.28 |
| K430 3842 part 1 paper | 510 | -0.26 | 3511 | -0.17 | 1247 | -0.14 | 1719 | -0.25 |
|  |  |  |  |  |  |  |  |  |
| MAX | 6126 | 0.11 | 849014 | 48.46 | 39586 | 2.10 | 23393 | 1.27 |
| MIN | -2 | -0.29 | 2804 | -0.22 | 311 | -0.19 | 198 | -0.35 |

## Compton ratios quantification results for Azzolino collection analyses

| Mn quantification linear regression | Fe quantification linear regression | Cu quantification linear regression | Zn quantification linear regression |
| --- | --- | --- | --- |
| y = 0.0053970068x - 0.4830118483 | y = 0.0048044480x - 0.4587093236 | y = 0.0047860850x - 0.2453681408 | y = 0.0058732263x - 0.3008625924 |
| R² = 0.9876043400 | R² = 0.9988929449 | R² = 0.9987489891 | R² = 0.9973044119 |

### Compton ratios and concentration metal/paper (mg/g)

| Spectrum | Mn Compton ratio | Mn concentration metal/paper (mg/g) | Fe Compton ratio | Fe concentration metal/paper (mg/g) | Cu Compton ratio | Cu concentration metal/paper (mg/g) | Zn Compton ratio | Zn concentration metal/paper (mg/g) |
| --- | --- | --- | --- | --- | --- | --- | --- | --- |
| Del Monte Vol II A1 No 17 line 01 | 8.90 | -0.43 | 789.44 | 3.33 | 12.03 | -0.19 | 19.96 | -0.18 |
| Del Monte Vol II A1 No 17 line 02 | 8.90 | -0.43 | 901.43 | 3.87 | 9.81 | -0.20 | 18.53 | -0.19 |
| Del Monte Vol II A1 No 17 line 03 | 7.07 | -0.44 | 893.68 | 3.83 | 9.53 | -0.20 | 16.18 | -0.21 |
| Del Monte Vol II A1 No 17 line 04 | 9.48 | -0.43 | 1360.57 | 6.08 | 7.92 | -0.21 | 20.42 | -0.18 |
| Del Monte Vol II A1 No 17 paper | 5.03 | -0.46 | 19.99 | -0.36 | 9.80 | -0.20 | 13.87 | -0.22 |
| K394 0014 line 01 | 16.49 | -0.39 | 408.08 | 1.50 | 44.03 | -0.03 | 59.29 | 0.05 |
| K394 0014 line 02 | 30.80 | -0.32 | 1338.06 | 5.97 | 133.28 | 0.39 | 147.48 | 0.57 |
| K394 0014 line 03 | 32.75 | -0.31 | 247.98 | 0.73 | 26.14 | -0.12 | 41.02 | -0.06 |
| K394 0014 line 04 | 15.73 | -0.40 | 396.21 | 1.44 | 40.02 | -0.05 | 53.45 | 0.01 |
| K394 0014 line 05 | 18.94 | -0.38 | 554.20 | 2.20 | 59.50 | 0.04 | 85.16 | 0.20 |
| K394 0014 paper | 284.57 | 1.05 | 696.97 | 2.89 | 43.85 | -0.04 | 55.80 | 0.03 |
| K396 266 line 01 | 16.58 | -0.39 | 2544.48 | 11.77 | 25.92 | -0.12 | 16.62 | -0.20 |
| K396 266 line 02 | 5.12 | -0.46 | 708.99 | 2.95 | 11.49 | -0.19 | 14.05 | -0.22 |
| K396 266 line 03 | 7.41 | -0.44 | 1088.63 | 4.77 | 16.42 | -0.17 | 11.94 | -0.23 |
| K396 266 line 04 | 7.02 | -0.45 | 1453.23 | 6.52 | 19.59 | -0.15 | 14.70 | -0.21 |
| K396 266 line 05 corrected | 23.98 | -0.35 | 3562.04 | 16.65 | 65.31 | 0.07 | 19.87 | -0.18 |
| K396 266 paper | 5.24 | -0.45 | 33.45 | -0.30 | 11.93 | -0.19 | 21.03 | -0.18 |
| K397 364 line 01 | 25.23 | -0.35 | 1132.08 | 4.98 | 11.06 | -0.19 | 32.77 | -0.11 |
| K397 364 line 02 | 21.09 | -0.37 | 769.36 | 3.24 | 11.18 | -0.19 | 23.49 | -0.16 |
| K397 364 line 03 | 21.90 | -0.36 | 986.30 | 4.28 | 9.39 | -0.20 | 26.81 | -0.14 |
| K397 364 line 04 | 14.87 | -0.40 | 561.00 | 2.24 | 8.10 | -0.21 | 18.30 | -0.19 |
| K397 364 line 05 | 13.65 | -0.41 | 615.26 | 2.50 | 5.62 | -0.22 | 14.87 | -0.21 |
| K397 364 paper | 12.97 | -0.41 | 35.18 | -0.29 | 13.08 | -0.18 | 15.42 | -0.21 |
| K399 470 line 01 | 10.18 | -0.43 | 733.94 | 3.07 | 12.64 | -0.18 | 16.56 | -0.20 |
| K399 470 line 02 | 10.33 | -0.43 | 1247.51 | 5.53 | 21.05 | -0.14 | 27.06 | -0.14 |
| K399 470 line 03 | 3.55 | -0.46 | 396.88 | 1.45 | 9.72 | -0.20 | 17.46 | -0.20 |
| K399 470 line 04 | 10.59 | -0.43 | 877.23 | 3.76 | 16.20 | -0.17 | 20.60 | -0.18 |
| K399 470 line 05 | 7.32 | -0.44 | 516.10 | 2.02 | 15.51 | -0.17 | 18.58 | -0.19 |
| K399 470 paper | 3.15 | -0.47 | 29.00 | -0.32 | 13.14 | -0.18 | 14.55 | -0.22 |
| K401 658 line 01 | 41.85 | -0.26 | 3569.22 | 16.69 | 44.30 | -0.03 | 56.46 | 0.03 |
| K401 658 line 02 | 39.21 | -0.27 | 3177.35 | 14.81 | 47.39 | -0.02 | 56.54 | 0.03 |
| K401 658 line 03 | 14.26 | -0.41 | 1184.95 | 5.23 | 15.88 | -0.17 | 26.95 | -0.14 |
| K401 658 line 04 | 22.43 | -0.36 | 2168.40 | 9.96 | 30.03 | -0.10 | 38.01 | -0.08 |
| K401 658 line 05 | 14.13 | -0.41 | 908.36 | 3.91 | 18.29 | -0.16 | 23.06 | -0.17 |
| K401 658 paper | 9.69 | -0.43 | 37.59 | -0.28 | 11.58 | -0.19 | 16.79 | -0.20 |
| K403 828 line 01 | 4.34 | -0.46 | 415.10 | 1.54 | 10.40 | -0.20 | 18.82 | -0.19 |
| K403 828 line 02 | 3.35 | -0.46 | 228.06 | 0.64 | 10.53 | -0.19 | 16.73 | -0.20 |
| K403 828 line 03 | 5.82 | -0.45 | 727.08 | 3.03 | 8.43 | -0.21 | 15.56 | -0.21 |
| K403 828 line 04 | 8.42 | -0.44 | 207.08 | 0.54 | 13.92 | -0.18 | 15.42 | -0.21 |
| K403 828 line 05 | 5.60 | -0.45 | 552.68 | 2.20 | 6.97 | -0.21 | 14.80 | -0.21 |
| K403 828 line 06 | 5.68 | -0.45 | 434.98 | 1.63 | 7.34 | -0.21 | 15.96 | -0.21 |
| K403 828 paper | 4.19 | -0.46 | 25.48 | -0.34 | 10.86 | -0.19 | 16.75 | -0.20 |
| K405 1092 line 01 | 7.39 | -0.44 | 314.54 | 1.05 | 10.13 | -0.20 | 16.10 | -0.21 |
| K405 1092 line 02 corrected | 8.88 | -0.44 | 407.90 | 1.50 | 14.94 | -0.17 | 19.34 | -0.19 |
| K405 1092 line 03 | 7.28 | -0.44 | 354.02 | 1.24 | 8.49 | -0.20 | 14.98 | -0.21 |
| K405 1092 line 04 | 3.00 | -0.47 | 123.77 | 0.14 | 8.01 | -0.21 | 15.51 | -0.21 |
| K405 1092 line 05 | 6.69 | -0.45 | 382.12 | 1.38 | 11.22 | -0.19 | 16.23 | -0.21 |
| K405 1092 paper | 4.41 | -0.46 | 18.48 | -0.37 | 8.91 | -0.20 | 14.44 | -0.22 |
| K407 1298 line 01 corrected | 4.54 | -0.46 | 1344.87 | 6.00 | 15.03 | -0.17 | 12.50 | -0.23 |
| K407 1298 line 02 corrected | 28.11 | -0.33 | 5365.22 | 25.32 | 37.85 | -0.06 | 32.83 | -0.11 |
| K407 1298 line 03 corrected | 4.12 | -0.46 | 898.91 | 3.86 | 12.63 | -0.18 | 12.26 | -0.23 |
| K407 1298 line 04 corrected | 4.51 | -0.46 | 303.55 | 1.00 | 7.90 | -0.21 | 12.29 | -0.23 |
| K407 1298 line 05 | 7.54 | -0.44 | 1113.29 | 4.89 | 13.63 | -0.18 | 17.37 | -0.20 |
| K407 1298 paper | 12.07 | -0.42 | 44.73 | -0.24 | 18.44 | -0.16 | 15.49 | -0.21 |
| K408 1556 line 01 | 28.85 | -0.33 | 4014.87 | 18.83 | 23.27 | -0.13 | 15.92 | -0.21 |
| K408 1556 line 02 | 20.93 | -0.37 | 2752.53 | 12.77 | 18.91 | -0.15 | 17.23 | -0.20 |
| K408 1556 line 03 | 9.99 | -0.43 | 882.26 | 3.78 | 16.27 | -0.17 | 18.33 | -0.19 |
| K408 1556 line 04 | 28.50 | -0.33 | 3069.67 | 14.29 | 74.49 | 0.11 | 21.56 | -0.17 |
| K408 1556 line 05 | 7.26 | -0.44 | 659.58 | 2.71 | 21.31 | -0.14 | 15.00 | -0.21 |
| K408 1556 line 06 | 6.44 | -0.45 | 914.30 | 3.93 | 23.75 | -0.13 | 13.83 | -0.22 |
| K408 1556 paper | 3.70 | -0.46 | 18.44 | -0.37 | 12.73 | -0.18 | 15.15 | -0.21 |
| K409 1608 line 01 corrected | 5.43 | -0.45 | 1422.89 | 6.38 | 210.10 | 0.76 | 24.41 | -0.16 |
| K409 1608 line 03 | 7.25 | -0.44 | 410.24 | 1.51 | 66.62 | 0.07 | 17.55 | -0.20 |
| K409 1608 line 04 | 3.97 | -0.46 | 204.42 | 0.52 | 34.56 | -0.08 | 12.54 | -0.23 |
| K409 1608 line 05 | 12.23 | -0.42 | 1669.66 | 7.56 | 234.07 | 0.87 | 31.95 | -0.11 |
| K409 1608 paper | 7.40 | -0.44 | 33.04 | -0.30 | 18.22 | -0.16 | 19.60 | -0.19 |
| K412 1902 line 01 | 26.74 | -0.34 | 2357.80 | 10.87 | 7.29 | -0.21 | 72.67 | 0.13 |
| K412 1902 line 02 corrected | 35.56 | -0.29 | 2540.92 | 11.75 | 10.13 | -0.20 | 139.97 | 0.52 |
| K412 1902 line 03 | 22.37 | -0.36 | 1733.57 | 7.87 | 6.11 | -0.22 | 70.98 | 0.12 |
| K412 1902 line 04 | 40.63 | -0.26 | 3334.81 | 15.56 | 7.89 | -0.21 | 93.41 | 0.25 |
| K412 1902 paper corrected | 7.54 | -0.44 | 30.30 | -0.31 | 11.85 | -0.19 | 47.67 | -0.02 |
| K415 2098 line 01 | 18.65 | -0.38 | 1965.75 | 8.99 | 91.06 | 0.19 | 32.55 | -0.11 |
| K415 2098 line 02 corrected | 11.47 | -0.42 | 787.80 | 3.33 | 46.83 | -0.02 | 21.46 | -0.17 |
| K415 2098 line 03 | 5.34 | -0.45 | 850.66 | 3.63 | 12.05 | -0.19 | 15.25 | -0.21 |
| K415 2098 line 04 | 5.83 | -0.45 | 502.09 | 1.95 | 26.89 | -0.12 | 18.04 | -0.19 |
| K415 2098 paper | 20.25 | -0.37 | 99.50 | 0.02 | 28.66 | -0.11 | 16.55 | -0.20 |
| K415 2182 line 01 | 9.38 | -0.43 | 982.23 | 4.26 | 18.63 | -0.16 | 17.38 | -0.20 |
| K415 2182 line 02 corrected | 130.53 | 0.22 | 9524.98 | 45.30 | 103.83 | 0.25 | 46.16 | -0.03 |
| K415 2182 line 03 | 5.34 | -0.45 | 850.66 | 3.63 | 12.05 | -0.19 | 15.25 | -0.21 |
| K415 2182 line 04 | 12.07 | -0.42 | 1272.13 | 5.65 | 22.16 | -0.14 | 21.27 | -0.18 |
| K415 2182 line 05 | 5.09 | -0.46 | 319.91 | 1.08 | 16.83 | -0.16 | 17.26 | -0.20 |
| K415 2182 paper | 11.20 | -0.42 | 43.26 | -0.25 | 16.06 | -0.17 | 11.97 | -0.23 |
| K419 2574 line 01 | 3.70 | -0.46 | 126.09 | 0.15 | 5.24 | -0.22 | 14.75 | -0.21 |
| K419 2574 line 02 | 4.40 | -0.46 | 283.42 | 0.90 | 6.65 | -0.21 | 18.76 | -0.19 |
| K419 2574 line 03 | 6.01 | -0.45 | 335.96 | 1.16 | 8.23 | -0.21 | 21.37 | -0.18 |
| K419 2574 line 04 | 12.10 | -0.42 | 540.51 | 2.14 | 13.05 | -0.18 | 33.49 | -0.10 |
| K419 2574 line 05 | 9.00 | -0.43 | 178.76 | 0.40 | 9.01 | -0.20 | 28.52 | -0.13 |
| K419 2574 paper | 4.07 | -0.46 | 18.14 | -0.37 | 8.06 | -0.21 | 11.54 | -0.23 |
| K420 2868 line 01 | 19.65 | -0.38 | 5129.56 | 24.19 | 7.42 | -0.21 | 15.10 | -0.21 |
| K420 2868 line 02 | 6.81 | -0.45 | 1739.89 | 7.90 | 12.19 | -0.19 | 10.60 | -0.24 |
| K420 2868 line 03 | 4.72 | -0.46 | 867.93 | 3.71 | 12.54 | -0.19 | 7.87 | -0.25 |
| K420 2868 line 04 corrected | 24.57 | -0.35 | 1856.83 | 8.46 | 33.89 | -0.08 | 11.99 | -0.23 |
| K420 2868 paper | 2.81 | -0.47 | 22.44 | -0.35 | 6.16 | -0.22 | 9.72 | -0.24 |
| K421 3064 line 01 | 3.49 | -0.46 | 524.01 | 2.06 | 7.18 | -0.21 | 14.22 | -0.22 |
| K421 3064 line 02 | 4.22 | -0.46 | 931.07 | 4.01 | 6.11 | -0.22 | 11.12 | -0.24 |
| K421 3064 line 03 | 5.42 | -0.45 | 428.72 | 1.60 | 9.48 | -0.20 | 9.32 | -0.25 |
| K421 3064 line 04 | 7.03 | -0.45 | 284.65 | 0.91 | 11.77 | -0.19 | 12.34 | -0.23 |
| K421 3064 line 05 | 3.58 | -0.46 | 1131.22 | 4.98 | 14.06 | -0.18 | 12.34 | -0.23 |
| K421 3064 line 06 | 5.21 | -0.45 | 282.09 | 0.90 | 7.94 | -0.21 | 12.45 | -0.23 |
| K421 3064 paper | 10.27 | -0.43 | 57.09 | -0.18 | 13.92 | -0.18 | 13.46 | -0.22 |
| K422 3078 line 01 | 8.90 | -0.43 | 2635.83 | 12.20 | 31.69 | -0.09 | 11.90 | -0.23 |
| K422 3078 line 02 | 4.99 | -0.46 | 1226.24 | 5.43 | 17.87 | -0.16 | 13.60 | -0.22 |
| K422 3078 line 03 | 4.69 | -0.46 | 852.97 | 3.64 | 53.16 | 0.01 | 53.44 | 0.01 |
| K422 3078 line 04 | 9.74 | -0.43 | 739.14 | 3.09 | 47.31 | -0.02 | 47.98 | -0.02 |
| K422 3190 line 01 | 9.82 | -0.43 | 3235.94 | 15.09 | 24.62 | -0.13 | 13.91 | -0.22 |
| K422 3190 line 02 | 10.28 | -0.43 | 2245.34 | 10.33 | 19.10 | -0.15 | 15.78 | -0.21 |
| K422 3190 line 03 corrected | 22.29 | -0.36 | 4324.15 | 20.32 | 36.11 | -0.07 | 21.73 | -0.17 |
| K422 3190 line 05 | 1.69 | -0.47 | 744.88 | 3.12 | 139.72 | 0.42 | 56.78 | 0.03 |
| K422 3190 line 06 | 1.80 | -0.47 | 521.19 | 2.05 | 109.39 | 0.28 | 48.74 | -0.01 |
| K423 3234 line 01 | 15.24 | -0.40 | 2006.39 | 9.18 | 22.05 | -0.14 | 21.23 | -0.18 |
| K423 3234 line 02 | 10.94 | -0.42 | 638.30 | 2.61 | 18.05 | -0.16 | 13.25 | -0.22 |
| K423 3234 line 03 | 16.91 | -0.39 | 1760.35 | 8.00 | 6.59 | -0.21 | 17.15 | -0.20 |
| K423 3234 line 04 | 4.83 | -0.46 | 796.30 | 3.37 | 8.28 | -0.21 | 10.56 | -0.24 |
| K423 3234 paper | 6.64 | -0.45 | 24.57 | -0.34 | 7.36 | -0.21 | 15.43 | -0.21 |
| K429 3767 line 01 | 5.37 | -0.45 | 1617.52 | 7.31 | 32.48 | -0.09 | 17.12 | -0.20 |
| K429 3767 line 02 | 3.28 | -0.47 | 1225.61 | 5.43 | 25.39 | -0.12 | 13.64 | -0.22 |
| K429 3767 line 03 | 9.19 | -0.43 | 2200.66 | 10.11 | 40.56 | -0.05 | 20.75 | -0.18 |
| K429 3767 line 04 | 3.72 | -0.46 | 1648.99 | 7.46 | 26.08 | -0.12 | 16.05 | -0.21 |
| K429 3767 line 05 | 4.78 | -0.46 | 184.28 | 0.43 | 10.45 | -0.20 | 12.06 | -0.23 |
| K429 3767 paper | 33.32 | -0.30 | 115.72 | 0.10 | 39.11 | -0.06 | 8.13 | -0.25 |
| K429 3800 line 01 | 11.96 | -0.42 | 1761.45 | 8.00 | 14.87 | -0.17 | 12.86 | -0.23 |
| K429 3800 line 02 | 2.28 | -0.47 | 489.30 | 1.89 | 8.81 | -0.20 | 10.51 | -0.24 |
| K429 3800 line 03 | 7.65 | -0.44 | 1013.63 | 4.41 | 12.96 | -0.18 | 13.55 | -0.22 |
| K429 3800 line 04 | 14.61 | -0.40 | 1688.76 | 7.65 | 17.62 | -0.16 | 14.70 | -0.21 |
| K429 3800 line 05 | 2.38 | -0.47 | 278.75 | 0.88 | 7.45 | -0.21 | 11.54 | -0.23 |
| K429 3800 line 06 | 1.66 | -0.47 | 173.56 | 0.38 | 8.63 | -0.20 | 10.71 | -0.24 |
| K429 3800 paper corrected | 4.85 | -0.46 | 26.62 | -0.33 | 8.63 | -0.20 | 12.66 | -0.23 |
| K429 3801 line 01 | 6.12 | -0.45 | 2047.06 | 9.38 | 10.11 | -0.20 | 12.42 | -0.23 |
| K429 3801 line 02 | 0.82 | -0.48 | 252.89 | 0.76 | 3.68 | -0.23 | 11.64 | -0.23 |
| K429 3801 line 03 | 3.63 | -0.46 | 1349.02 | 6.02 | 8.94 | -0.20 | 11.16 | -0.24 |
| K429 3801 line 04 | -0.01 | -0.48 | 144.56 | 0.24 | 7.52 | -0.21 | 10.71 | -0.24 |
| K429 3801 line 05 | 0.87 | -0.48 | 1032.30 | 4.50 | 12.24 | -0.19 | 11.98 | -0.23 |
| K429 3801 paper corrected | 2.42 | -0.47 | 18.85 | -0.37 | 10.46 | -0.20 | 9.92 | -0.24 |
| K430 3842 part 1 line 01 | 12.83 | -0.41 | 3393.83 | 15.85 | 49.95 | -0.01 | 17.01 | -0.20 |
| K430 3842 part 1 line 02 | 31.20 | -0.31 | 11161.70 | 53.17 | 165.53 | 0.55 | 24.71 | -0.16 |
| K430 3842 part 1 line 03 | 10.30 | -0.43 | 2648.60 | 12.27 | 49.46 | -0.01 | 13.44 | -0.22 |
| K430 3842 part 1 line 04 corrected | 2.22 | -0.47 | 909.07 | 3.91 | 15.86 | -0.17 | 10.99 | -0.24 |
| K430 3842 part 1 line 05 corrected | 6.39 | -0.45 | 1015.73 | 4.42 | 17.95 | -0.16 | 10.82 | -0.24 |
| K430 3842 part 1 line 06 | 6.97 | -0.45 | 1325.06 | 5.91 | 24.97 | -0.13 | 16.14 | -0.21 |
| K430 3842 part 1 paper | 4.63 | -0.46 | 31.90 | -0.31 | 11.33 | -0.19 | 15.62 | -0.21 |
|  |  |  |  |  |  |  |  |  |
| MAX | 284.57 | 1.05 | 11161.70 | 53.17 | 234.07 | 0.87 | 147.48 | 0.57 |
| MIN | -0.01 | -0.48 | 18.14 | -0.37 | 3.68 | -0.23 | 7.87 | -0.25 |

## Results of ink ratios based on net peak quantification

Table showing weight/weight ratios of Mn, Cu, Zn in relation to Fe for all letters measured (+ means that peaks are present but net peak area values gave low quality regression lines with R^2^ < 0.8 or only two points were measured, hence regression lines would be invalid. Blank means that no peaks were present in the XRF spectra.)

| **Letter** | Slope for net area Mn versus net area Fe | Slope (Amn/Afe) * Slope concMn / Slope concFe | Slope for net area Cu versus net area Fe | Slope (Acu/Afe) * Slope concCu / Slope concFe | Slope for net area Zn versus net area Fe | Slope (Azn/Afe) * Slope concZn / Slope concFe |
| --- | --- | --- | --- | --- | --- | --- |
|  | **w/w ratio Mn/Fe** | | **w/w ratio Cu/Fe** | | **w/w ratio Zn/Fe** | |
| Del Monte Vol II A1 No 17 | y = 0,00227712x + 1 192,84689644 | + |  |  |  |  |
|  | R² = 0,47240288 |  |  |  |  |  |
| K394 0014 | y = 0,01086667x + 2 490,55747936 | 0.012 | y = 0.09868903x + 437.66663978 | 0.100 | y = 0,10269830x + 2 890,12717132 | 0.125 |
|  | R² = 0,90153795 |  | R² = 0.99714778 |  | R² = 0,94599149 |  |
| K396 266 | y = 0,00645266x - 12,38450015 | 0.007 | y = 0,02066182x - 1 131,87939464 | + |  |  |
|  | R² = 0,81229091 |  | R² = 0,77403251 |  |  |  |
| K397 364 | y = 0,01796481x + 832,63690144 | 0.021 |  |  | y = 0,02657016x + 289,35562832 | 0.032 |
|  | R² = 0,86199982 |  |  |  | R² = 0,88331112 |  |
| K399 470 | y = 0,01267833x - 102,17586869 | + |  |  |  |  |
|  | R² = 0,57037864 |  |  |  |  |  |
| K401 658 | y = 0,00661330x + 1 337,27843889 | 0.008 | y = 0,01084601x + 932,52974424 | + | y = 0,01309870x + 1 556,11605543 | + |
|  | R² = 0,87784552 |  | R² = 0,71992727 |  | R² = 0,65541233 |  |
| K403 828 Azzolino | y = 0.00428135x + 448.81068670 | 0.005 |  |  |  |  |
|  | R² = 0.98065031 |  |  |  |  |  |
| K403 828 Christina | y = 0.0034x + 798.41 | + |  |  |  |  |
|  | R² = 0.5532 |  |  |  |  |  |
| K405 1092 | y = 0,01829997x + 140,27027730 | 0.021 |  |  |  |  |
|  | R² = 0,87604936 |  |  |  |  |  |
| K407 1298 | y = 0,00497173x + 142,34262076 | 0.006 | y = 0,00555029x + 1 280,50588690 | 0.006 | y = 0,00467870x + 1 206,73549844 | 0.006 |
|  | R² = 0,94994196 |  | R² = 0,99980981 |  | R² = 0,99523626 |  |
| K408 1556 | y = 0,00566736x + 651,27022789 | 0.006 | y = 0,00106713x + 1 992,69156670 | 0.001 |  |  |
|  | R² = 0,99887050 |  | R² = 0,90852520 |  |  |  |
| K408 1556 | y = 0,00130126x + 1 220,98227924 | + | y = 0,01228168x + 2 375,50256254 | + |  |  |
|  | R² = 0,08204603 |  | R² = 0,73728428 |  |  |  |
| K409 1608 | y = 0,00302477x + 742,02678983 | + | y = 0,13859780x + 1 368,97742745 | 0.141 | y = 0,01109211x + 1 943,80758743 | 0.014 |
|  | R² = 0,45321266 |  | R² = 0,99802458 |  | R² = 0,96230038 |  |
| K412 1902 | y = 0,01013874x + 807,64898553 | 0.012 |  |  | y = 0,00982198x + 9 480,75897257 | 0.012 |
|  | R² = 0,98275122 |  |  |  | R² = 0,81393935 |  |
| K415 2098 | y = 0,00867221x + 233,08544179 | + | y = 0,04504692x - 287,36410991 | + |  |  |
|  | R² = 0,39686278 |  | R² = 0,35833823 |  |  |  |
| K415 2182 | y = 0,01430034x - 489,59016824 | 0.016 | y = 0,00260580x + 2 175,61878298 | + |  |  |
|  | R² = 0,87700704 |  | R² = 0,17019144 |  |  |  |
| K419 2574 | y = 0,00026936x + 946,92420961 | + |  |  | y = -0,01377170x + 3 784,57106492 | + |
|  | R² = 0,00007214 |  |  |  | R² = 0,03048488 |  |
| K420 2868 | y = -0,00155940x + 1 371,70036664 | + | y = -0,00058785x + 2 221,11655595 | + |  |  |
|  | R² = 0,15442664 |  | R² = 0,11096634 |  |  |  |
| K421 3064 | y = -0,00388965x + 1 178,79037212 | + |  |  |  |  |
|  | R² = 0,48829268 |  |  |  |  |  |
| K422 3078 | y = 0,00152069x + 533,76415749 | + | y = 0,00798810x + 1 253,85867469 | + | y = 0,05644633x + 531,54969636 | + |
|  | R² = 0,52439207 |  | R² = 1,00000000 |  | R² = 1,00000000 |  |
| K422 3190 secretary | y = 0,00222826x + 761,74321072 | + | y = 0,00602295x + 801,17182326 | 0.006 |  |  |
|  | R² = 0,06434021 |  | R² = 0,80574378 |  |  |  |
| K422 3190 Christina | y = -0,00260217x + 489,57569264 | + | y = 0,09541303x + 9 263,89126996 | + | y = 0,00484719x + 7 174,75116077 | + |
|  | R² = 1,00000000 |  | R² = 1,00000000 |  | R² = 1,00000000 |  |
| K423 3234 | y = 0,00732988x + 200,16682630 | + |  |  |  |  |
|  | R² = 0,72666566 |  |  |  |  |  |
| K429 3767 | y = 0,00070179x + 614,34363122 | + | y = 0,01272750x + 1 352,89671782 | 0.013 | y = 0,00705445x + 723,11309556 | 0.009 |
|  | R² = 0,07563527 |  | R² = 0,94291173 |  | R² = 0,96967585 |  |
| K429 3800 | y = 0,00766359x - 22,41409811 | 0.009 | y = 0,00491960x + 1 186,92566104 | 0.005 |  |  |
|  | R² = 0,94940559 |  | R² = 0,86225679 |  |  |  |
| K429 3801 | y = 0,00312900x - 100,82374597 | 0.004 |  |  |  |  |
|  | R² = 0,92442060 |  |  |  |  |  |
| K430 3842 | y = 0,00298320x + 162,49114357 | 0.003 | y = 0,01657573x + 33,06105746 | 0.017 |  |  |
|  | R² = 0,85209055 |  | R² = 0,95423265 |  |  |  |

## Results of ink ratios based on Compton normalisation quantification

Table showing weight/weight ratios of Mn, Cu, Zn in relation to Fe for all letters measured (+ means that peaks are present but Compton ratios gave low quality regression lines with R^2^ < 0.8 or only two points were measured, hence regression lines would be invalid. Blank means that no peaks were present in the XRF spectra.)

| **Letter** | Slope for Mn/Compton versus Fe/Compton | Slope[(Mn/Comp)/(Fe/Comp)] * [(Slope_concMn/Comp)/(Slope_concFe/Comp)] | Slope for net area Cu versus net area Fe | Slope[(Cu/Comp)/(Fe/Comp)] * [(Slope_concCu/Comp)/(Slope_concFe/Comp)] | Slope for net area Zn versus net area Fe | Slope[(Zn/Comp)/(Fe/Comp)] * [(Slope_concZn/Comp)/(Slope_concFe/Comp)] |
| --- | --- | --- | --- | --- | --- | --- |
|  | **w/w ratio Mn/Fe** | | **w/w ratio Cu/Fe** | | **w/w ratio Zn/Fe** | |
| Del Monte Vol II A1 No 17 | y = 0.00197833x + 0.00663461 | + |  |  |  |  |
|  | R² = 0.23236750 |  |  |  |  |  |
| K394 0014 | y = 0.00699916x + 0.01882196 | + | y = 0.09779068x + 0.00300457 | 0.097 | y = 0.09688859x + 0.02022184 | 0.118 |
|  | R² = 0.13700512 |  | R² = 0.99844740 |  | R² = 0.97644758 |  |
| K396 266 | y = 0.00679011x - 0.00068377 | 0.008 | y = 0.01714911x - 0.00434778 | 0.017 |  |  |
|  | R² = 0.97523235 |  | R² = 0.85536803 |  |  |  |
| K397 364 | y = 0.01903531x + 0.00387688 | 0.021 |  |  | y = 0.02770357x + 0.00073132 | 0.034 |
|  | R² = 0.88575600 |  |  |  | R² = 0.91594767 |  |
| K399 470 | y = 0.00717059x + 0.00298658 | + |  |  |  |  |
|  | R² = 0.62500596 |  |  |  |  |  |
| K401 658 | y = 0.01120544x + 0.00170545 | 0.013 | y = 0.01193791x + 0.00489306 | 0.012 | y = 0.01336289x + 0.01078491 | 0.016 |
|  | R² = 0.96932500 |  | R² = 0.94241370 |  | R² = 0.98193960 |  |
| K403 828 Azzolino | y = 0.00494336x + 0.00224633 | 0.006 |  |  |  |  |
|  | R² = 0.99904970 |  |  |  |  |  |
| K403 828 Christina | y = -0.00870620x + 0.01003563 | + |  |  |  |  |
|  | R² = 0.90464734 |  |  |  |  |  |
| K405 1092 | y = 0.01819946x + 0.00088749 | 0.020 |  |  |  |  |
|  | R² = 0.88185858 |  |  |  |  |  |
| K407 1298 | y = 0.00500455x + 0.00072972 | 0.006 | y = 0.00578363x + 0.00696571 | 0.006 | y = 0.00478385x + 0.00706304 | 0.006 |
|  | R² = 0.96098356 |  | R² = 0.99809530 |  | R² = 0.99350691 |  |
| K408 1556 secretary | y = 0.00600746x + 0.00460441 | 0.007 | y = 0.00217034x + 0.01395054 | 0.002 |  |  |
|  | R² = 0.99961413 |  | R² = 0.93733496 |  |  |  |
| K408 1556 Christina | y = 0.00936706x - 0.00043145 | 0.011 | y = 0.02264099x + 0.00480784 | 0.023 |  |  |
|  | R² = 0.98345710 |  | R² = 0.99690789 |  |  |  |
| K409 1608 | y = 0.00325819x + 0.00420247 | + | y = 0.13786283x + 0.00856613 | 0.137 | y = 0.01117319x + 0.01125600 | 0.014 |
|  | R² = 0.43326097 |  | R² = 0.99808712 |  | R² = 0.92690091 |  |
| K412 1902 | y = 0.01183382x + 0.00183638 | 0.013 |  |  | y = 0.01690228x + 0.05214132 | + |
|  | R² = 0.88902801 |  |  |  | R² = 0.12040546 |  |
| K415 2098 | y = 0.00865355x + 0.00143962 | + | y = 0.04667302x - 0.00370769 | + |  |  |
|  | R² = 0.80684292 |  | R² = 0.76712178 |  |  |  |
| K415 2182 | y = 0.01408313x - 0.00399229 | 0.016 | y = 0.00993128x + 0.00897771 | 0.010 |  |  |
|  | R² = 0.99736297 |  | R² = 0.99151945 |  |  |  |
| K419 2574 | y = 0.01506917x + 0.00262766 | + |  |  | y = 0.03183993x + 0.01404880 | + |
|  | R² = 0.48561150 |  |  |  | R² = 0.46172837 |  |
| K420 2868 | y = 0.00272085x + 0.00741084 | + | y = -0.00240550x + 0.02227819 | + |  |  |
|  | R² = 0.27680596 |  | R² = 0.14530186 |  |  |  |
| K421 3064 | y = -0.00267715x + 0.00642468 | + |  |  |  |  |
|  | R² = 0.49704665 |  |  |  |  |  |
| K422 3078 | y = 0.00094337x + 0.00579376 | + | y = 0.00980559x + 0.00584551 | + | y = 0.04797230x + 0.01251853 | + |
|  | R² = 0.09938554 |  | R² = 1.00000000 |  | R² = 1.00000000 |  |
| K422 3190 secretary | y = 0.00586904x - 0.00505501 | + | y = 0.00821939x - 0.00025548 | 0.008 |  |  |
|  | R² = 0.74507824 |  | R² = 0.97044085 |  |  |  |
| K422 3190 Christina | y = -0.00048014x + 0.00204715 | + | y = 0.13556682x + 0.03873667 | + | y = 0.03594572x + 0.03000099 | + |
|  | R² = 1.00000000 |  | R² = 1.00000000 |  | R² = 1.00000000 |  |
| K423 3234 | y = 0.00632222x + 0.00375845 | + |  |  |  |  |
|  | R² = 0.64292567 |  |  |  |  |  |
| K429 3767 | y = 0.00164379x + 0.00300619 | + | y = 0.01573404x + 0.00480205 | 0.016 | y = 0.00726014x + 0.00474171 | 0.009 |
|  | R² = 0.27744871 |  | R² = 0.80075756 |  | R² = 0.96802671 |  |
| K429 3800 | y = 0.00774127x - 0.00021507 | 0.009 | y = 0.00429012x + 0.00876290 | 0.004 |  |  |
|  | R² = 0.95248481 |  | R² = 0.57038354 |  |  |  |
| K429 3801 | y = 0.00299461x - 0.00060414 | 0.003 |  |  |  |  |
|  | R² = 0.86608272 |  |  |  |  |  |
| K430 3842 | y = 0.00257753x + 0.00286503 | 0.003 | y = 0.01441858x + 0.00480031 | 0.014 |  |  |
|  | R² = 0.97655632 |  | R² = 0.99602730 |  |  |  |

# Discussion and conclusions

- Quantification of the following elements was attempted: iron, manganese, copper and zinc. Quantification of other elements was not attempted; several were shown to be present in all or some of the inks and include sulphur, potassium, calcium, mercury and lead.
- The net counts quantification results showed iron levels up to 48 mg/g paper, manganese levels up to 0.11 mg/g paper, copper levels up to 2.10 mg/g paper and zinc levels up to 1.27 mg/g paper.
- The Compton ratios quantification results showed iron levels up to 53 mg/g paper, manganese levels up to 1.05 mg/g paper, copper levels up to 0.87 mg/g paper and zinc levels up to 0.57 mg/g paper.
- The graphs below show comparisons of the two quantification methods indicating that there are discrepancies between the calculated amounts of metal in paper. Furthermore, for both quantification methods almost all of the manganese, copper and zinc quantification results showed negative values. This implies that neither quantification method is accurate enough to be applicable to such low levels. The levels of Mn, Cu and Zn measured in the Azzolino collection inks are below or at the lower end of those in the reference metal impregnated papers.
- The ink ratios giving weight by weight content of manganese, copper and zinc in relation to iron were calculated from the quantification slopes using both methods and yielded very similar results for ink compositions. The highest amounts calculated were ca. 21 mg of manganese per g iron, 137-141 mg copper per g iron and 118-125 mg zinc per g iron. The bar charts below show the results from both sets of calculations. However, only few of the letters analysed could be used for this type of quantification as data could not be included if regression lines were of low quality or too few points were measured, see the relevant tables in the results section.
- Therefore, it was decided not to use the quantification methods for a comparison of XRF results of the inks on the analysed letters. Instead, Compton ratios of net peak areas were chosen because the normalisation helped to reduce some of the inhomogeneity between data that is introduced due to variations in detector deadtime, differences in paper types and other matrix effects.

### Net peak area versus Compton normalisation for quantification of concentration metal/paper (mg/g)


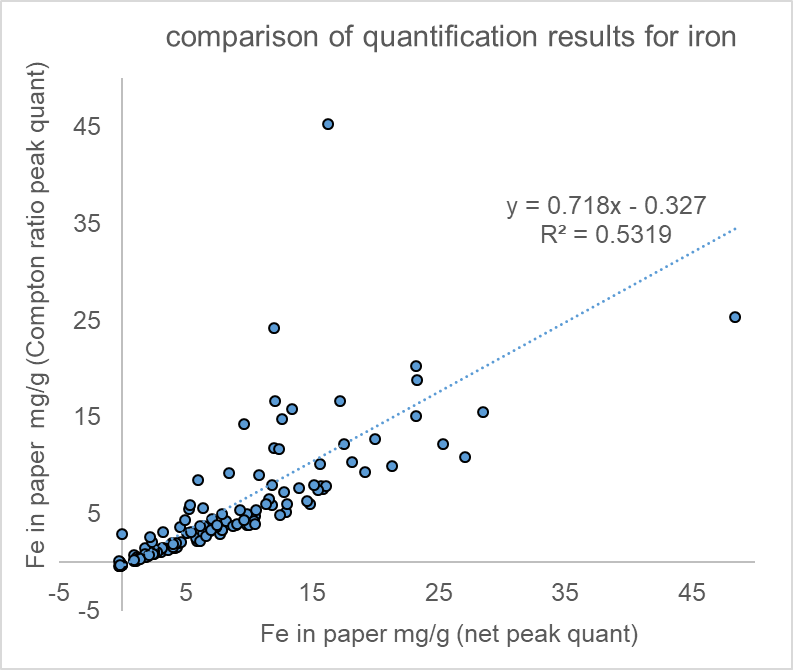

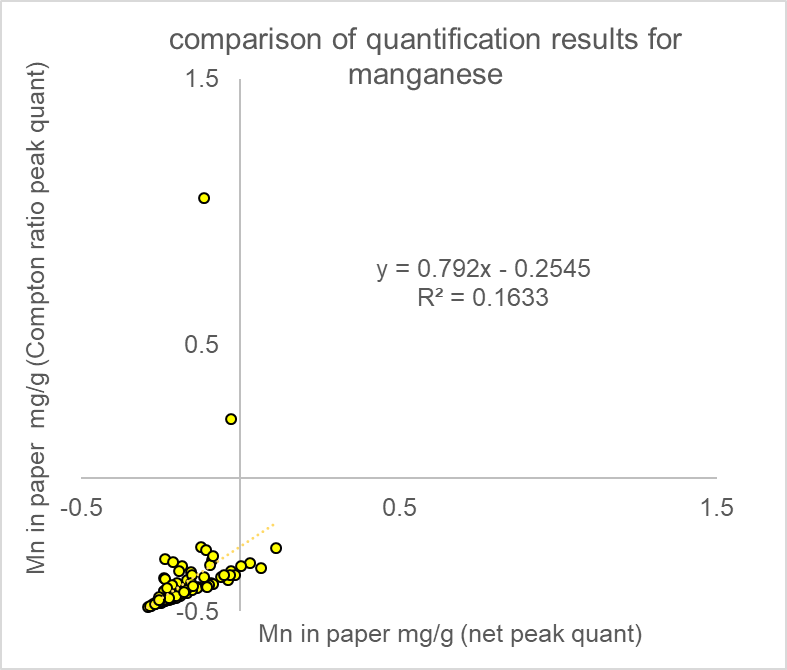


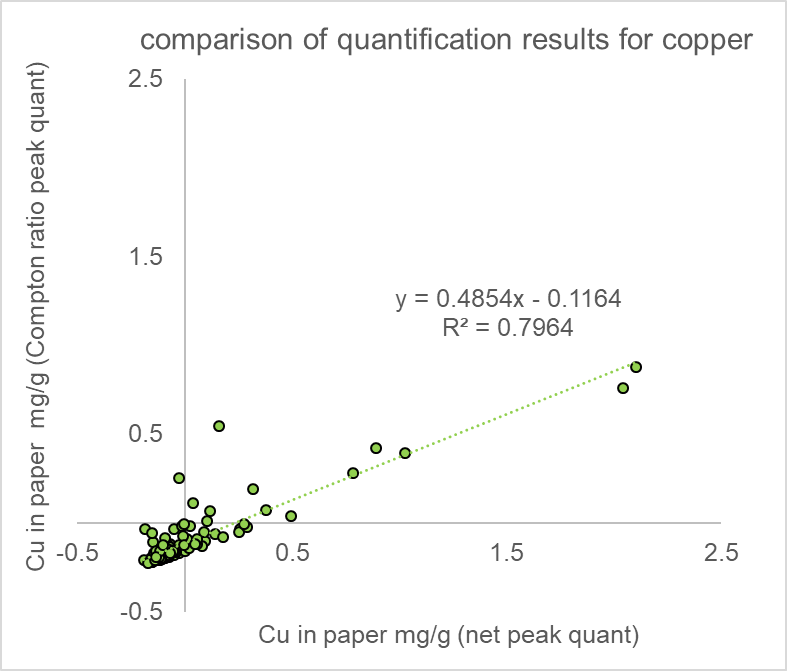

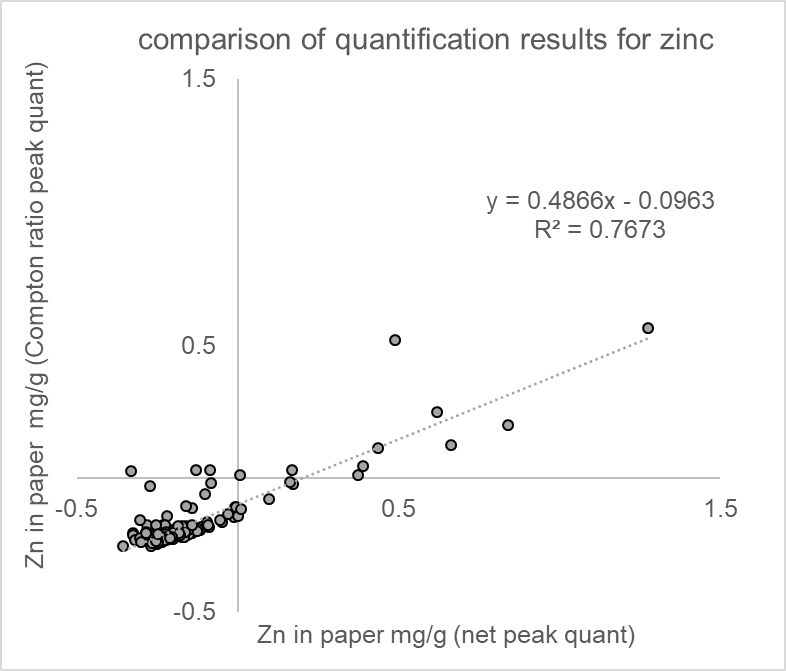


### Ink ratios calculated from quantification slopes giving weight/weight content of manganese, copper and zinc in relation to iron


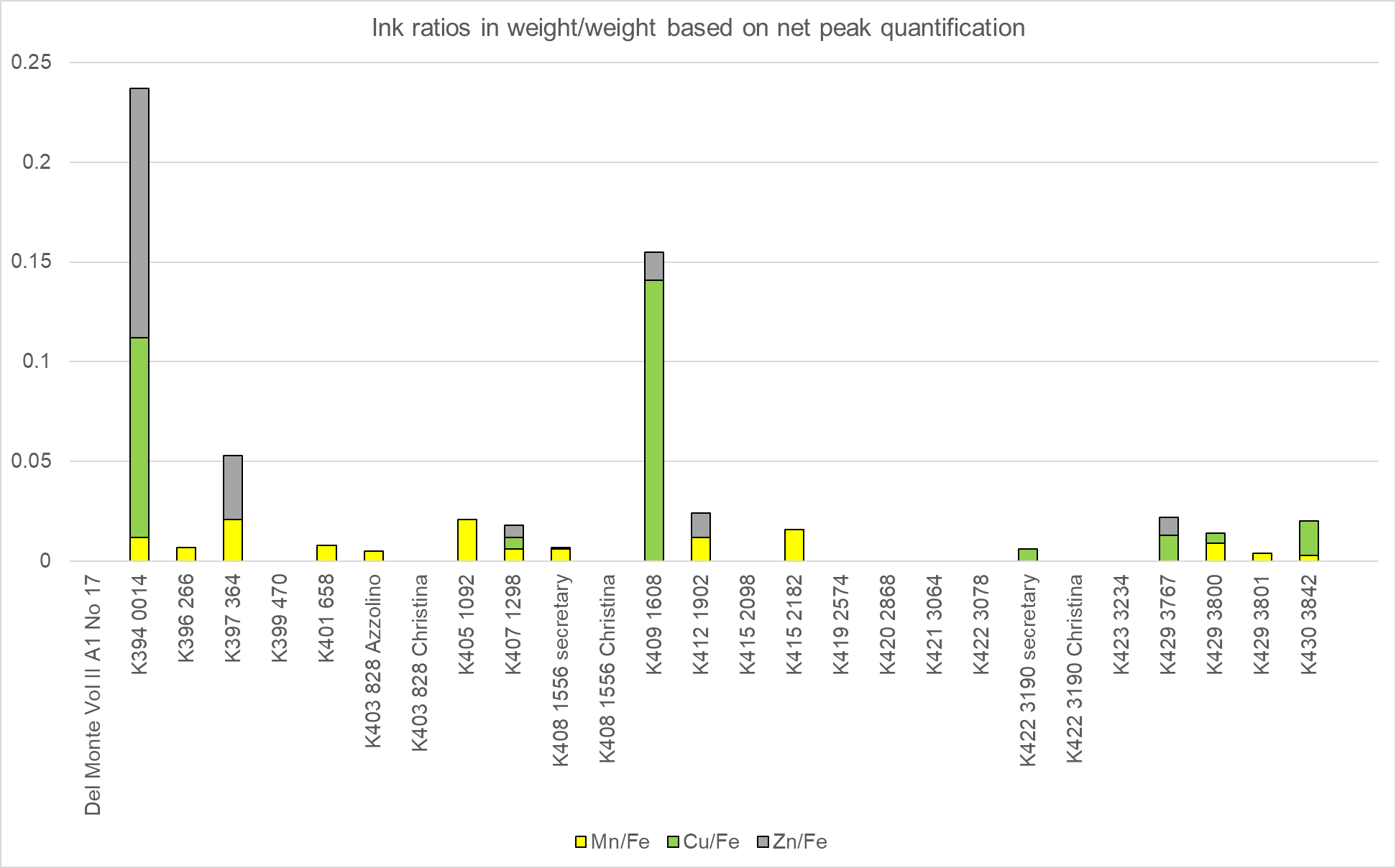


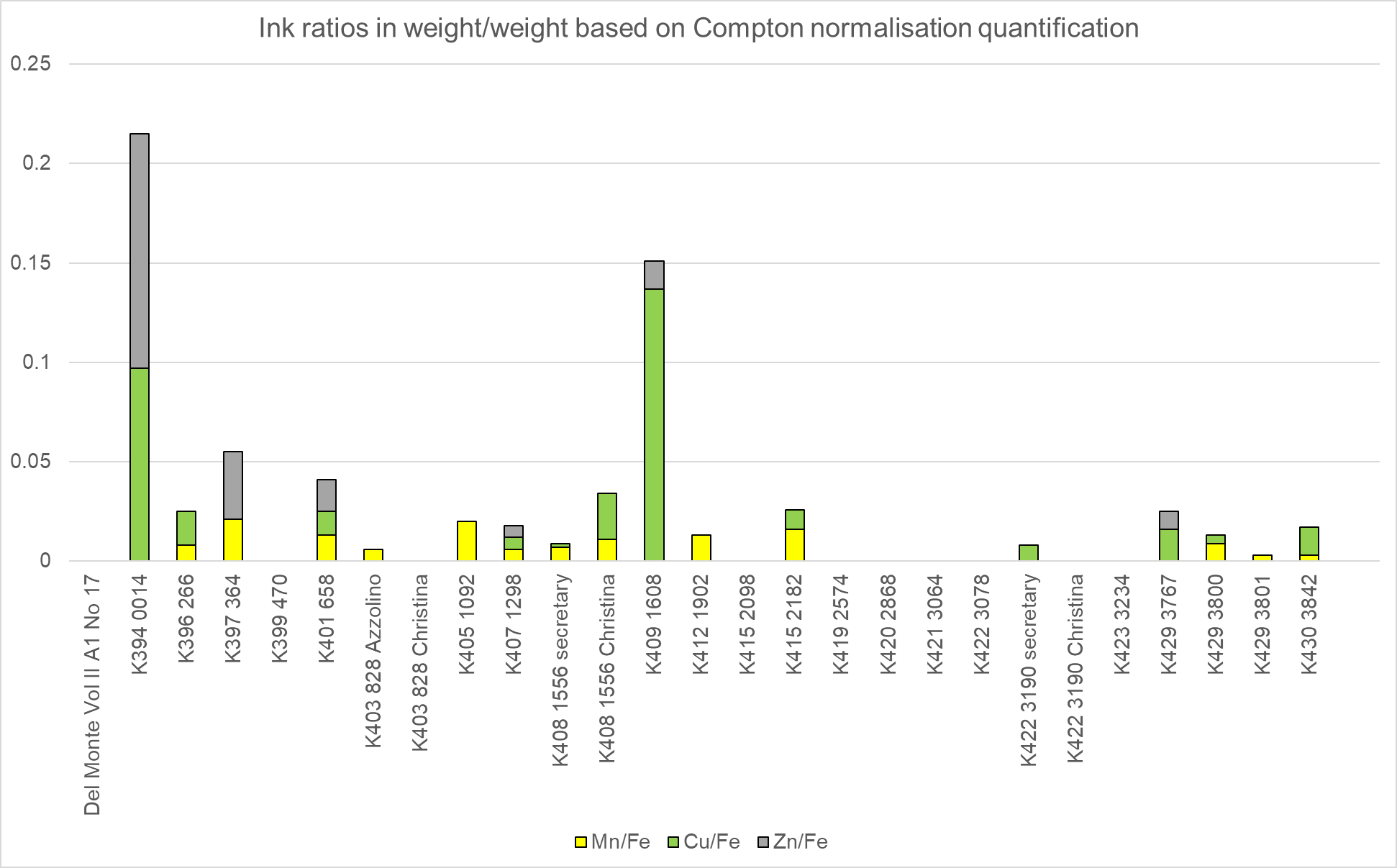

Supplement: S3 File — Report on quantification method based on XRF analysis. (DOCX) [file pone.0283539.s003.docx]
